# Hierarchically Micro‐ and Mesoporous Zeolitic Imidazolate Frameworks Through Selective Ligand Removal

**DOI:** 10.1002/smll.202307981

**Published:** 2023-12-21

**Authors:** Zheao Huang, Jakob Rath, Qiancheng Zhou, Alexey Cherevan, Shaghayegh Naghdi, Dominik Eder

**Affiliations:** ^1^ Institute of Material Chemistry Vienna University of Technology Vienna 1060 Austria; ^2^ Institute of Nanoscience and Nanotechnology College of Physical Science and Technology Central China Normal University Wuhan 430079 China

**Keywords:** cluster defects, hierarchically porous ZIF, mesopores, selective ligand removal, water purification

## Abstract

A new method to engineer hierarchically porous zeolitic imidazolate frameworks (ZIFs) through selective ligand removal (SeLiRe) is presented. This innovative approach involves crafting mixed‐ligand ZIFs (ML‐ZIFs) with varying proportions of 2‐aminobenzimidazole (NH_2_‐bIm) and 2‐methylimidazole (2‐mIm), followed by controlled thermal treatments. This process creates a dual‐pore system, incorporating both micropores and additional mesopores, suggesting selective cleavage of metal‐ligand coordination bonds. Achieving this delicate balance requires adjustment of heating conditions for each mixed‐ligand ratio, enabling the targeted removal of NH_2_‐bIm from a variety of ML‐ZIFs while preserving their inherent microporous framework. Furthermore, the distribution of the initial thermolabile ligand plays a pivotal role in determining the resulting mesopore architecture. The efficacy of this methodology is aptly demonstrated through the assessment of hierarchically porous ZIFs for their potential in adsorbing diverse organic dyes in aqueous environments. Particularly striking is the performance of the 10%NH_2_‐ZIF‐2 h, which showcases an astonishing 40‐fold increase in methylene blue adsorption capacity compared to ZIF‐8, attributed to larger pore volumes that accelerate the diffusion of dye molecules to adsorption sites. This versatile technique opens new avenues for designing micro/mesoporous ZIFs, particularly suited for liquid media scenarios necessitating efficient active site access and optimal diffusion kinetics, such as purification, catalysis, and sensing.

## Graphical abstract

The research focuses on the construction of mixed‐ligand and ligand‐removal zeolitic imidazolate frameworks ZIF‐8, followed by the ligand thermolysis strategy to selectively remove thermolabile amino‐ligands in order to design controllable hierarchical pores that provide larger pore volumes and facilitating faster transport of the dye molecules to the adsorption sites.

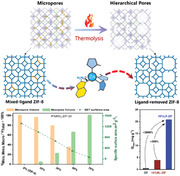

## 1 Introduction

Zeolitic imidazolate frameworks (ZIFs) are a special sub‐class of metal‐organic frameworks (MOFs) constructed from tetrahedral metal ions (e.g. Zn, Co) bridged by imidazolate (IM) ligands.^[^ ^1^ ^]^ ZIF‐8 is a popular representative that is composed of tetrahedrally coordinated zinc ions based on zeolitic sodalite topology.^[^ ^2^ ^]^ The presence of microporosity (pore size < 2 nm) has allowed ZIF‐8 to be extensively researched in adsorption,^[^ ^3^ ^]^ catalysis,^[^ ^4^ ^]^ drug release^[^ ^5^ ^]^ and hydrogen storage.^[^ ^6^ ^]^ ZIFs were initially regarded as structurally ideal materials with tunable micropores. However, as research has progressed, the lack of mesoscale cavities has shown to impede the diffusion/adsorption of macromolecules (e.g. enzymes, drugs and particulate matter),^[^ ^7^, ^8^, ^9^, ^10^ ^]^ which severely restricts the expansion of ZIFs’ application. Thus, the incorporation of mesopores (2–50 nm) in parent ZIFs is crucial in order to meet the requirements of modern applications.

Recent years have seen an increasing number of attempts at designing hierarchically porous MOFs (HP‐MOFs), which consist of both micro‐ and mesopores, often with interconnected channels.^[^ ^11^, ^12^ ^]^ HP‐MOFs are able to retain the advantages of intrinsic micropores, yet the presence of additional mesoscale cavities expands their potential applications. In addition to reducing the restriction of macromolecule diffusion, the existence of hierarchical pores also exposes coordinatively unsaturated sites, facilitating enhanced catalysis and adsorption performance.^[^ ^10^, ^12^, ^13^ ^]^ Consequently, many synthetic strategies have been developed to design and construct HP‐MOFs such as commonly used templating,^[^ ^14^ ^]^ defect formation strategy,^[^ ^15^ ^]^ and etching technique.^[^ ^16^ ^]^ While mesoporous structures can originate from the development of defects, excessively random defects make it difficult to precisely tailor the mesoporous features in terms of molecular size. Furthermore, the template removal and etching processes often result in morphological or structural collapse, which can have detrimental effect on the crystal structure and relevant properties.^[^ ^17^ ^]^ In addition to these bottlenecks, it remains a challenge to control size and spatial arrangement of the hierarchical pores, not only for the stability of the overall MOF framework, but likewise for maintaining the integrity of the mesoscale cavities.^[^ ^18^, ^19^, ^20^ ^]^

A more promising strategy toward HP‐MOFs was recently reported by Feng et al.^[^ ^21^ ^]^ and Naghdi et al.^[^ ^13^ ^]^] and is based on the selective ligand removal (SeLiRe) from mixed‐ligand MOFs through thermolysis. This strategy is a post‐synthetic method, which makes it applicable to a wider range of MOFs. Moreover, the removal of the thermolabile ligand can result in the formation of different mesoporous architecture, e.g. channels and cavities, making it easier to control the porosity of HP‐MOFs while maintaining the intrinsic crystal structure. So far, however, the construction of hierarchical pores via SeLiRe has not been achieved with ZIFs.

Herein, we utilize the SeLiRe strategy for the first time to construct ZIFs with controlled hierarchical micro/mesopores. Distinct functional groups are known to cause considerable differences in the ligand's resistance to thermal treatments^[^ ^21^, ^22^ ^]^ Therefore, the first step involves the synthesis of mixed‐ligand ZIFs (ML‐ZIFs) with suitably distinct thermal stability. In this work, we replaced different fractions of the original 2‐mIm ligand with the more thermolabile amino‐functionalized NH_2_‐bIm ligand. The second step involves the selective removal of NH_2_‐bIm through careful cleavage of the metal‐ligand coordination. In contrast to the aforementioned MOF examples, the SeLiRe process with ZIFs requires a more careful fine‐tuning of various process conditions including temperature, atmosphere and reaction time. The process was monitored through various in situ techniques, and the ligand removal was quantified through ATR‐IR and ^1^H NMR spectroscopies. In the third step, ligand‐removed ZIFs (LR‐ZIFs) were assessed to have controlled micro/mesoporosity by N_2_ physisorption. Ultimately, we show that the hierarchically porous structure of the new LR‐ZIFs can significantly enhance the adsorption capacity of various organic dyes in water.

## 2 Results and Discussion

### 2.1 Structural Characterization of Mixed‐Ligand ZIFs

**Figure 1:**
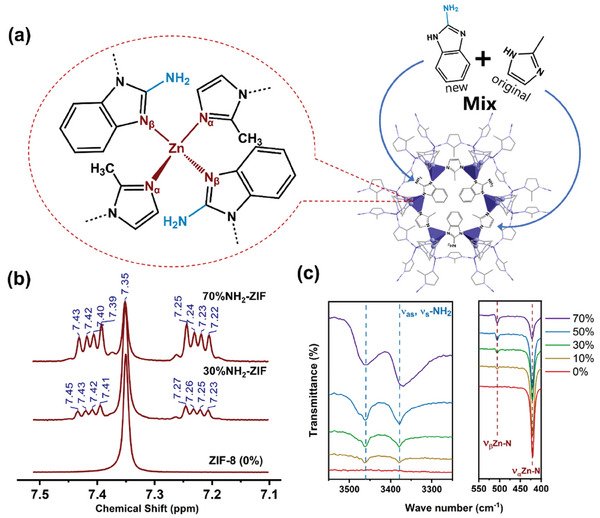
Structural characterization of mixed‐ligand ZIFs. Schematic diagram of the ZnN_4_ cluster and unit cell a) of the 50%NH_2_‐ZIF. ^1^H NMR spectroscopy of pure ZIF‐8, 30%NH_2_‐ZIF and 70%NH_2_‐ZIF b), from the entire ^1^H NMR in Figure S1 (Supporting Information). All samples were digested using d^4^‐acetic acid and then tested by ^1^H NMR. Ex situ ATR‐IR spectra of 0–70%NH_2_‐ZIFs, presented for the IR range at 400–550 cm^−1^ and 3250–3550 cm^−1^ respectively c), from the entire IR spectra in Figure S2a (Supporting Information).

ZIF‐8 is formed by the tetrahedral coordination of zinc ions linked to the ligand 2‐mIm.^[^ ^23^, ^24^ ^]^ Mixing in the thermolabile ligand, NH_2_‐bIm (**Figure** **1**a), is a typical method for introducing amino groups.^[^ ^25^ ^]^ We synthesized a series of R%NH_2_‐ZIFs through solvent‐assisted ligand exchange (SALE), where R% represents the synthetic mass of the NH_2_‐bIm ligand (0–70%). In the ^1^H nuclear magnetic resonance (^1^H NMR) of Figure 1b, the ML‐ZIFs (30% and 70%) exhibit the typical spectral features of both 2‐mIm and NH_2_‐bIm (characteristic quartets on sides of 7.35 ppm), compared to pure ZIF‐8. The quartets increase with increasing R%, which allows quantification of the actual mixed‐ligand composition by ^1^H NMR. Figure S1 and Table S1 (Supporting Information) show that the respective contents are in good agreement with the nominal values.

Attenuated total reflection infrared spectroscopy (ATR‐IR) reveals the successful embedding of NH_2_‐bIm ligands within the ML‐ZIF framework. Notably, an increase in the mixing ratio of NH_2_‐bIm is associated with elevated ν(‐NH_2_) modes (symmetrical at 3380 cm^−1^ and asymmetric at 3460 cm^−1^, as seen in Figure 1c; Figure S2a, Supporting Information), indicating homogeneous incorporation of NH_2_‐bIm.^[^ ^25^, ^26^ ^]^ Additionally, a new band emerges at 506 cm^−1^ (Figure 1c), intensifying with higher NH_2_‐bIm fractions within the structure. Correspondingly, the original ν(Zn─N) mode (at 421 cm^−1^) associated with metal‐IM bonds diminishes. Importantly, the 506 cm^−1^ band is independent of NH_2_‐bIm itself (Figure S2b, Supporting Information) and closely resembles the Zn─N band observed in ZIF‐7 synthesized using a benzimidazole ligand (bIm) with a structure akin to NH_2_‐bIm.^[^ ^27^, ^28^ ^]^

The data therefore suggest that the new band is generated by the new Zn─N bonding vibration between Zn and N on the fused‐imidazole ring on NH_2_‐bIm (hence, the original Zn─N is named Zn‐N_α_ and the new one is named Zn‐N_β_). Note that the original tetrahedral coordination of Zn^2+^ is maintained (Figure 1a). The decrease in ν(Zn‐N_α_) band and the increase in ν(Zn‐N_β_) band are consistent with the increasing mixed‐ligand ratio, thus reflecting the competitive coordination between 2‐mIm and NH_2_‐bIm (Figure S2c,d, Supporting Information).

The X‐ray diffraction (XRD) patterns of the ML‐ZIFs exhibit a close resemblance to those of ZIF‐8, characterized by multiple Bragg peaks, indicating well‐preserved intrinsic structure and good crystallinity (Figure S3a, Supporting Information). As the ligand ratio increases, the intensity of the crystalline (011) peak diminishes, accompanied by an increase in its full‐width at half‐maximum (FWHM). This indicates the presence of structural distortion due to the competitive coordination between the new amino ligand and the original ligand, resulting in a weakening of the crystallinity (Figure S3b, Supporting Information).^[^ ^29^, ^30^ ^]^ Consequently, this phenomenon introduces some degree of disorder, impacting the morphology and the “optimal” particle size, causing the ZIFs to exhibit less stable growth compared to the prior state (as observed in scanning electron microscopy, SEM, in Figure S4, Supporting Information).

### 2.2 Influence of Temperature and Time on Selective Ligand Removal

The thermal stability of the samples ranging from 0% to 70% of NH_2_‐bIm content was examined through thermogravimetric analysis (TGA) under air atmosphere, heating up to 600 °C. The TGA curves reveal distinct mass losses for the ML‐ZIFs within the temperature range of 200–400 °C (Figure S5a, Supporting Information). In the case of the 0–50% samples, the initial mass loss ≈200 °C appears relatively consistent, but a noticeable divergence becomes apparent ≈350 °C compared to the pure ZIF‐8 (0%). This divergence becomes more pronounced in the 70% sample, where the mass loss is more substantial and initiates at lower temperatures. This heightened mass loss is attributed to the poorer thermal stability (200 °C) and excessive water evaporation (25 °C).^[^ ^29^, ^31^ ^]^

The significant reduction in mass observed at temperatures exceeding 400 °C corresponds to the structural breakdown of the ZIFs framework due to ligand oxidation. Additionally, TGA analysis under isothermal heating conditions provides a clearer depiction, illustrating that the mass loss of ML‐ZIFs at 300 °C progressively rises with increasing NH_2_‐bIm content (Figure S5b,c, Supporting Information). This observation suggests a quantitative correlation between the extent of mass loss during heating and the concentration of the NH_2_‐bIm ligand incorporated in the corresponding ML‐ZIF structure.

**Figure 2:**
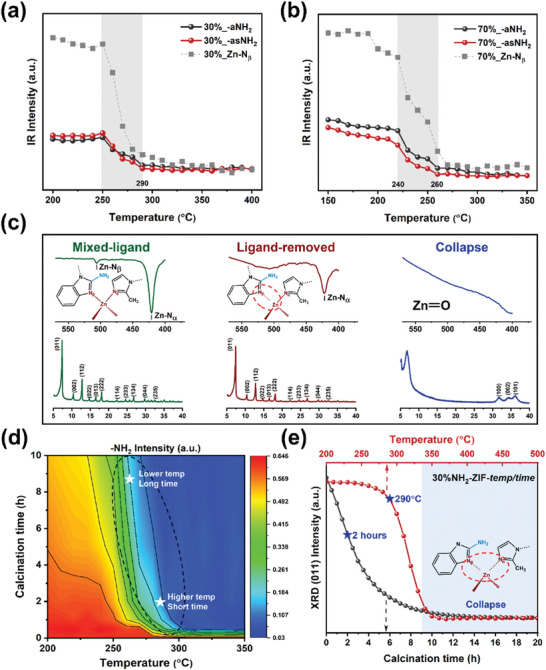
The effects of heating temperature and duration on selective ligand removal. Evolution of the ν(‐NH_2_) and ν(Zn‐N_β_) bands extracted from ex‐situ AIR‐IR for 30%NH_2_‐ZIF a) and 70%NH_2_‐ZIF b) upon heating in air, monitoring the selective removal of NH_2_‐bIm. Ex situ ATR‐IR spectra and in situ XRD of 30%NH_2_‐ZIF at room temperature, 290 and 400 °C c). 2D contour plot of ν(‐NH_2_) intensity in ML‐ZIFs versus calcination temperature and duration d). Data are extracted from the IR spectra in Figures S6–S8 (Supporting Information) at 3250–3550 cm^−1^ for ν(‐NH_2_), 400–550 cm^−1^ for ν(Zn─N). Evolution intensity plot of (011) peak with calcination temperature/duration in the in‐situ XRD of 30%NH_2_‐ZIF e) in air. Data are extracted from the XRD patterns in Figures S13 and S14 (Supporting Information).

Further analysis was conducted on all samples using ATR‐IR after subjecting them to varying temperatures and durations of thermal treatment (details summarized in Figures S6–S8, Supporting Information). Changes in intensity for bands associated with NH_2_‐bIm, including *ν*_s_(‐NH_2_) and *ν*_as_(‐NH_2_), as well as ν(Zn‐N_β_), were observed, as shown in **Figure** **2**a,b. Notably, in samples ranging from 10% to 50%, these bands exhibit reduction between 250 and 290 °C, while for the 70% samples, the reduction occurs already within the range of 240 to 260 °C. In all the cases, however, the bands such as ν(Zn‐N_α_), corresponding to 2‐mIm, remain intact within these temperature ranges, indicating its higher thermal stability compared to NH_2_‐bIm (IR spectra in Figure 2c).

Consistent with observations in ^1^H NMR spectroscopy, the distinctive quartets of NH_2_‐bIm experience significant reduction following ligand thermolysis, with their chemical shifts becoming barely discernible after 2 h (Figure S9, Supporting Information). Since the amino group is unique to NH_2_‐bIm, the decline in ν(‐NH_2_) due to temperature can be attributed exclusively to the removal of NH_2_‐bIm. Likewise, the decrease in ν(Zn‐N_β_) with increasing heating temperature and duration confirms the quantitative removal of NH_2_‐bIm from the organic framework through thermolysis, involving cleavage of the Zn‐N_β_ bond.

The intricate interplay between calcination temperature and duration is visually depicted through 2D contour plots, aiding in the identification of specific parameters for achieving quantitative selective ligand removal (Figure 2d; Figure S10, Supporting Information). For instance, in the case of the 30% sample, either a prolonged exposure at lower temperature (e.g. 260 °C for 8 h) or a shorter duration at higher temperature (e.g., 290 °C for 2 h) is effective in selectively removing the NH_2_‐bIm ligand. In samples with higher ratios (e.g., 70%), even lower heating conditions suffice (Figures S11,S12, Supporting Information). However, it is important to note that while thermolysis effectively removes NH_2_‐bIm, it may also compromise the inherent framework structure of ZIF‐8. This non‐desirable impact on the metal‐organic framework underscores the need for meticulous precision when defining the heating parameters.

The influence of ligand removal on framework alterations was explored through in situ X‐ray diffraction (in situ XRD). The comprehensive diffraction patterns for all samples are summarized in Figures S13 and S14 (Supporting Information). Figure 2e highlights the dynamic changes in the intensity of the (011) peak for the 30% sample at different temperatures and durations. The data indicate that no observable changes occur at heating temperatures and durations below 290 °C and 2 h. Beyond these thresholds, the inherent structure and crystallinity of ZIF‐8 progressively deteriorate, eventually resulting in the emergence of peaks corresponding to hexagonal wurtzite ZnO. Similar observations can be extended from the 2D/3D contour plots of other ML‐ZIFs, with the vanishing of the (011) peak accelerating as the proportion of thermolabile NH_2_‐bIm increases, signifying a diminishing thermal stability of ML‐ZIFs (Figure S15, Supporting Information).

It's noteworthy that the effective temperature ranges for achieving selective ligand removal in ML‐ZIFs are relatively narrow, spanning ≈40–50 °C. This range contrasts with the broader window of ≈150 °C found in traditional MOFs containing amino‐terephthalic acid ligands (e.g., NH_2_‐MIL‐125 and NH_2_‐UiO‐66).^[^ ^13^, ^21^, ^32^ ^]^ The narrow thermolysis temperature window for both ligands, makes it challenging to selectively remove a particular ligand in this system. For instance, in the case of ZIF‐67, which is a cobalt‐based analog of ZIF‐8, our attempts were unsuccessful primarily due to the low thermal stability of the Co─N bond within ZIF‐67 (Figures S16–S18 and Section S2, Supporting Information).

Hence, the collective findings from TGA, ATR‐IR, ^1^H NMR, and XRD analyses validate that subjecting the ML‐ZIFs to a 2 h thermal process at 260 °C (for 70% composition) and 290 °C (for 10–50% compositions) achieves the precise elimination of NH_2_‐bIm while maintaining the integrity of the framework structure.

### 2.3 Characterization of Pore Structures in LR‐ZIFs

**Figure 3:**
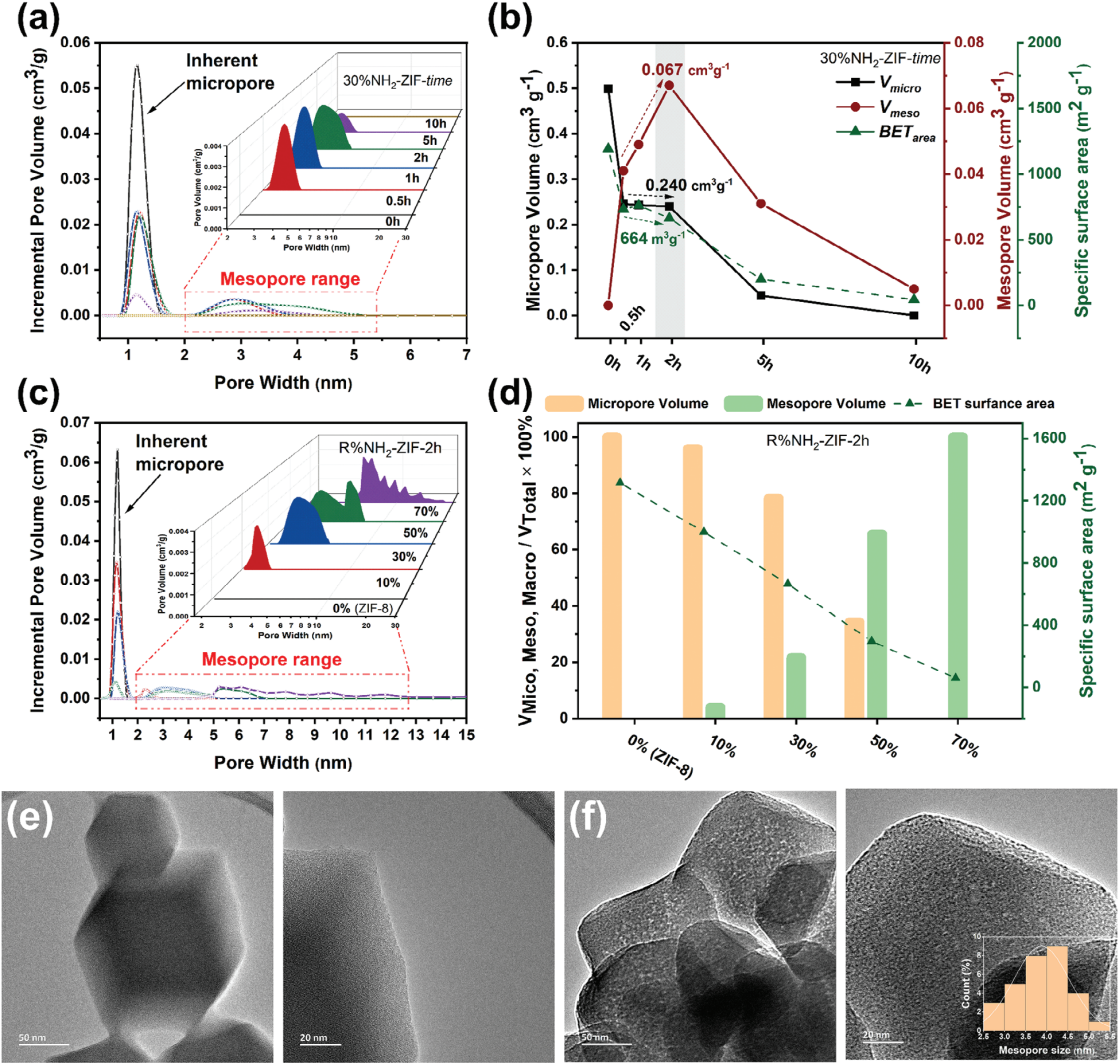
Hierarchical pores in LR‐ZIFs. NLDFT porosity distributions of 30%NH_2_‐ZIF‐time a) and R%NH_2_‐ZIF‐2 h c). Time‐dependent plot showing the change of micro‐, mesopore volume and BET specific surface area with calcination time from 0–10 h b). Ligand‐dependent histogram showing the change of pore volume ratio and BET specific surface area with mixed‐ligand ratio d). TEM images of 30%NH_2_‐ZIF before e) and after f) calcination 2 h. The distribution of mesopore size is plotted inside (f).

The impact of varying calcination conditions and mixed‐ligand ratios on the pore structure of ligand‐removed ZIFs (LR‐ZIFs) was assessed through N_2_ physisorption at 77 K. Pure ZIF‐8, heated ZIF‐8 (290 °C for 2 h) and ML‐ZIF exhibit type‐I isotherms with no pores exceeding diameters of 2 nm, characteristic of typical microporous materials (**Figures** **3**; Figures S19 and S20, Supporting Information). The situation is different with the LR‐ZIFs. Utilizing nonlocal density functional theory (NLDFT), we observed that the distinct peak centered at 1.2 nm, representative of intrinsic micropores, is largely preserved independent of the initial mixed‐ligand ratio. However, the LR‐ZIFs exhibit additional pores in the range from 2 to 30 nm (Figure 3). Moreover, the volume ratio of micro‐ to mesopores and the size distribution of mesopores are both contingent on the heating duration and initial ligand composition of the samples.

Let us focus first on the influence of heating duration. Figure 3a illustrates that the sample 30%NH_2_‐ZIF (ML‐ZIF) lacks mesopores initially, yet following a 0.5‐h thermolysis, a discernible mesopore emerges measuring ≈2.8 nm in diameter, accompanied by a decrease in micropore volume. Subsequently, the volume and dimensions of the mesopores grow from 0.5 to 2 h, culminating at 2 h with a diameter of ≈3.5 nm and a maximum mesopore volume of 0.067 cm^3^ g^−1^. Importantly, the micropore volume and specific surface area exhibit no pronounced reduction trend during this period, signifying a stable hierarchical structure at the 2 h mark of heating (Figure 3b; Figure S20, Supporting Information). In contrast to this, following a 5‐h calcination, both the specific surface area and micro/mesopore volume experience a notable decline (Table S2, Supporting Information), attributed to partial framework collapse, as confirmed by XRD and IR analyses.

Continuing, we examine the influence of the initial ligand ratio, specifically focusing on a 2‐h thermolysis duration (0–50% at 290 °C, 70% at 260 °C). The findings reveal a significant augmentation in the size distribution of mesopores with escalating NH_2_‐bIm content, spanning from 3–4 nm in the 10% sample to ≈4–25 nm in the 70% sample. This size expansion is concomitant with a reduction in micropore volume ratio (Figure 3c,d). Intriguingly, a more intricate bimodal meso‐porosity distribution is noticeable at 50% (featuring an additional peak ≈6 nm), along with a multimodal distribution at 70%. Furthermore, the 70% sample seems to have lost all the microporous structures, retaining only mesopores distributed within the range of 4–25 nm. These observations imply that the transition of the mesoporous structure tends to be gradual and is influenced by the initial ligand ratio.

Transmission electron microscopy (TEM) images of the 30% sample before and after ligand thermolysis further verify the presence of hierarchical pores (Figure 3e,f). The illuminated regions denote the existence of mesoscale voids that are uniformly arranged throughout the particles, resulting in a sponge‐like porous configuration. The size of these mesopores observed in the TEM images mainly ranges from 3 to 4 nm, further supporting the porosity distribution diagram obtained from N_2_ physisorption. Notably, these illuminated patches are more pronounced at higher ML ligand ratios, underscoring the augmented mesopore dimensions compared to lower ML ratios (Figure S21, Supporting Information). Importantly, these changes caused by SeLiRe do not affect the chemical state and elemental composition of the surface, which is well illustrated by the fact that Zn 2p and N 1s edges are not significantly shifted in the XPS of ZIFs before and after SeLiRe process (Figure S22, Supporting Information). After two hours of heat treatment, analyses of N 1s peaks clearly show a decrease of the C─N/Zn─N ratio from 49.8% to 38.4% for the sample 30%, and from 70.3% to 53.9% for the sample 70%. This direct evidence further confirms the occurrence of SeLiRe process in ML‐ZIFs during the heat treatment.^[^ ^21^ ^]^

### 2.4 Mechanism of Hierarchical Pore Formation

**Figure 4:**
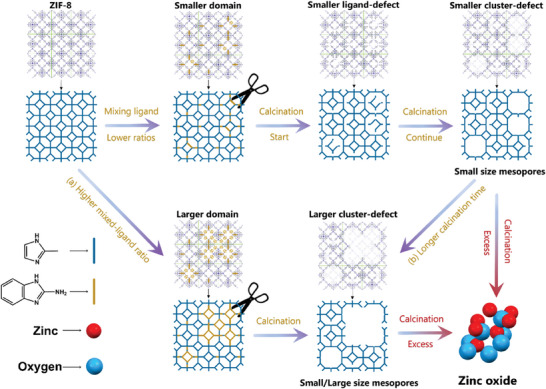
Schematic diagram of the proposed mechanism for the SeLiRe process. The formation of mesopores is guided by the initial distribution of the NH_2_‐bIm ligand that classifies the formation of defects into small and large domains as well as by the chosen conditions for thermolysis. The blue units represent the ligand 2‐mIm, and the yellow units represent the ligand NH_2_‐bIm.

Our research has unveiled a molecular‐scale mechanism governing the formation of hierarchical pores that is intricately linked to the mixed‐ligand ratios (**Figure** **4**). In the context of mixed‐ligand ZIF‐8, it is established that lower concentrations of the thermolabile ligand generally lead to the creation of smaller nanodomains with a random disposition.^[^ ^33^ ^]^ Conversely, at higher NH_2_‐bIm concentrations, the thermolabile ligand has a propensity to aggregate around ZnN_4_ clusters, giving rise to larger domains.^[^ ^33^, ^34^ ^]^

Upon elevation of temperature, the thermolabile NH_2_‐bIm undergoes direct decomposition through cleavage of the Zn─N_β_ bonds, marking the initiation of a one‐step SeLiRe process. Simultaneously, the evaporation of small molecules like NO_x_, CO_2_, and H_2_O occurs,^[^ ^21^, ^35^ ^]^ often accompanied by an intensification of the sample's color (Figure S23, Supporting Information). This process contrasts with the SeLiRe mechanism observed in MOFs featuring the amino‐terephthalic acid ligands, where a two‐step cleavage occurs, and ligand decomposition results solely from the second (delayed) cleavage.^[^ ^13^, ^21^ ^]^ Moreover, in contrast to MOFs, we observed that the SeLiRe process in inert gases (Ar and N_2_) can also be achieved in the case of ML‐ZIFs (Figures S24–S32 and Section S4, Supporting Information), underscoring that the decomposition of NH_2_‐bIm is not ascribable to oxidation and can transpire independently.

In accordance with existing literature findings,^[^ ^36^, ^37^ ^]^ the breaking of Zn─N bonds tends to occur more readily upon heating compared to the breakdown of the imidazole ring structure. As the ligand connections gradually weaken, the count of ZnN_4_ tetrahedral clusters linked to nitrogen starts diminishing (as indicated by the decline curve in Zn‐N_β_ in the IR spectrum). Drawing from our observations, it is reasonable to deduce that these disengaged ZnN_4_ clusters, in conjunction with the adjacent absent ligands, coalesce to form cluster‐defects and additional Zn sites within the frameworks.^[^ ^38^ ^]^ Computational simulations by Zhang et al. provide further support for the thermodynamic feasibility of metal and ligand vacancy formation within the ZIF‐8 framework.^[^ ^39^ ^]^

To validate the structural integrity under this mechanism, we employed density functional theory (DFT) calculations on the LR‐ZIFs framework structure, simulating the removal of various ligand‐clusters. Figures S33 and S34 (Supporting Information) depict the LR‐ZIFs unit cell post the elimination of one, two, and four Zn‐N_4_ clusters, alongside their associated ligands (Section S5, Supporting Information). The outcomes confirm that the fundamental framework structures remain largely intact upon the introduction of mesopores at the molecular level. Furthermore, the similarity in terms of formation energy values of framework with various number of Zn‐N_4_ clusters removed demonstrates the thermodynamic stability of cluster‐defect configurations (Table S3, Supporting Information). The collective findings from XRD, XPS, SEM, TEM, and ATR‐IR underscore that the SeLiRe process exerts minimal impact on the structural integrity, affirming that the LR‐ZIFs effectively retain the intrinsic framework architecture of ZIF‐8.

The classification of the resulting cluster‐defects as either smaller or larger is contingent upon the domain distribution originating from the ligand ratio during synthesis. The formation of these cluster‐defects fundamentally influences the mesoporous structure, and their classification is intricately linked to the initial distribution of the thermolabile linker. This mesopore transition is characterized by its continuous nature, signifying that the mesopore structure of LR‐ZIFs undergoes a gradual evolution with escalating ligand ratios. This stands in contrast to the discrete dual‐pore features, comprising channels and cavities, observed in MIL‐125‐Ti, which necessitates distinct synthetic pathways.^[^ ^13^ ^]^

### 2.5 Enhancing Dye Adsorption in Water Purification

In order to assess the enhanced efficacy of the LR‐ZIFs, we conducted tests to evaluate the samples' ability to adsorb significant organic pollutants from water. Our previous work showcased the substantial improvement in glyphosate molecule adsorption by incorporating larger mesopores into MIL‐125‐Ti.^[^ ^40^ ^]^ This enhancement was attributed to the facilitated diffusion of glyphosate molecules into the MOF framework and the provision of new adsorption sites following ligand removal. These sites were ideally suited for glyphosate adsorption through phosphonate coordination.

**Figure 5:**
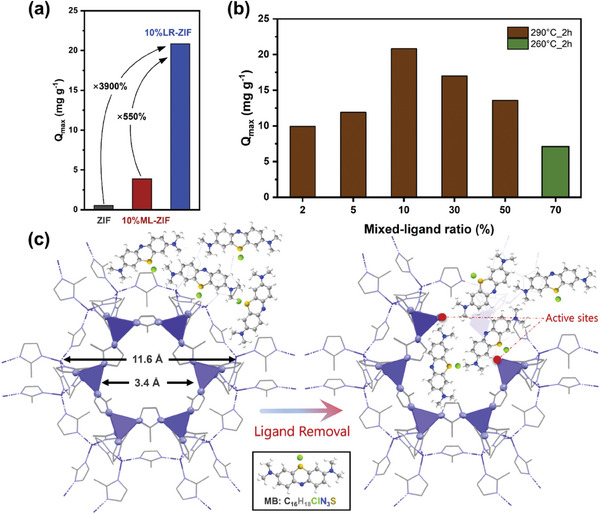
Adsorption of Methylene blue. Maximum adsorption capacity (*Q*_max_) of ZIF‐8, 10%NH_2_‐ZIF and 10%NH_2_‐ZIF‐2 h, revealing that the introduction of mesopores greatly enhances the adsorption of MB a). *Q*_max_ of LR‐ZIFs with different mixed‐ligand ratio b). Adsorption mechanism in unit cell of ZIF‐8 and LR‐ZIF; the MB molecule approximately has the dimensions 17.0 × 7.6 × 3.3 Å and fits well within the newly created mesoscale cavities c).

In this study, we examined how the introduction of hierarchical pores in ZIF‐8 impacts the adsorption of various dyes, including methylene blue (MB), methyl orange (MO), and Rhodamine B. The results are presented in Figures S35–S38 (see Section S6, Supporting Information).^[^ ^41^ ^]^ As summarized in **Figure** **5**a, the selective removal of ligands leads to a significant increase in the adsorption capacity for MB. Notably, the 10%NH_2_‐ZIF‐2 h sample exhibits the highest MB adsorption capacity, achieving an enhancement of ≈40 times compared to ZIF‐8 (Figure 5a,b).

The inherent pore channels of ZIF‐8 and ML‐ZIFs (with dimensions of 3.4 Å)^[^ ^42^ ^]^ appear to hinder the diffusion of MB molecules (with dimensions of 17.0 × 7.6 × 3.3 Å)^[^ ^43^ ^]^ into the central cavities with dimensions of 11.6 Å (see NLDFT Section 2.3 and),^[^ ^42^ ^]^ thus limiting the utilization of Zn centers as adsorption sites. In contrast, the creation of hierarchical micro/mesopores through ligand thermolysis eliminates this diffusion constraint, resulting in an amplified adsorption capacity (Figure 5c).

Similar trends are observed with the anionic methyl orange and the cationic rhodamine B (Figure S37a,b, Supporting Information), indicating that electrostatic interactions have a minor role in dye adsorption. Interestingly, the adsorption capacity of 10%ML‐ZIF is also enhanced by ≈5 times compared to pure ZIF‐8. While this enhancement is usually attributed to additional electrostatic adsorption between the sulfonic groups of MB and the ‐NH_2_ group on the surface,^[^ ^25^, ^44^ ^]^ the absence of significant electrostatic interactions in LR‐ZIFs point to a different mechanism. This suggests that diffusion plays a more crucial role than attractive electrostatic interactions.

The adsorption kinetics and isotherms data exhibit the closest agreement with the Pseudo‐second‐order (PSO) and Langmuir models, as summarized in Tables S4 and S5 (Supporting Information). This observation suggests that the adsorption reactions likely involve chemisorption and monolayer molecular adsorption, in line with previous research.^[^ ^45^, ^46^ ^]^ However, it is important to highlight that although the PSO model provides the best fit, this does not necessitate chemisorption within the ZIFs.^[^ ^47^ ^]^ It is essential to integrate findings from other analytical techniques and thoroughly investigate the chemical nature of the adsorbent surface.

For a more precise understanding of the adsorption process, we performed a comparative analysis of IR bands and XRD patterns before and after the adsorption of MB/MO. The post‐adsorption spectra exhibit no emergence of new bands or significant shifts in the existing bands. However, the IR bands from the dye references are distinctly visible and unchanged (refer to Figure S38a,b, Supporting Information). This confirms that the predominant mode of dye adsorption by the LR‐ZIF samples, irrespective of the dye's charge (anionic or cationic), is physisorption. Furthermore, our evaluation of powder XRD data and cyclic tests conducted before and after MB adsorption demonstrates that LR‐ZIFs serve as effective and enduring adsorbents (refer to Figure S38c,d, Supporting Information).

Certainly, the enhanced adsorption of dyes can be attributed to a combination of key factors. Primarily, the increased availability of space, particularly the higher pore volume facilitated by the mesopore cavities, plays a pivotal role in achieving a larger adsorption capacity. This augmented pore volume offers additional active sites within the adsorbent material where dyes can be effectively accommodated.

Equally important is the improved accessibility of these pore cavities. When the pores are easily accessible, dyes can seamlessly diffuse into the adsorbent matrix, resulting in a heightened adsorption efficiency. This improved accessibility ensures that dyes can engage effectively with the active sites present within the adsorbent structure, thus facilitating a more efficient adsorption process. It's important to note that this mechanism differs from the glyphosate adsorption process observed in MIL‐125‐Ti, which involves phosphonate coordination with unsaturated Ti sites combined with hydrogen bond formation.^[^ ^40^ ^]^

In essence, the synergistic effect of increased pore volume and enhanced accessibility to pore cavities significantly contributes to the overall heightened dye adsorption observed within the material.

## 3 Conclusion

Zeolitic imidazolate frameworks (ZIFs) represent a class of microporous materials comprising metal ions interconnected by imidazolate linkers. Despite their demonstrated potential in numerous applications, the absence of mesoscale cavities in most ZIFs can hinder specific functionalities that demand rapid and unimpeded access to active adsorption sites. Such functionalities encompass catalysis, sensing, and the adsorption of substantial molecules in both air and aqueous environments.

To surmount this limitation, we have pioneered an innovative approach by fabricating mixed‐ligand ML‐ZIFs, utilizing varying proportions of NH_2_‐bIm (2‐aminobenzimidazole) and 2‐mIm (2‐methylimidazole). Through precisely controlled thermal treatments, we achieve the quantitative elimination of the thermolabile ligand NH_2_‐bIm, thus introducing uniform mesopores with consistent size and structure within the ZIF matrix.

Our comprehensive investigation, leveraging an array of ex situ and in situ techniques such as XRD, SEM, TEM, ATR‐IR, TGA, and ^1^H NMR, has elucidated the mechanism of ligand removal, unaffected by the ambient atmosphere (air, Ar, or N_2_). This indicates that the removal process ensues via the selective cleavage of metal‐ligand coordination, rather than oxidation. However, the close temperature proximity between NH_2_‐bIm and 2‐mIm presents a challenge in achieving precise ligand removal, necessitating meticulous calibration of heating temperature and duration for each mixed‐ligand composition. Such careful calibration is imperative to achieve the complete elimination of NH_2_‐bIm, the introduction of uniform mesopores, the prevention of ZnO nanoparticle formation, and the preservation of the inherent microporous framework of the ZIF.

Furthermore, our observations underscore the pivotal role played by the initial distribution of the thermolabile ligand in the as‐synthesized ML samples, influencing the resulting configuration of the mesopores. This emphasizes the critical importance of stringent control during the synthesis process.

To demonstrate the versatility and broad applicability of our selective ligand thermolysis, we have evaluated the resulting hierarchically porous ZIF specimens for the adsorption of organic dyes in aqueous media. Impressively, the adsorption capacity for methylene blue (MB) in the case of 10%NH_2_‐ZIF‐2 h exhibited an ≈40‐fold increase compared to conventional ZIF‐8. Moreover, the 10%NH_2_‐ZIF‐2 h sample showcased recyclability and stability in MB adsorption. This substantial enhancement in adsorption performance is attributed to the incorporation of additional mesopores through the Selective Ligand Removal (SeLiRe) process, which provides larger pore volumes for dye molecules and facilitates expedited transport of these molecules to adsorption sites.

In essence, our work introduces a promising avenue for designing hierarchically micro/mesoporous ZIFs with diverse topologies, thereby unveiling fresh possibilities for a range of applications.

## 4 Experimental Section

### Chemicals

Zinc nitrate hexahydrate (Zn(NO_3_)_2_·6H_2_O, 98%, ACROS), Cobalt nitrate hexahydrate (Co(NO_3_)_2_·6H_2_O, 99%, STREM) 2‐methylimidazole (2‐mIm, C_4_H_6_N_2_, 99%, Sigma–Aldrich), 2‐aminobenzimidazole (NH_2_‐bIm, C_7_H_7_N_3_, 97%, Sigma–Aldrich), Methanol (MeOH, 99.9%, HiPerSolv CHROMANORM, VWR), Potassium bromide for IR spectroscopy (99%, Fisher scientific, Austria), Sulfuric acid (H_2_SO_4_, 99.9%, Sigma–Aldrich), Methylene blue (MB, C_16_H_18_CIN_3_S·xH_2_O, high purity, biological stain, Alfa Aesar), Methyl orange (MO, C_14_H_14_N_3_NaO_3_S, 90%, Sigma–Aldrich), Rhodamine B (RhB, C_28_H_31_ClN_2_O_3_, pure, 99%, ACROS), d_4_‐acetic acid for NMR (CD_3_CO_2_D, 99.4%, Thermo scientific).

### Ligand‐Removed ZIF‐8

LR‐ZIF‐8 or R%NH_2_‐ZIF‐time/temp refers to the ZIF‐8 samples obtained by thermolysis of ML‐ZIF‐8 at specific temperatures for specific time periods (Section S7, Supporting Information). The heating process was conducted using a muffle furnace (LT 5/12 Nabertherm, Germany) in an air atmosphere, ramp rate of 10 °C min^−1^. Or a tube furnace (HTM Reetz LK‐1100, Germany) with argon/nitrogen flow at a ramp rate of 10 °C min^−1^. The final temperature was allowed to cool naturally to room temperature. For air atmosphere, the calcination temperature is 290 °C for 0–50%NH_2_‐ZIFs and 260 °C for 70%. For argon/nitrogen flow, the calcination temperature was 500 °C for 30%NH_2_‐ZIF and 400 °C for 70%NH_2_‐ZIF. The samples were labeled as R%NH_2_‐ZIF‐time/temp (or R%‐time/temp), where R% indicates the mixing ratio of various NH_2_‐bIm, time is the calcination time, and temp indicates the special calcination temperature.

### Characterization

Powder XRD measurements were performed using PANalytical X'Pert Pro multi‐purpose diffractometer (MPD) with Bragg Brentano geometry with Cu anode at 45 kV, 40 mA, equipped with a BBHD Mirror and an X‐Celerator multichannel detector. In situ XRD is used with the addition of the Anton Paar HTK 1200 temperature control system, with a heating rate of 5 °C min^−1^. The sample was heated with a flow rate of 0.5 mL min^−1^ of air, argon, and nitrogen. ATR‐IR spectra were obtained with Perkinelmer Spectrum two FT‐IR spectrometer, with the LiTaO_3_ (lithium tantalate) MIR detector. N_2_ Physisorption measurements were conducted at a temperature of 77 K, on a 3Flex instrument by Micromeritics. SEM images were recorded on a FEI Quanta 250 (Schottky‐)FEG‐SEM, TEM measurements displayed in this paper were performed on a Tecnai F20 FEG‐TEM, facilitated by USTEM (university service center for transmission electron microscopy) at TU Wien. Raman spectroscopy measurements were carried out using the WITec alpha 300 RSA. UV–Vis and DRS were obtained at 660–670 nm by Jasco V‐670. Liquid phase ^1^H NMR spectra were measured using the Bruker ADVANCE 250 (250.13 MHz) instrument, which is equipped with a 5 mm inverse‐broad probe head and z‐gradient unit. TGA measurements were carried out using PerkinElmer 8000 (Waltham, USA) with the sample heated in of Al_2_O_3_ crucible DFT calculations were performed by using the Vienna ab initio program package (VASP). More details are in the Supporting Information.

## Conflict of Interest

The authors declare no conflict of interest.

## Author Contributions

Z.H. carried out most of the experiments and analysis and wrote the initial manuscript. J.R. conducted SEM and TEM, Q.Z. performed DFT simulations. A.C. reviewed and edited the manuscript. S.N. conducted N_2_ physisorption and ^1^H NMR, reviewed and edited the manuscript. D.E. outlined and supervised the project, reviewed and edited the final manuscript.

## Supporting information

Supporting Information

Supporting Information

## Data Availability

The data that support the findings of this study are available in the supplementary material of this article.
