# Supplementary material for: Hierarchically Micro‐ and Mesoporous Zeolitic Imidazolate Frameworks Through Selective Ligand Removal
Source: Small. 2023 Dec 21;20(21):2307981. doi: 10.1002/smll.202307981 (PMC11478943; doi:10.1002/smll.202307981)
Supplement: Supplementary file 1 — Supporting Information [file SMLL-20-2307981-s001.pdf]

NANO · MICRO
small

Supporting Information

for *Small*, DOI 10.1002/smll.202307981

Hierarchically Micro- and Mesoporous Zeolitic Imidazolate Frameworks Through Selective Ligand Removal

*Zheao Huang, Jakob Rath, Qiancheng Zhou, Alexey Cherevan, Shaghayegh Naghdi and Dominik Eder**

Supporting Information

Hierarchically Micro- and Mesoporous Zeolitic Imidazolate Frameworks through Selective Ligand Removal

Zheao Huang[1], Jakob Rath[1], Qiancheng Zhou[2], Alexey Cherevan[1], Shaghayegh Naghdi[1], and Dominik Eder[,1]*

[1] Institute of Material Chemistry, Vienna University of Technology, 1060, Vienna, Austria

[2] Institute of Nanoscience and Nanotechnology, College of Physical Science and Technology, Central China Normal University, 430079, Wuhan, China

* Corresponding author. Email: dominik.eder@tuwien.ac.at.

Contect

S1. Ligand NH_2-bIm mixing and removal process.. 3

S2. Ligand mixing and removal process for ZIF-67 19

S3. Characterization of pore structures 23

S4. Ligand removal in inert gases 29

S5. DFT simulation models... 40

S6. Enhancing Dyes Adsorption 43

S7. Materials Synthesis and Experimental Section 49

S8. References... 55

S1. Ligand NH_2-bIm mixing and removal process

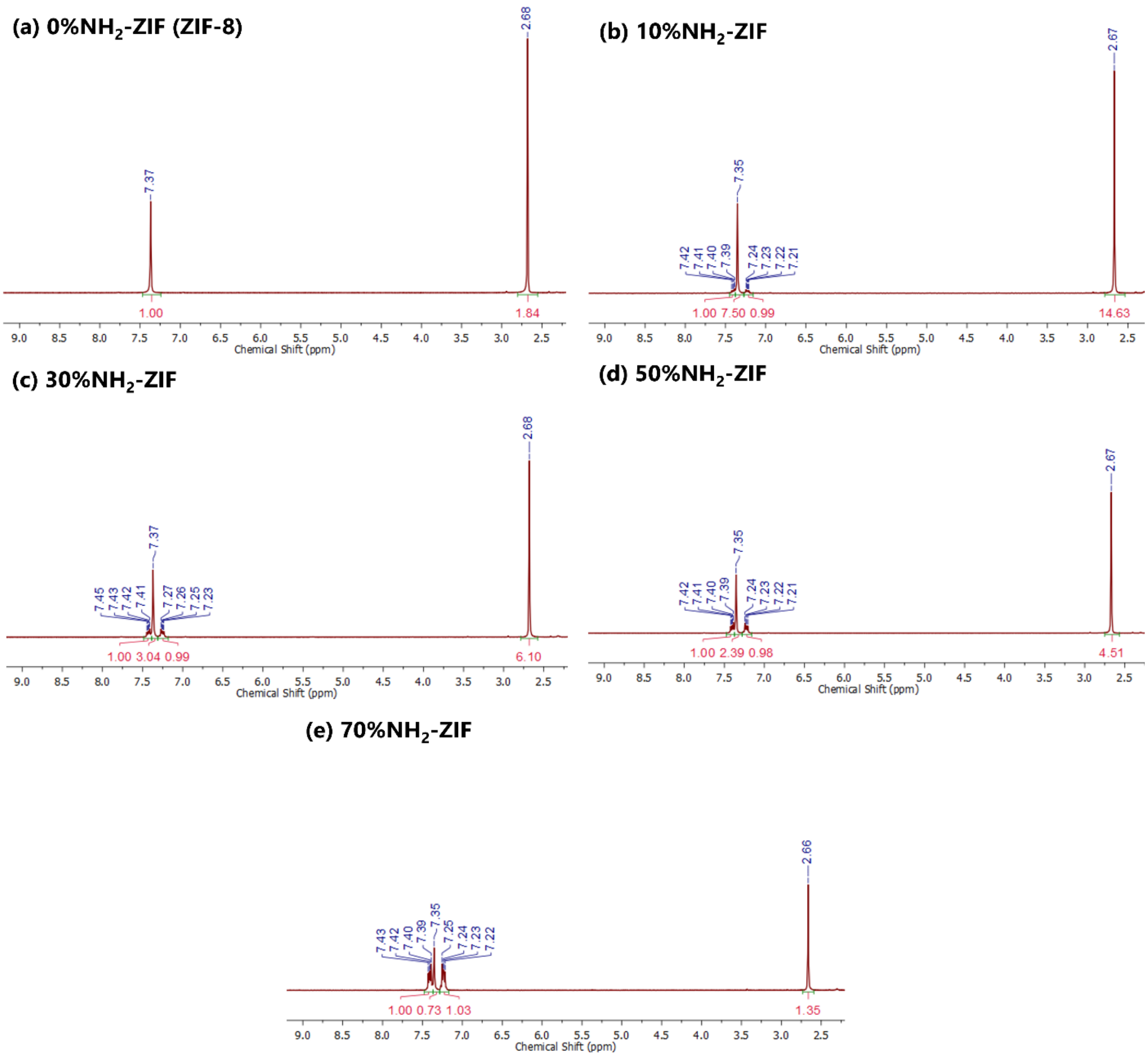

Figure S1. ^{1}H NMR spectroscopy of 0-70%NH_2-ZIFs (a, 0%NH_2-ZIF; b, 10%NH_2-ZIF; c, 30%NH_2-ZIF; d, 50%NH_2-ZIF; e, 70%NH_2-ZIF). The actual ratios of NH_2-bIm can be obtained in **Table S1**.

0%NH_2-ZIF: *^{1}H NMR (250 MHz, Acetic Acid-d_4) δ 2.55 – 2.80 (s, 2H), 7.24 – 7.47 (s, 1H).* 10%NH_2-ZIF: *^{1}H NMR (250 MHz, Acetic Acid-d_4) δ 2.54 – 2.79 (s, 15H), 7.16 – 7.27 (dd, J = 3.2, 6.0 Hz, 1H), 7.27 – 7.38 (s, 7H), 7.38 – 7.45 (dd, J = 3.2, 6.0 Hz, 1H).* 30%NH_2-ZIF: *^{1}H NMR (250 MHz, Acetic Acid-d_4) δ 2.57 – 2.79 (s, 6H), 7.18 – 7.31 (dd, J = 3.2, 6.0 Hz, 1H), 7.31 – 7.39 (s, 3H), 7.39 – 7.48 (dd, J = 3.2, 5.9 Hz, 1H).* 50%NH_2-ZIF: *^{1}H NMR (250 MHz, Acetic Acid-d_4) δ 2.57 – 2.75 (s, 5H), 7.16 – 7.28 (dd, J = 3.2, 6.0 Hz, 1H), 7.28 – 7.37 (s, 2H), 7.37 – 7.47 (dd, J = 3.2, 6.0 Hz, 1H).* 70%NH_2-ZIF: *^{1}H NMR (250 MHz, Acetic Acid-d_4) δ 2.59 – 2.73 (s, 1H), 7.17 – 7.28 (dd, J = 3.2, 6.0 Hz, 1H), 7.28 – 7.37 (s, 1H), 7.37 – 7.48 (dd, J = 3.2, 5.9 Hz, 1H).*

Table S1. Calculation of the NH_2-bIm ratio in ML-ZIFs by ^{1}H NMR.

**Samples**	**10%NH$_2$-ZIF**	**30%NH$_2$-ZIF**	**50%NH$_2$-ZIF**	**70%NH$_2$-ZIF**
**Synthetic mass of NH$_2$-bIM/2-mIm (g/g)**	0.2/1.8	0.6/1.4	1/1	1.4/0.6
**Synthetic ratio of NH$_2$-bIm (mol%)**	6.4%	20.9%	38.2%	60.0%
**Actual ratio of NH$_2$-bIm (mol%)**	8.2%	17.9%	22.3%	49.4%

Due to the competitive coordination between the two ligands, the actual ratio gradually deviates from the synthetic ratio as the ratio of NH_2-bIm increases. In thermodynamics, the Zn metal center has a higher affinity for 2-mIm than NH_2-bIm [1, 2]. Moreover, the NH_2-bIm possesses the redundant benzene ring and is excessively bloated for the formation of new Zn-N_β bonds.

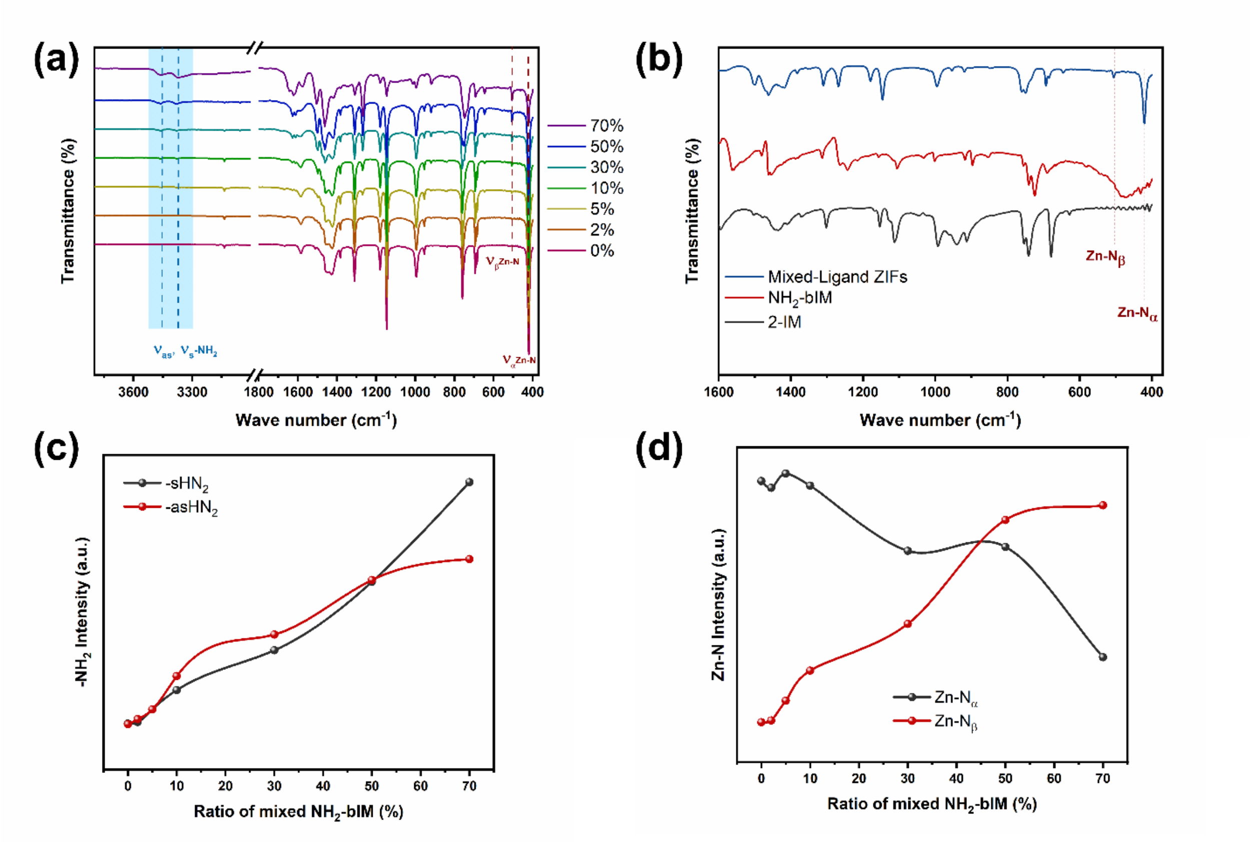

Figure S2. ATR-IR spectra of 0-70%NH_2-ZIFs (a). ATR-IR spectra of 2-mIm, NH_2-bIm and 50%NH_2-ZIF (b). Intensity plot of ν_s(-NH_2) intensity (c) and ν(Zn-N) intensity (d) with the mixed-ligand ratio.

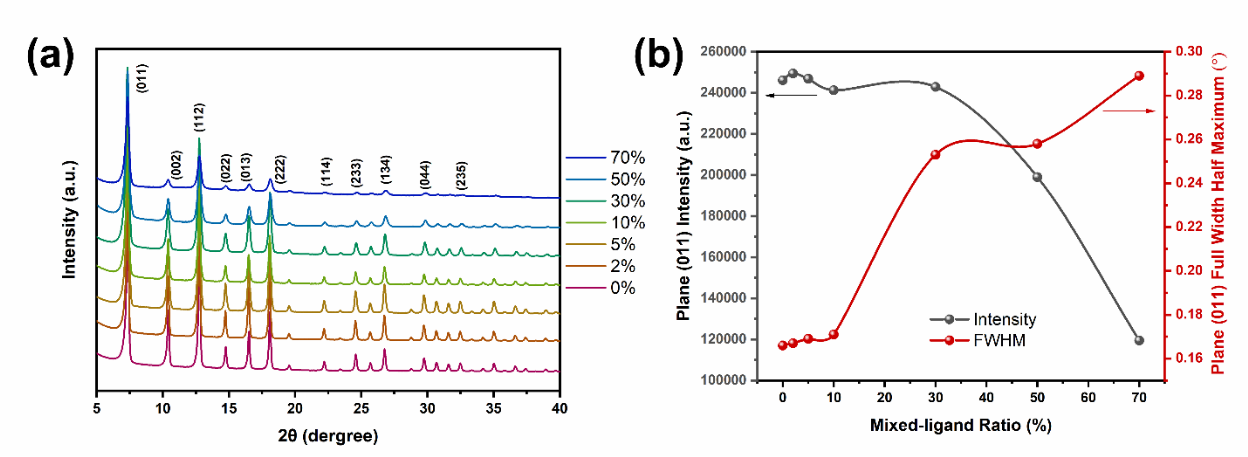

Figure S3. XRD patterns of 0-70%NH_2-ZIFs (a). Evolution plots of the (011) intensity and FWHM with the mixed-ligand ratio (b). XRD patterns of the NH_2-ZIFs are essentially indistinguishable from the simulated ZIF-8. As the ratio of NH_2-bIm increases, the low-angle peaks, particularly the unit lattice constants of (011) changed, show changes in the form of reduced crystallinity and increased FWHM.

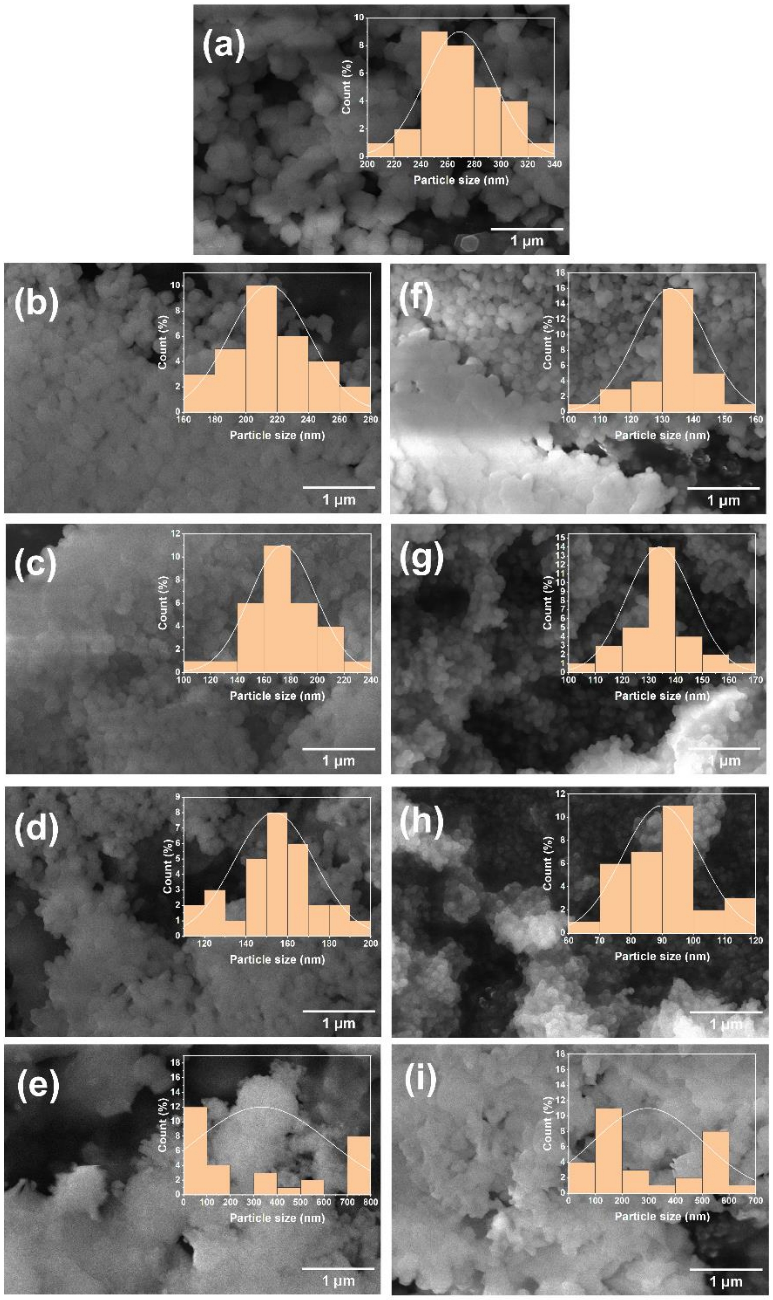

Figure S4. SEM images of 0-70%NH_2-ZIFs (a, 0%NH_2-ZIF; b, 10%NH_2-ZIF; c, 30%NH_2-ZIF; d, 50%NH_2-ZIF; e, 70%NH_2-ZIF) and 10-70%NH_2-ZIF-2h (f, 10%NH_2-ZIF-2h; g, 30%NH_2-ZIF-2h; h, 50%NH_2-ZIF-2h; i, 70%NH_2-ZIF-2h). Noted that the particle sizes of LR-ZIFs were slightly reduced due to oxidation reactions. An ImageJ program was used to select 30 particles from each sample.

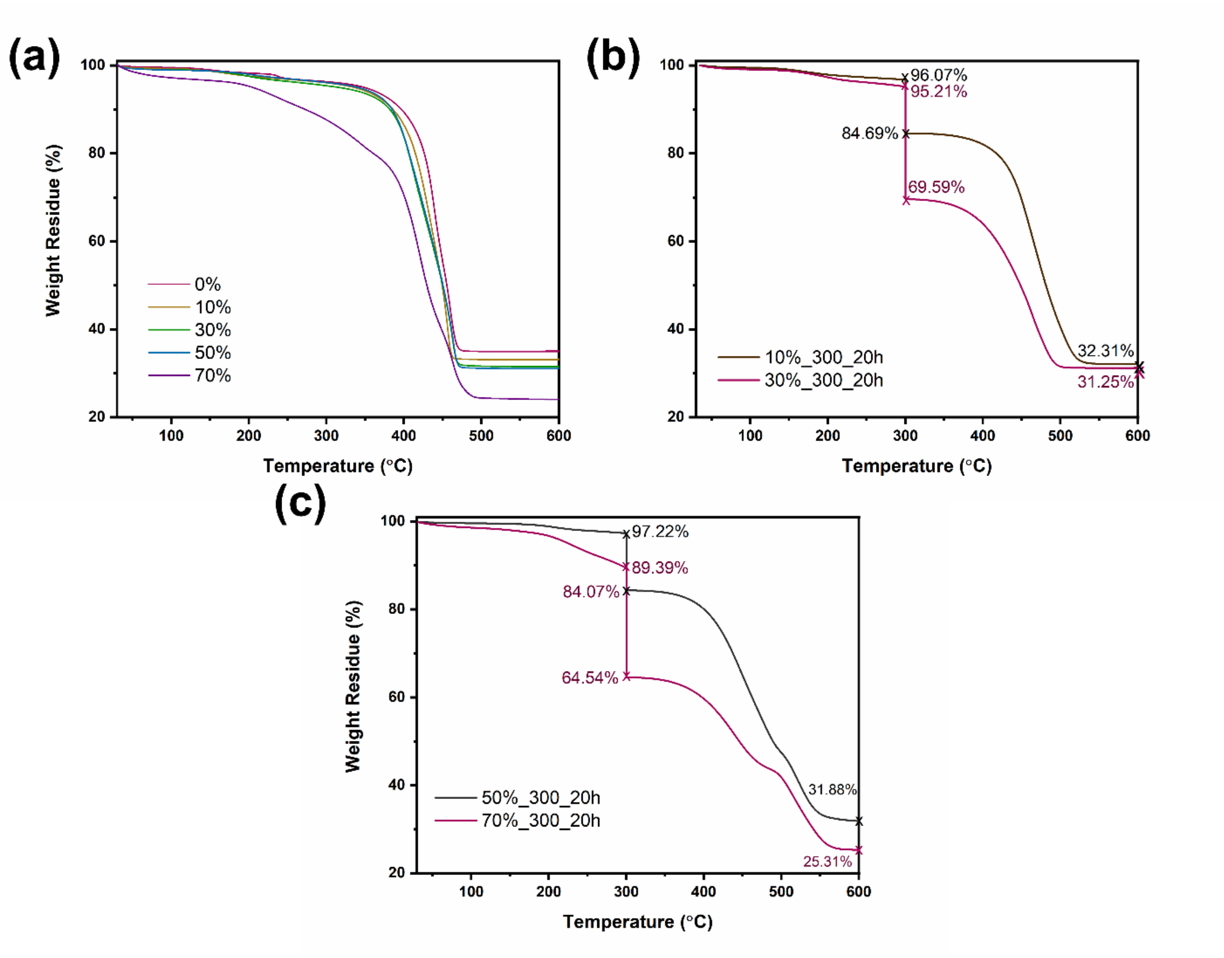

Figure S5. TGA curves of 0-70%NH_2-ZIFs (a). Isothermally heated TGA curves of 10/30%NH_2-ZIF (b) and 50/70%NH_2-ZIF (c) that hold continuously at 300°C for 20 hours. The sudden weight loss is attributed to the prolonged isothermal TGA testing, a departure from the typical TGA setup where the temperature and time are continuously increasing. All TGA tests were all carried out in air atmosphere.

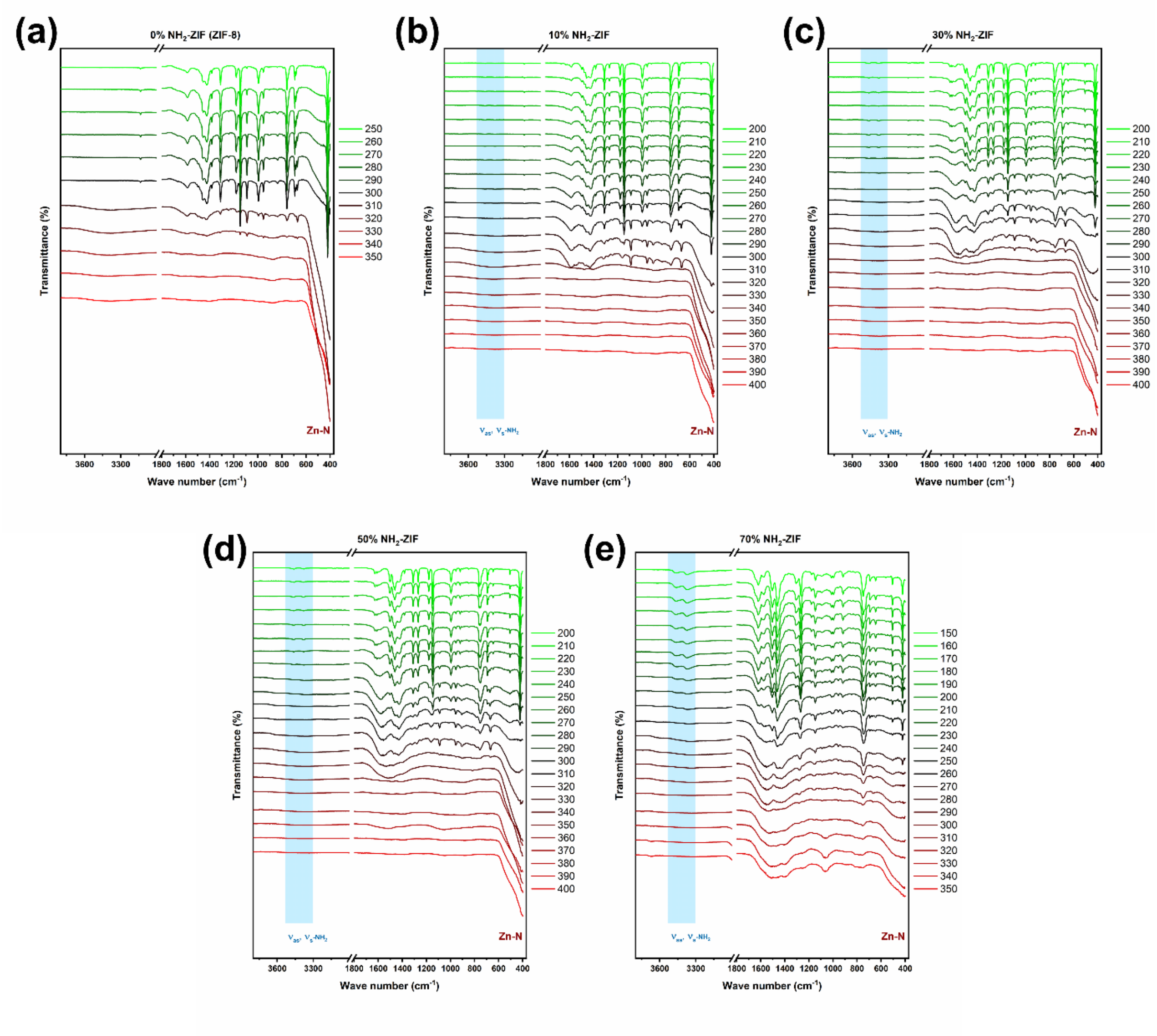

Figure S6. Ex-situ ATR-IR spectra of 0-70%NH_2-ZIFs (a, 0%NH_2-ZIF; b, 10%NH_2-ZIF; c, 30%NH_2-ZIF; d, 50%NH_2-ZIF; e, 70%NH_2-ZIF) at different temperatures. All ML-ZIFs were calcined in a muffle furnace, in air atmosphere, for a period of 2 hours and then cooled naturally to room temperature.

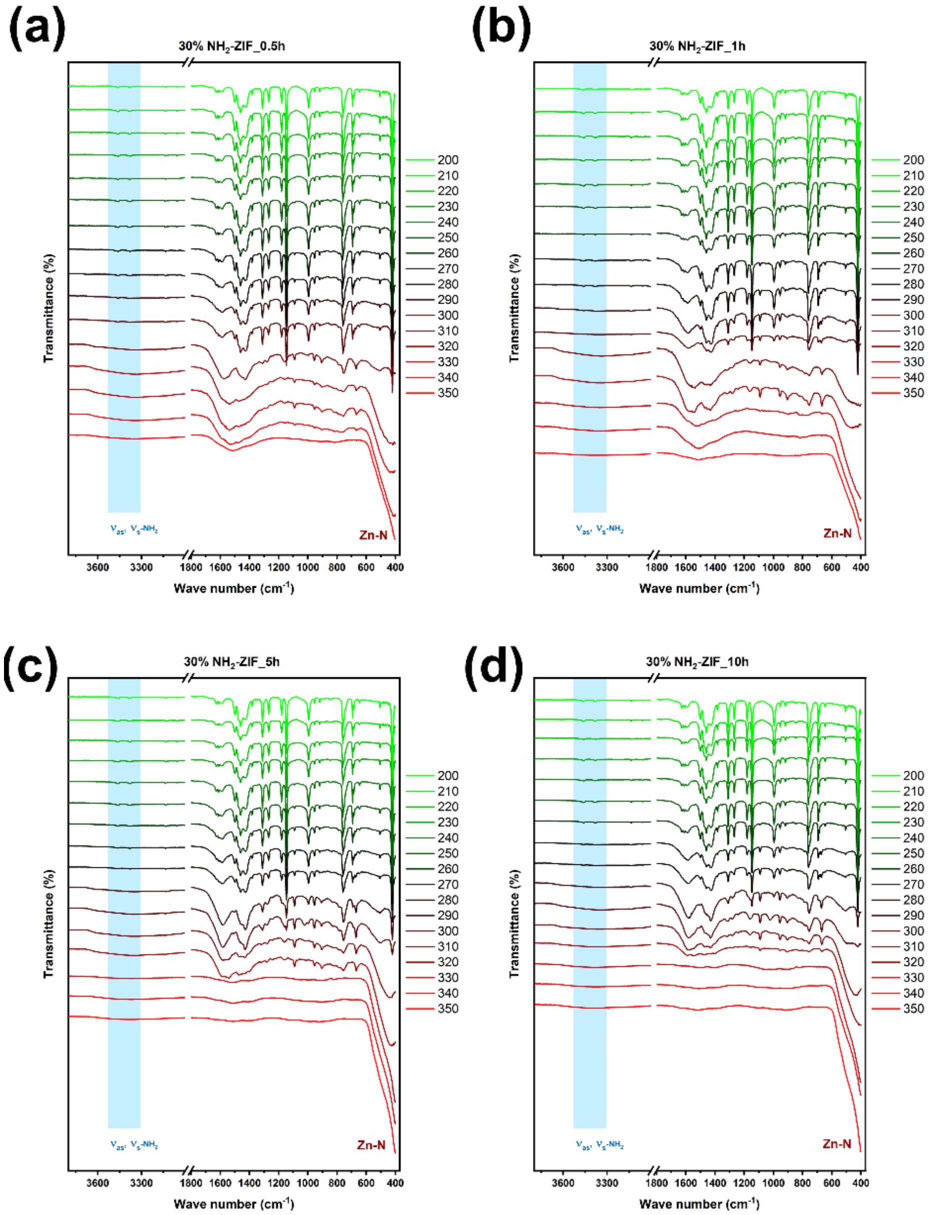

Figure S7. Ex-situ ATR-IR spectra of 30%NH_2-ZIFs at different calcination times (a, 0.5 hours; b, 1 hour; c, 5 hours; d, 10 hours). 30%NH_2-ZIFs were calcined in a muffle furnace in air atmosphere at 290°C. The data for calcination for 2 hours is included in **Figure S6c**.

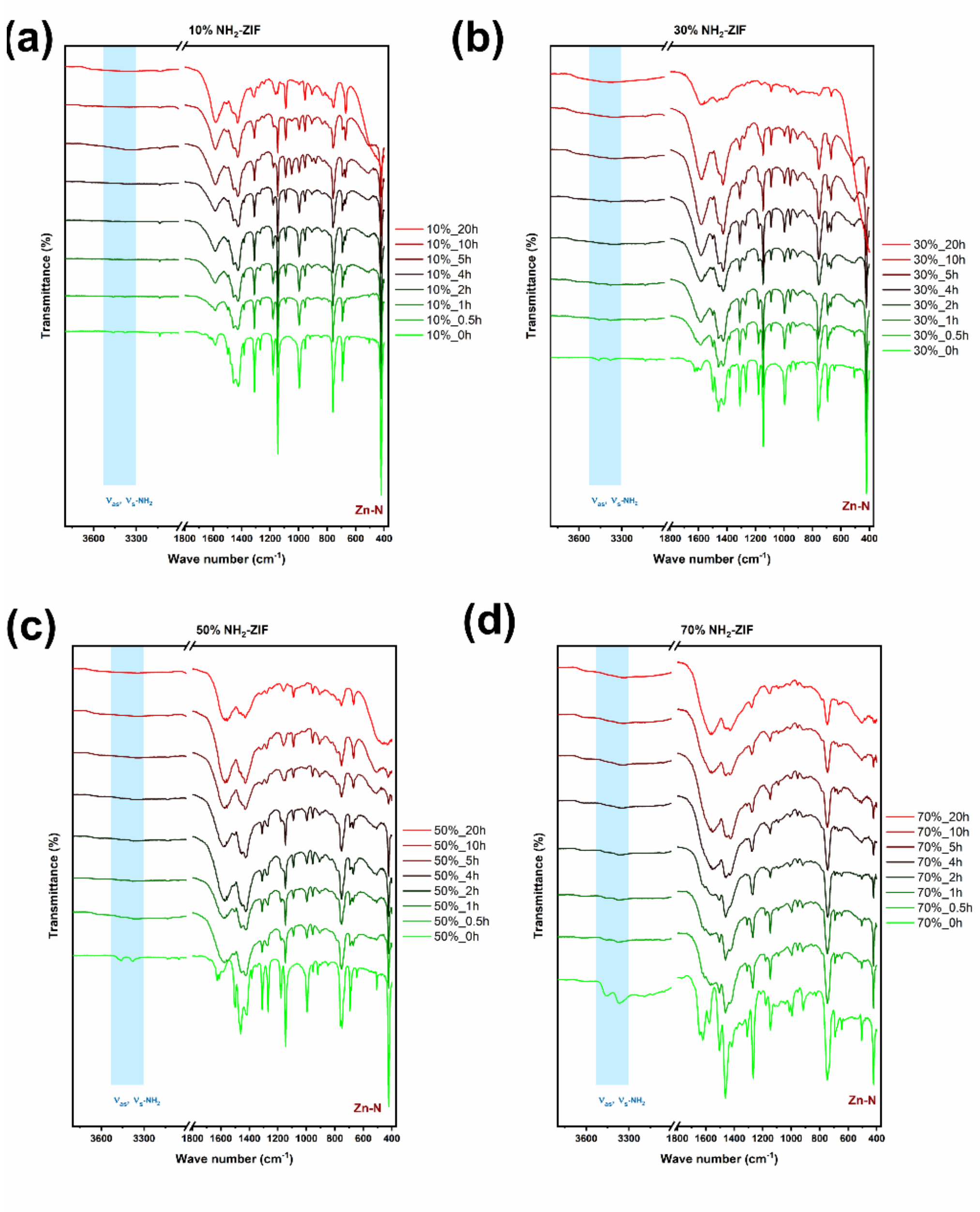

Figure S8. Isothermal ex-situ ATR-IR spectra of 10-70%NH_2-ZIFs (a, 10% NH_2-ZIF; b, 30% NH_2-ZIF; c, 50%NH_2-ZIF; d, 70%NH_2-ZIF) at different calcination times. All NH_2-ZIFs were calcined in a muffle furnace in the air atmosphere. 10-50% at 290°C and 70% at 260°C.

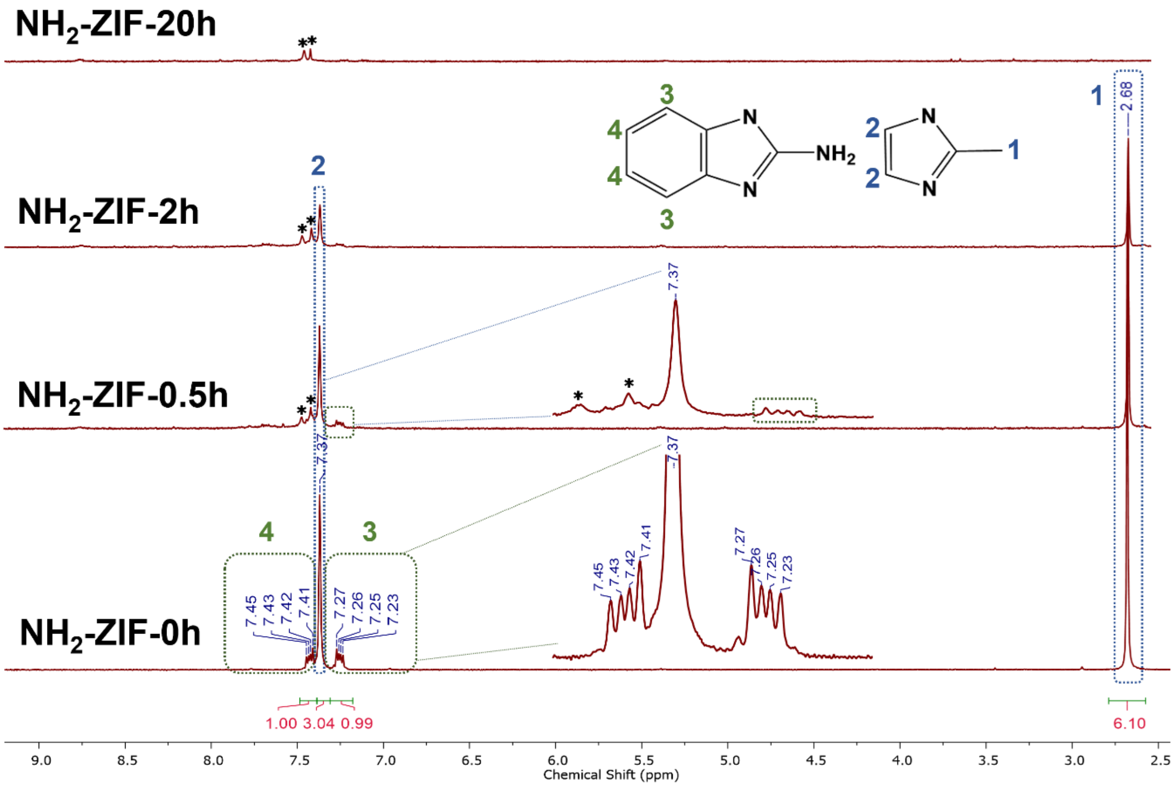

Figure S9. ^{1}H NMR spectroscopy of LR-ZIFs at different calcination times (30%NH_2-ZIF-0h; 30%NH_2-ZIF-0.5h; 30%NH_2-ZIF-2h; 30%NH_2-ZIF-20h). Before heating, the NMR of 30%NH_2-ZIF-0h (ML-ZIF) exhibits typical spectrum features of both 2-mIm and NH_2-bIM. After heating, the characteristic quartets of NH_2-bIm on both sides of 7.35 ppm was markedly reduce and their chemical shifts were barely visible after 2 hours, confirming that the NH_2-bIm can be completely removed by ligand thermolysis strategy. *By-products such as azirine or benzimidazole resulting from the oxidation of the -NH_2 group or ligand NH_2-bIm [3-5], are supported by the cleavage of $\nu_{s,\,as}$(-NH_2) in ATR-IR (**Figures 2a** and **2b**). After 20 hours, the two ligands were removed and only the *by-products remained, corresponding to the results of isothermal XRD and ATR-IR (**Figures S6-9**, **S13** and **S14**).

30%NH_2-ZIF-0h: *^{1}H NMR (250 MHz, Acetic Acid-d_4) δ 2.57 – 2.79 (s, 6H), 7.18 – 7.31 (dd, J = 3.2, 6.0 Hz, 1H), 7.31 – 7.39 (s, 3H), 7.39 – 7.48 (dd, J = 3.2, 5.9 Hz, 1H).* 30%NH_2-ZIF-0.5h: *^{1}H NMR (250 MHz, Acetic Acid-d_4) δ 2.59 – 2.76 (s, 3H), 7.17 – 7.30 (m, 0H), 7.31 – 7.40 (s, 2H), 7.40 – 7.53 (d, J = 13.5 Hz, 1H).* 30%NH_2-ZIF-2h: *^{1}H NMR (250 MHz, Acetic Acid-d_4) δ 2.62 – 2.74 (s, 2H), 7.21 – 7.29 (s, 0H), 7.32 – 7.40 (s, 1H), 7.40 – 7.52 (d, J = 13.6 Hz, 1H).* 30%NH_2-ZIF-20h:*^{1}H NMR (250 MHz, Acetic Acid-d_4) δ 7.24 – 7.44 (d, J = 9.0 Hz, 1H).*

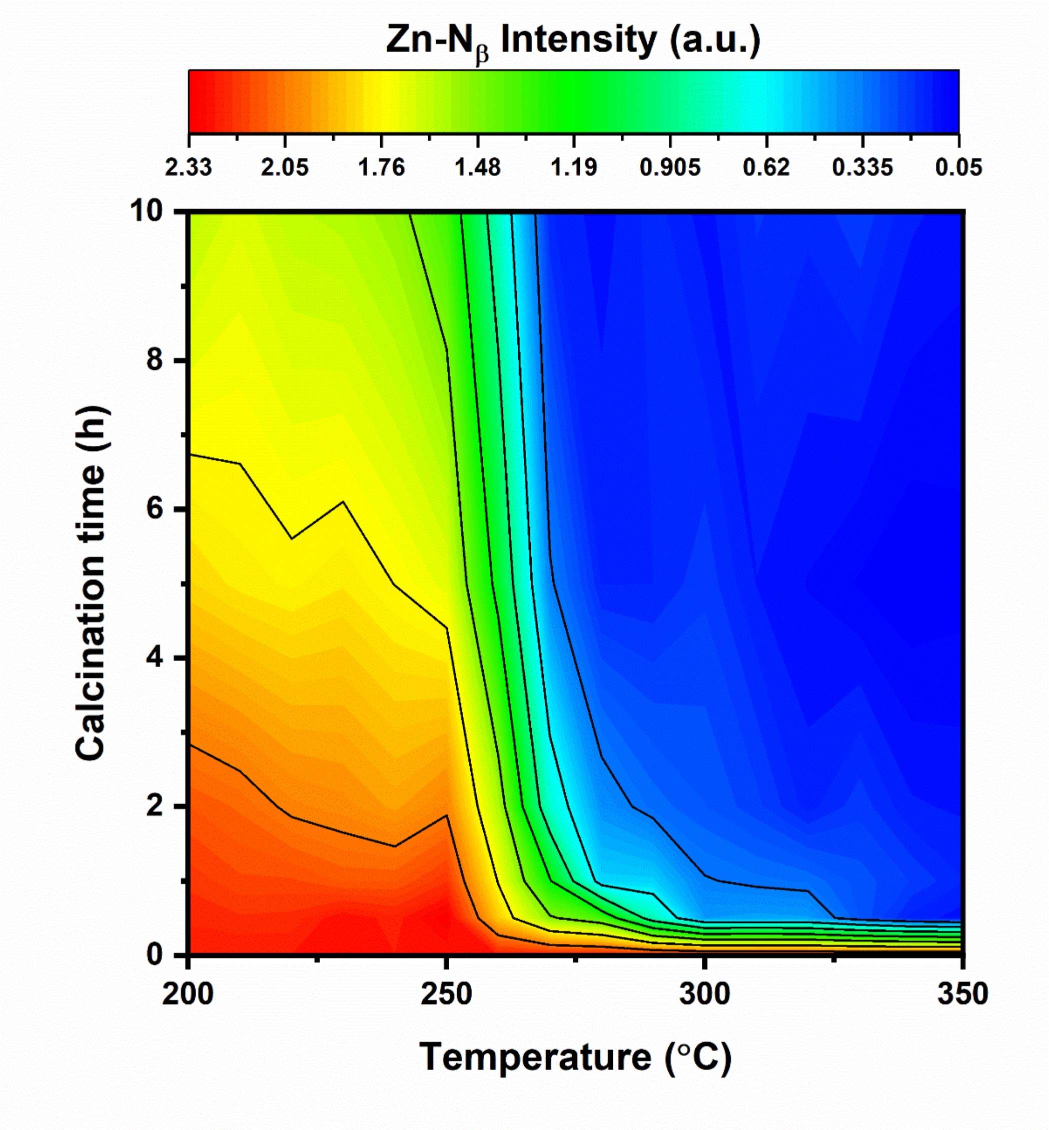

Figure S10. 2D contour plot of ν(Zn-N$_β$) intensity in 30%NH_2-ZIFs vs. mixed NH_2-bIm ratio with calcination temperature. Data are from the IR spectra in **Figures S6** and **S8**.

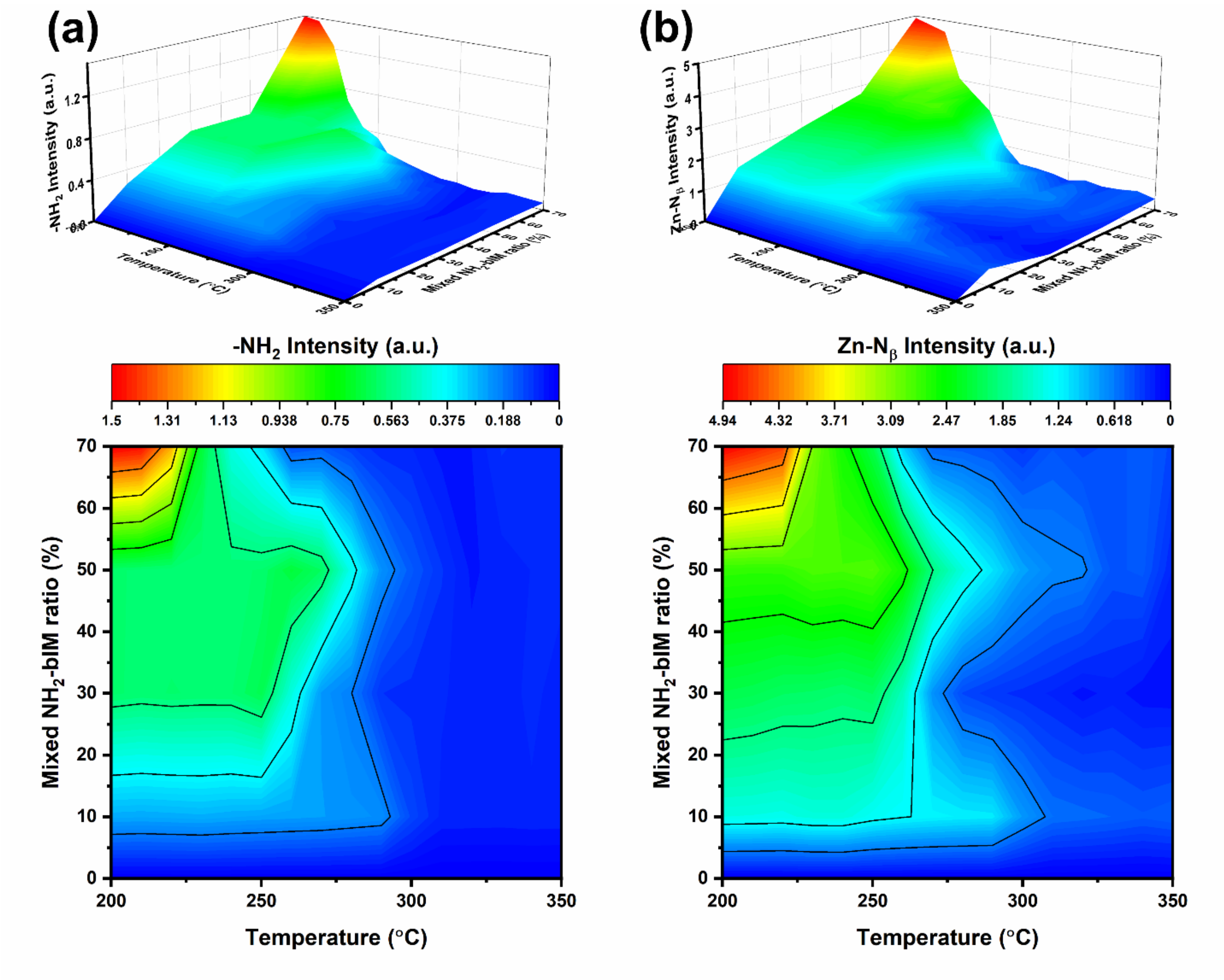

Figure S11. 2D/3D contour plot of ν(-NH_2) intensity (a) and ν(Zn-N_β) intensity (b) in ML-ZIFs vs. mixed NH_2-bIm ratio with calcination temperature. Data are from the IR spectra in **Figure S6**.

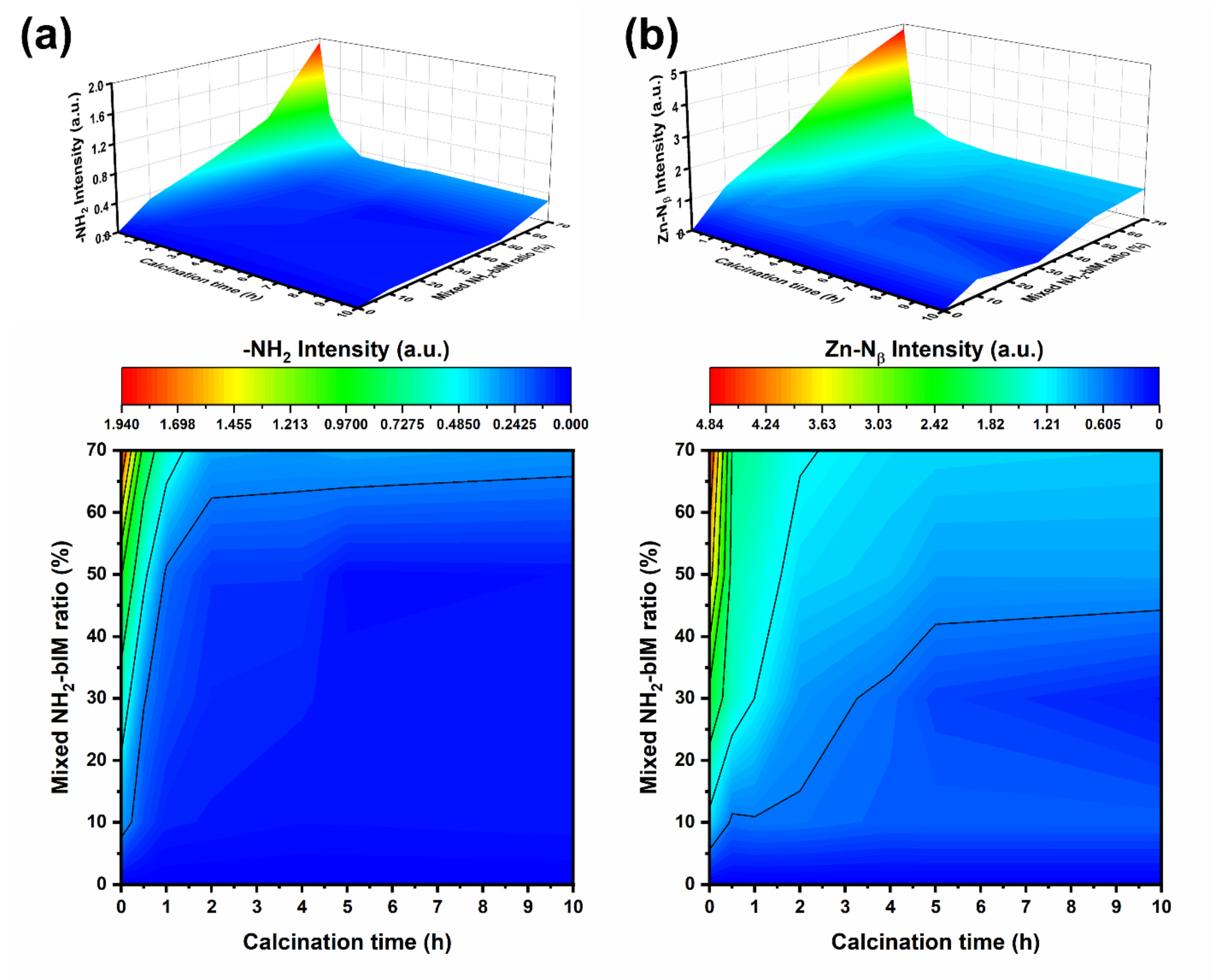

Figure S12. 2D/3D contour plot of ν($-NH_2$) intensity (a) and ν(Zn-N_β) intensity (b) in ML-ZIFs vs. mixed NH_2-bIm ratio with calcination time. Data are from the IR spectra in **Figure S8**.

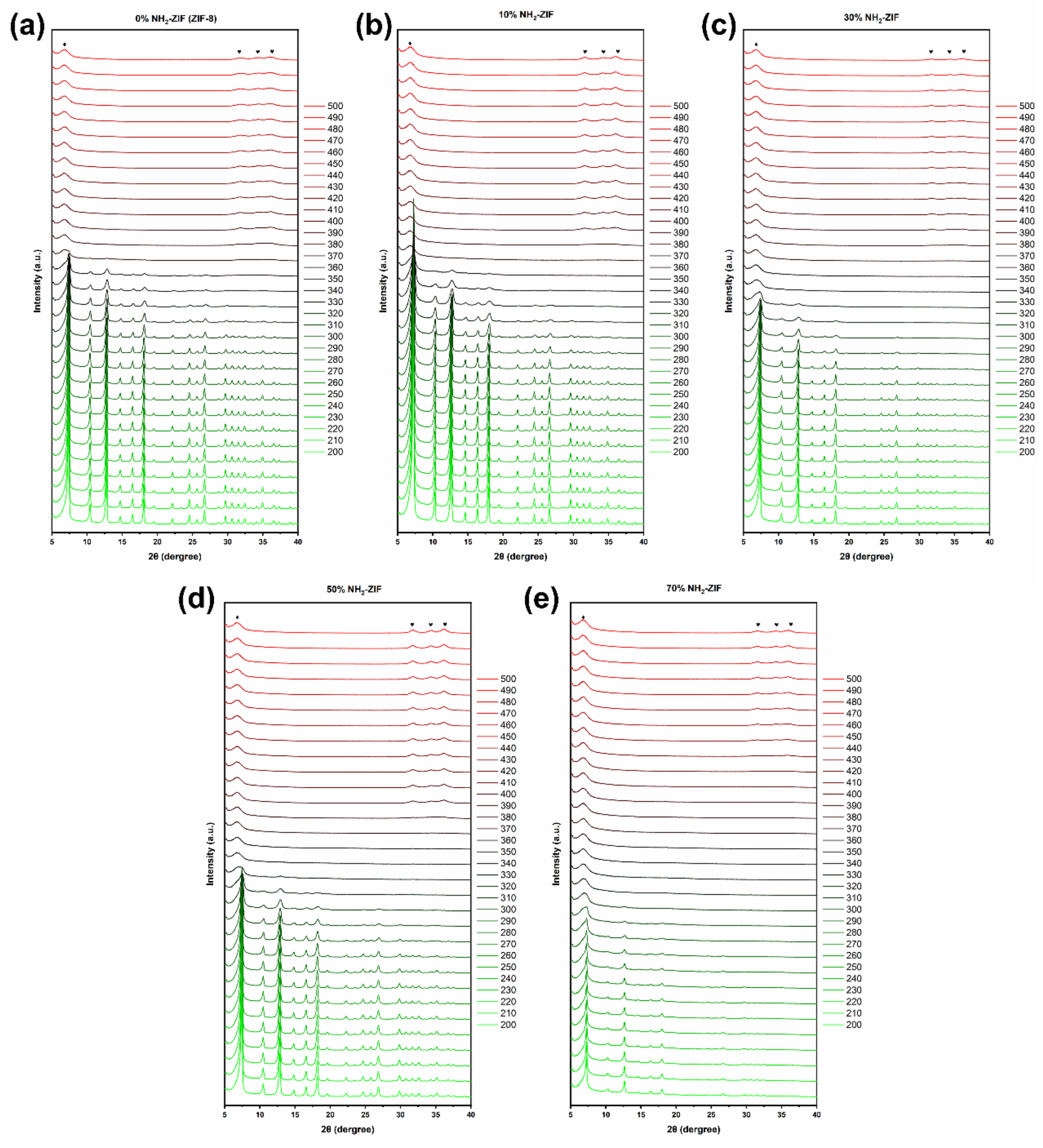

Figure S13. Temperature-programmed in-situ XRD patterns of 0-70%NH_2-ZIFs in air atmosphere (a, 0%NH_2-ZIF; b, 10%NH_2-ZIF; c, 30%NH_2-ZIF; d, 50%NH_2-ZIF; e, 70%NH_2-ZIF). The (011) peak, which is the first peak and has the strongest intensity, is a suitable choice for revealing and studying the transformation of the crystal structure during ligand thermolysis.

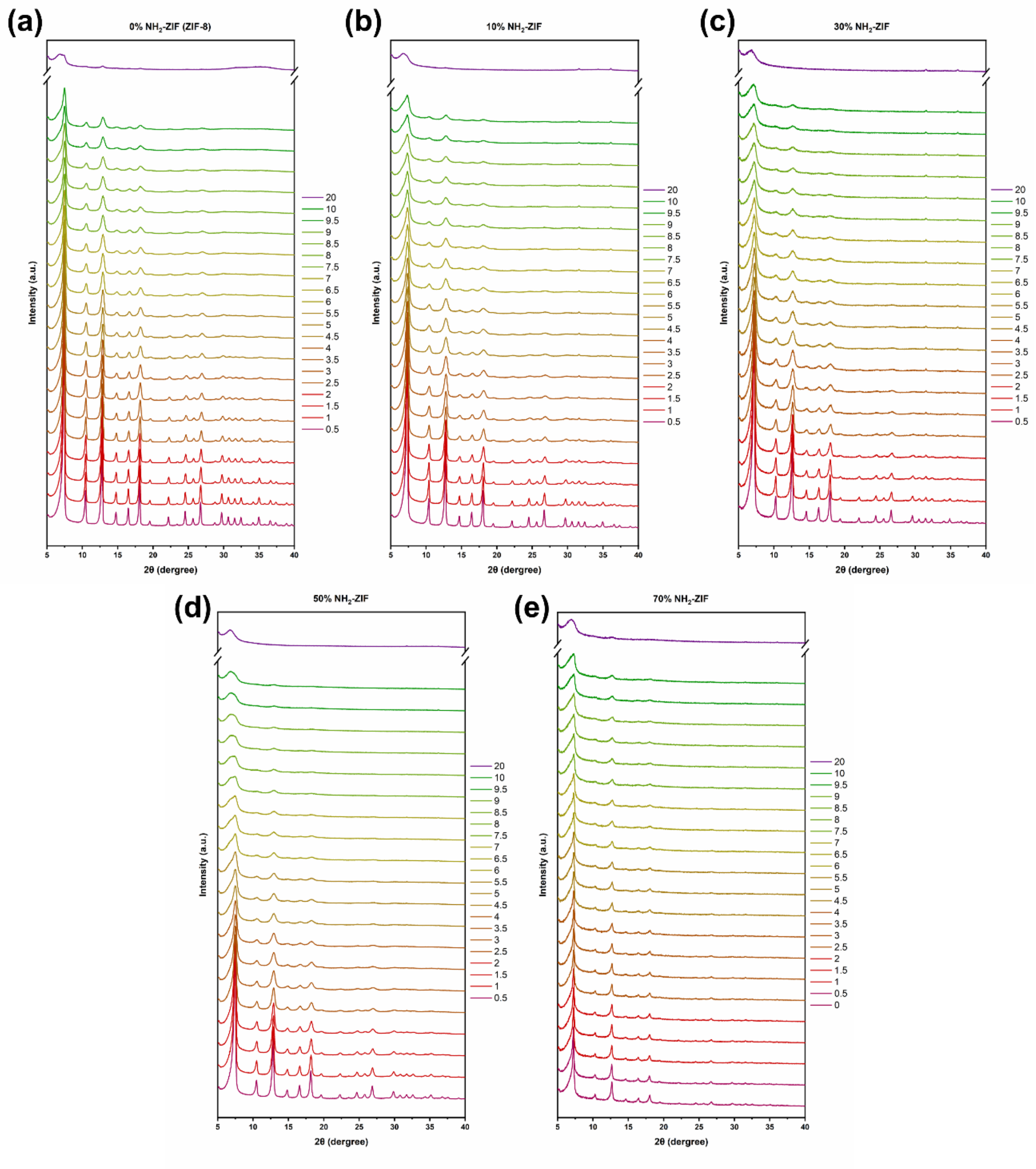

Figure S14. Isothermal in-situ XRD patterns of 0-70%NH2-ZIFs (a, 0%NH2-ZIF; b, 10%NH2-ZIF; c, 30%NH2-ZIF; d, 50%NH2-ZIF; e, 70%NH2-ZIF;) at different calcination times in air atmosphere. 0-50% at 290°C and 70% at 260°C.

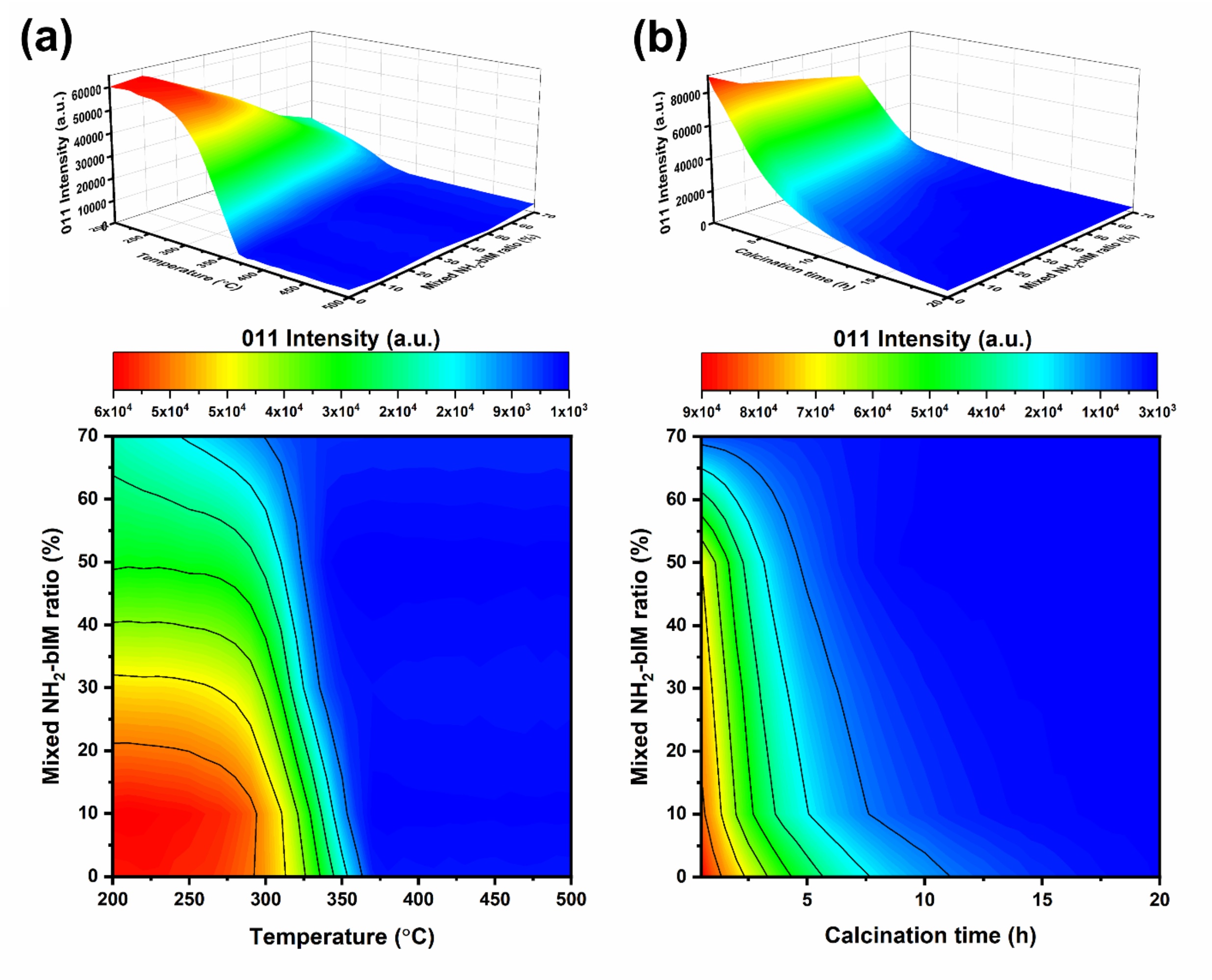

Figure S15. 2D/3D contour plot of (011) intensity in ML-ZIFs vs. mixed NH2-bIm ratio with temperature (a) and calcination time (b). Data are from the XRD patterns in **Figures S13** and **S14**.

S2. Ligand mixing and removal process for ZIF-67

Mixed-ligand ZIF-67 and its ligand thermolysis process. ZIF-67 and ZIF-8 share the same topology (SOD) in the family of ZIFs [6], with cobalt serving as the metal center. Since, we attempted to mix NH_2-bIminto ZIF-67 and successfully synthesized NH_2-ZIF67 (ML-ZIF-67), as confirmed by presence of ν(Co-$N_β$) and ν(-NH_2), as well as the XRD patterns (**Figures S16a** and **S16b**). Due to ZIF-67 and ZIF-8 are isostructural with the similar lattice parameters and the same organic ligands, the outcome is almost identical to that of the ML-ZIF-8. However, because coordination differences between Zn-N and Co-N, their thermal stability differs, with the unsaturated Co-N (outer shell is $3d^7 4s^2$) being more susceptible to oxidation than Zn-N (outer shell is $3d^{10} 4s^2$) [7, 8]. A comparison of the TGA curves revealed that the ML-ZIF-67's thermolysis temperature was about 50°C ahead than that of the ML-ZIF-8's (**Figure 2**). Overlapping TGA and intensity plot of (011) peaks illustrate that the mixed-ligand ratio cannot influence the thermolysis temperature of NH_2-ZIF67 (**Figures S16c** and **S16d**). It further demonstrates that NH_2-bImand 2-IM in NH_2-ZIF67 have similar thermolysis temperatures and cannot selectively retain/remove one ligand by temperature.

More detail as observed in the ex-situ ATR-IR spectra (**Figure S17**), crystal structure of NH_2-ZIF67 is rapidly converted to cobalt oxide at 10°C (220-230°C). Prior to oxidation, no significant NH_2-bImremoval process was observed, and both ν(Co-$N_β$) and ν(-NH_2) were maintained. After switching to inert gas (Ar) calcination, NH_2-ZIF67 was similarly carbonized rapidly between 440-500°C, resulting in the destruction of the ZIF-67 framework. Once again, ligand thermolysis did not occur prior to 450°C in inert gas flow. In conclusion, the temperatures of the two ligands in NH_2-ZIF67 were too near to each other, regardless of the gas atmosphere, to allow the SeLiRe by thermolysis. This removal strategy, in contrast, can be simply applied to the ML-ZIF-8 which has a suitable temperature window of the two ligands.

Currently, we are focusing on finding ligands that can effectively facilitate the mixing and removal of ligands in a specific type of zeolitic imidazolate framework.

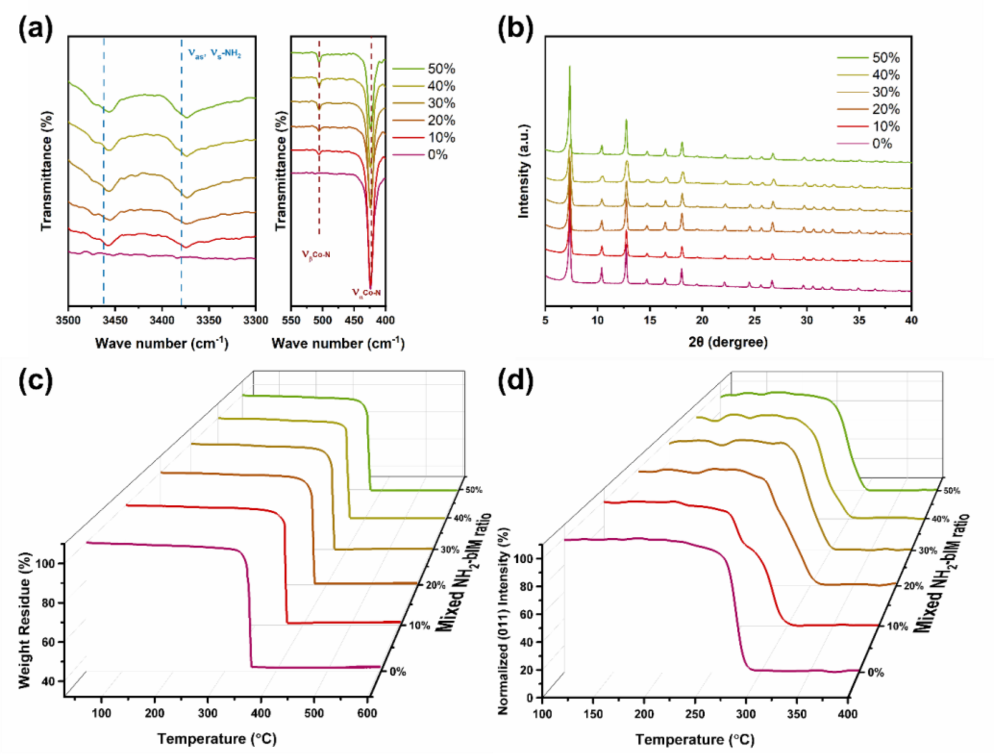

Figure S16. ATR-IR spectra (a) and XRD patterns (b) of 0-50%NH_2-ZIF67. TGA curves of 0-50%NH_2-ZIF67 (c). Intensity evolution plot of (011) peaks in the in-situ XRD with temperature for 0-50%NH_2-ZIF67 (d), data are from the in-situ XRD patterns (**Figure S18**).

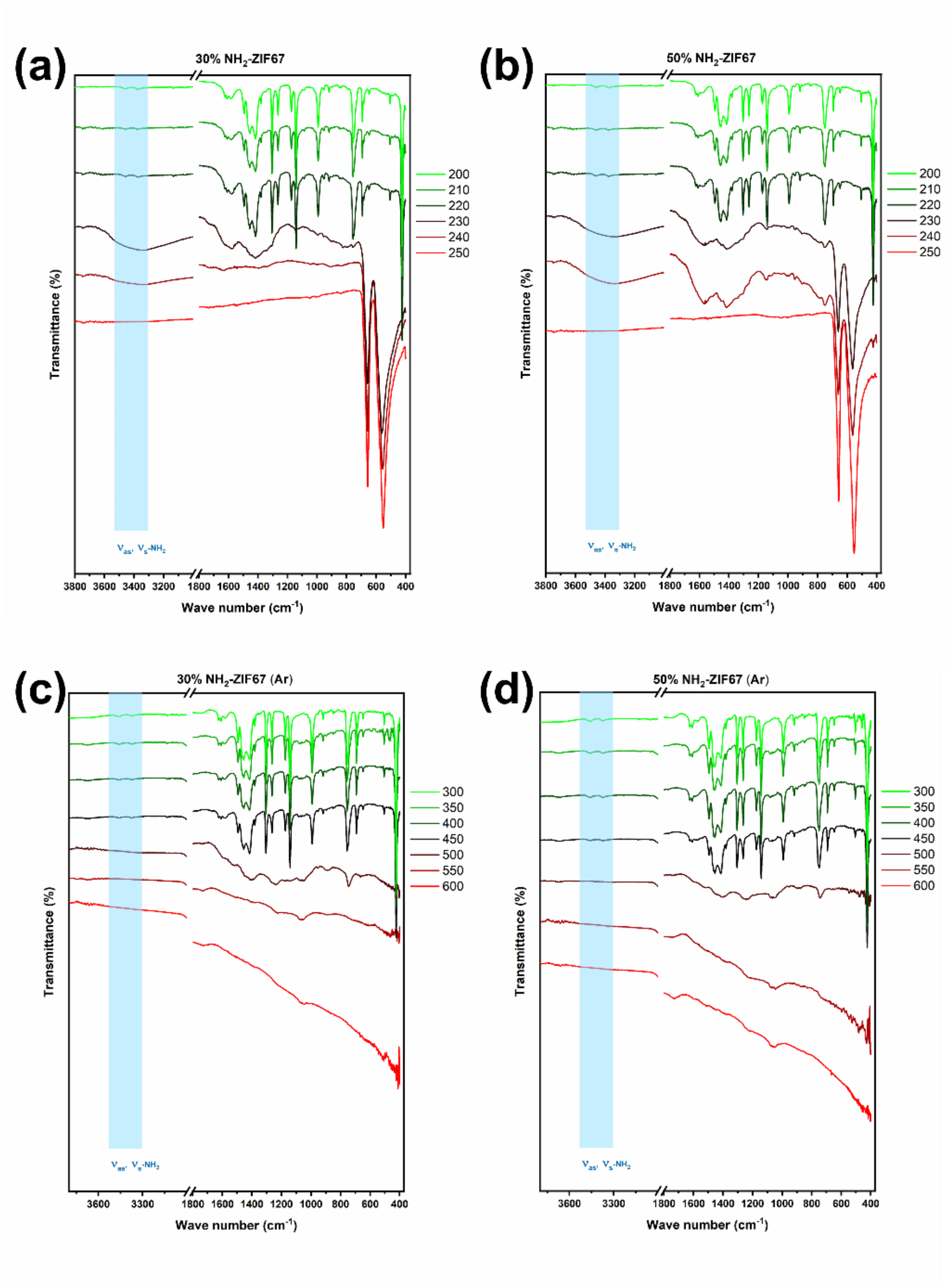

Figure S17. Ex-situ ATR-IR spectra of 30%NH2-ZIF67 (a, in air; c, in Ar) and 50%NH2-ZIF67 (b, in air; d, in Ar) at different temperatures. Calcination time of the all NH2-ZIF67 were two hours, and then cooled naturally to room temperature.

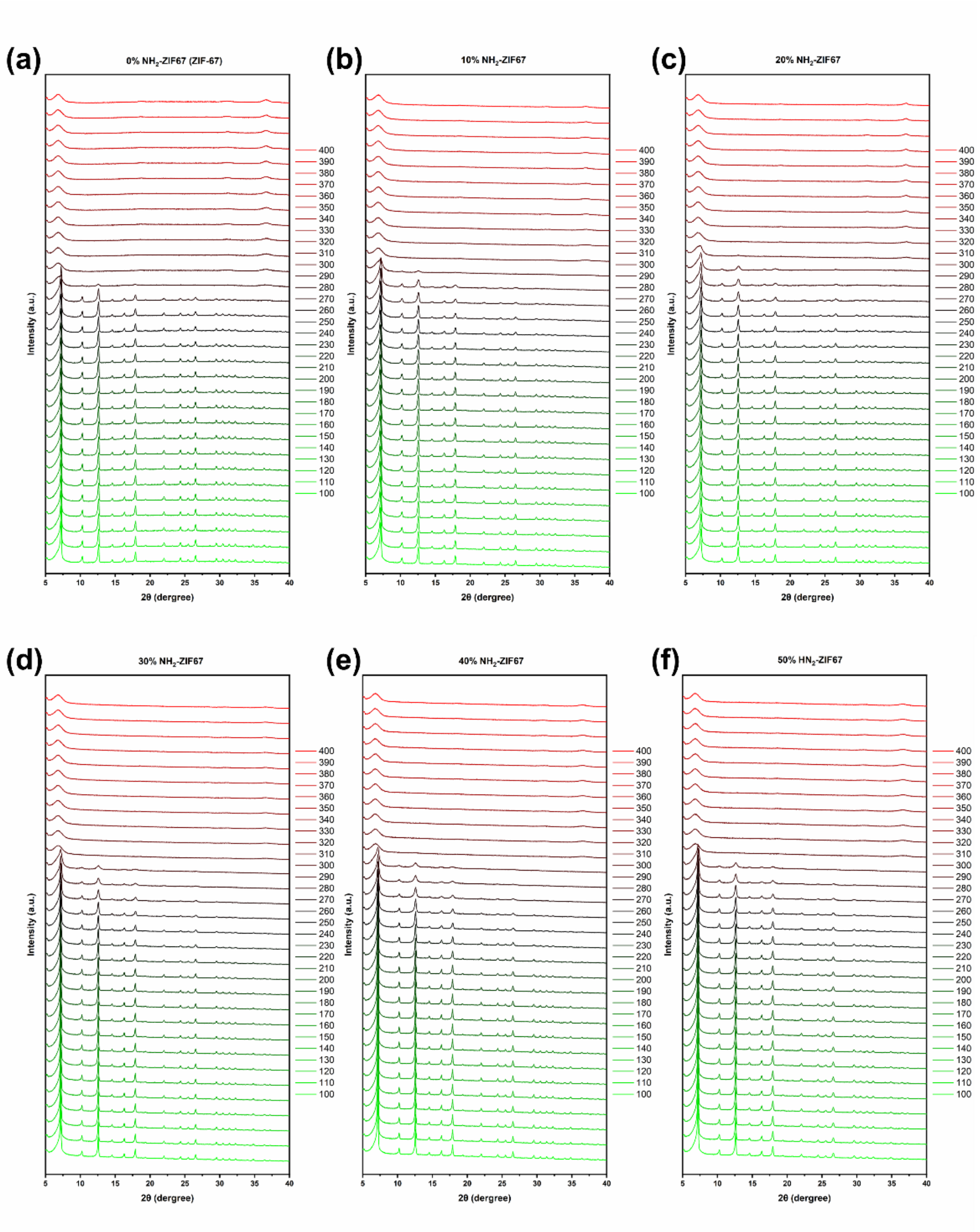

Figure S18. Temperature-programmed in-situ XRD patterns of 0-50%NH_2-ZIF67 in air atmosphere (a, 0%NH_2-ZIF67; b, 10%NH_2-ZIF67; c, 20%NH_2-ZIF67; d, 30%NH_2-ZIF67; e, 40%NH_2-ZIF67; f, 50%NH_2-ZIF67).

S3. Characterization of pore structures

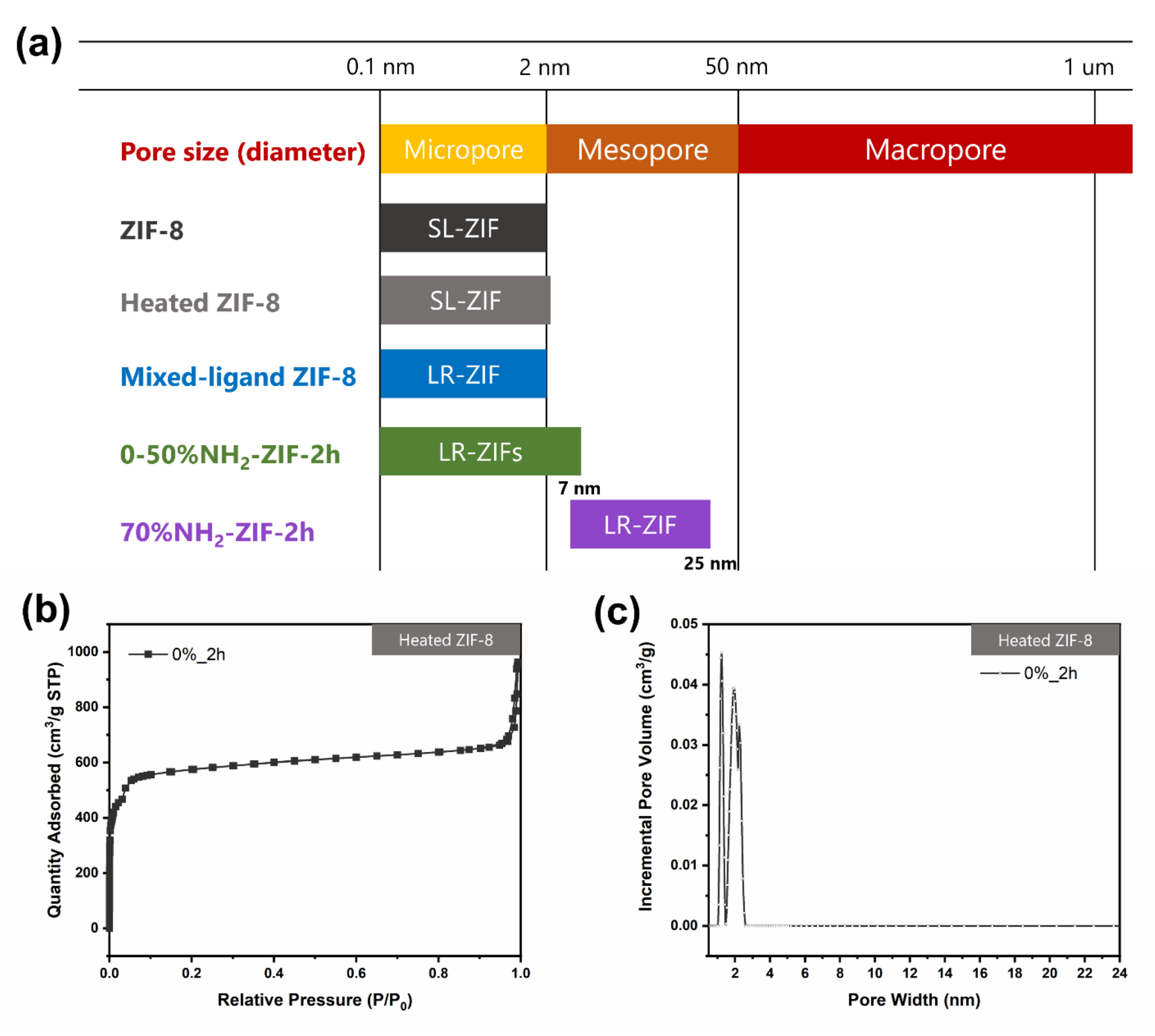

Figure S19. NLDFT porosity distribution diagram of ZIF-8, mixed-ligand ZIF-8, single-ligand ZIF-8 (SL-ZIF) and ligand-removal ZIF-8 (a). Heated ZIF-8 means heating of pure ZIF-8 at 290°C for 2 hours (0%NH_2-ZIF-2h). N_2 sorption isotherm (b) and NLDFT porosity distribution (c) of heated ZIF-8. Despite the impact of heating on the pore distribution of SL-ZIF, it still remains concentrated within the microporous range, indicating the advantage of ML-ZIFs in constructing tuned porous structures through thermolysis.

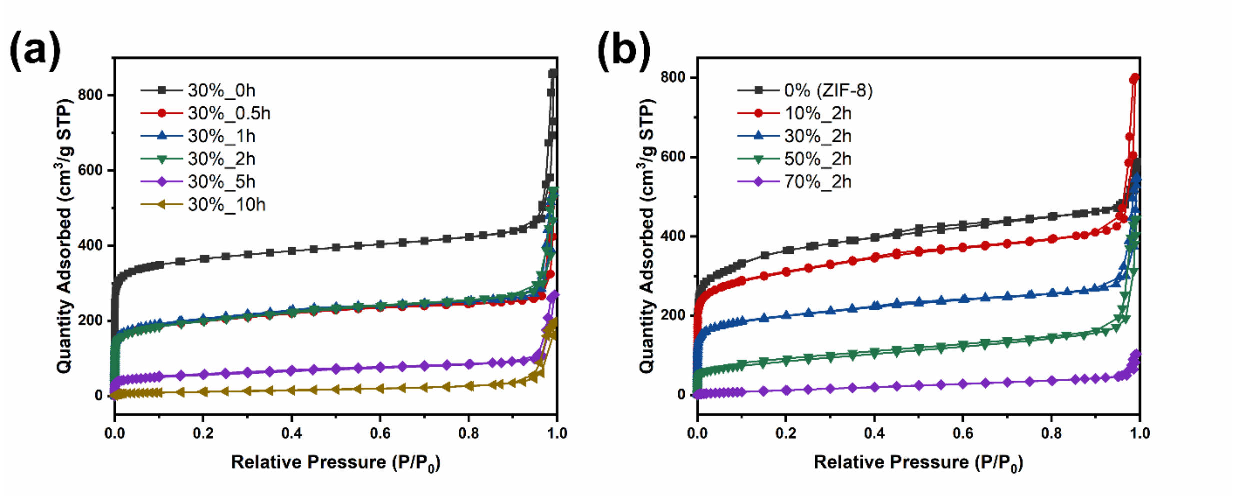

Figure S20. N_2 physisorption isotherms at 77 K of 30%NH_2-ZIF-*time* at 0-10 hours calcination times (a) and R%NH_2-ZIF-2h including ZIF-8 (b).

Table S2. Specific surface area and porosity parameters of ML-ZIFs and LR-ZIFs.

**Samples**[a]	**Specific surface area**[b] **($m^2\ g^{-1}$)**	**Total Pore Volume ($cm^3\ g^{-1}$)**	**Micropore Volume**[c] **($cm^3\ g^{-1}$)**	**Mesopore Volume**[d] **($cm^3\ g^{-1}$)**
0%NH_2-ZIF-0h (ZIF-8)	1315.0	0.518	0.518	-
10%NH_2-ZIF-2h	999.6	0.392	0.376	0.016
30%NH_2-ZIF-0h	1192.9	0.499	0.499	-
30%NH_2-ZIF-0.5h	733.3	0.287	0.246	0.041
30%NH_2-ZIF-1h	760.9	0.292	0.243	0.049
30%NH_2-ZIF-2h	665.0	0.309	0.240	0.067
30%NH_2-ZIF-5h	199.7	0.075	0.044	0.031
30%NH_2-ZIF-10h	43.5	0.005	-	0.005
50%NH_2-ZIF-2h	296.8	0.131	0.045	0.086
70%NH_2-ZIF-2h	60.1	0.038	-	0.038
30%NH_2-ZIF-Ar-2h	1180.3	0.540	0.342	0.198
70%NH_2-ZIF-Ar-2h	79.5	0.072	-	0.072

[a] In the air atmosphere range, 2-50% heated at 290°C and 70% heated at 260°C. In the Ar flow, 30%NH_2-ZIF heated to 500°C, 70%NH_2-ZIF heated to 400°C.

[b] Brunauer-Emmett-Teller specific surface area.

[c] Cumulative adsorption volume of micropores from 0 to 2 nm in diameter.

[d] Cumulative adsorption volume of mesopores from 2 to 50 nm in diameter according to International Union of Pure and Applied Chemistry (IUPAC) [9].

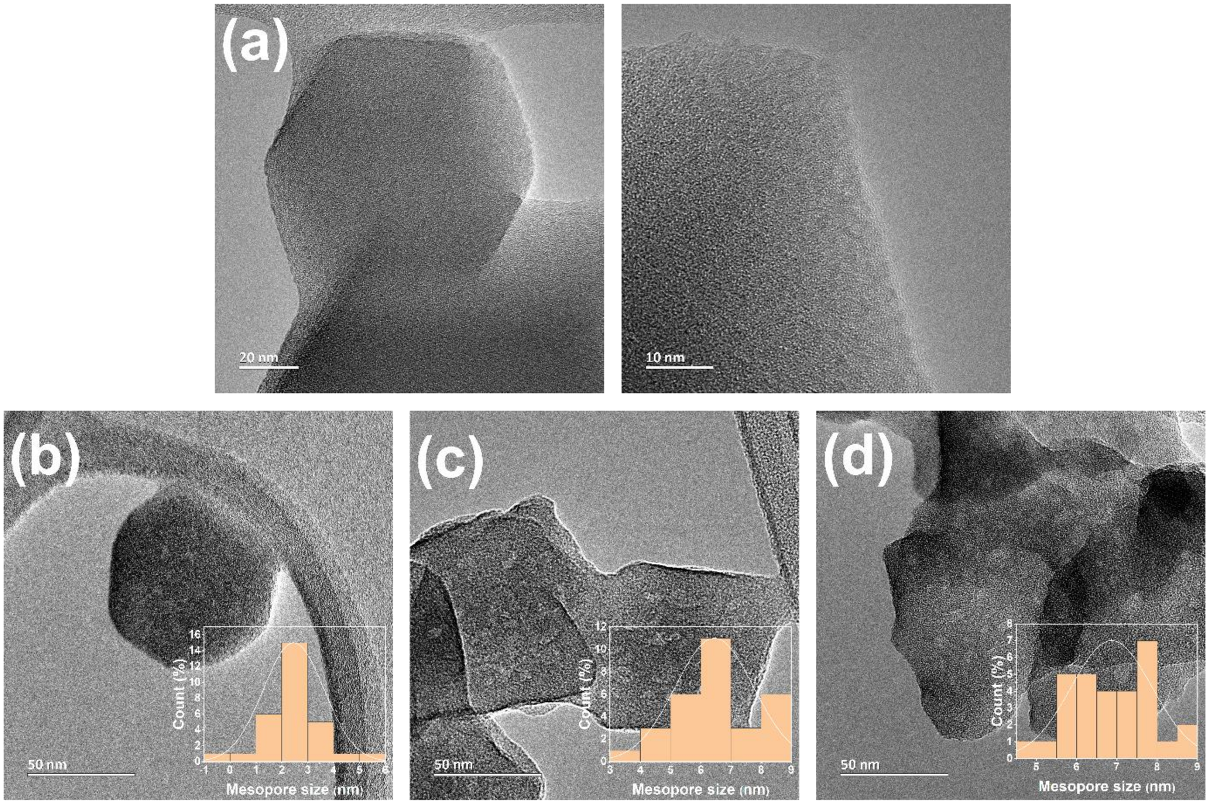

Figure S21. TEM images of pure ZIF-8 (a), 10%NH_2-ZIF-2h (b), 50%NH_2-ZIF-2h (c) and 70%NH_2-ZIF-2h (d). The inside shows the distribution of mesopore size.

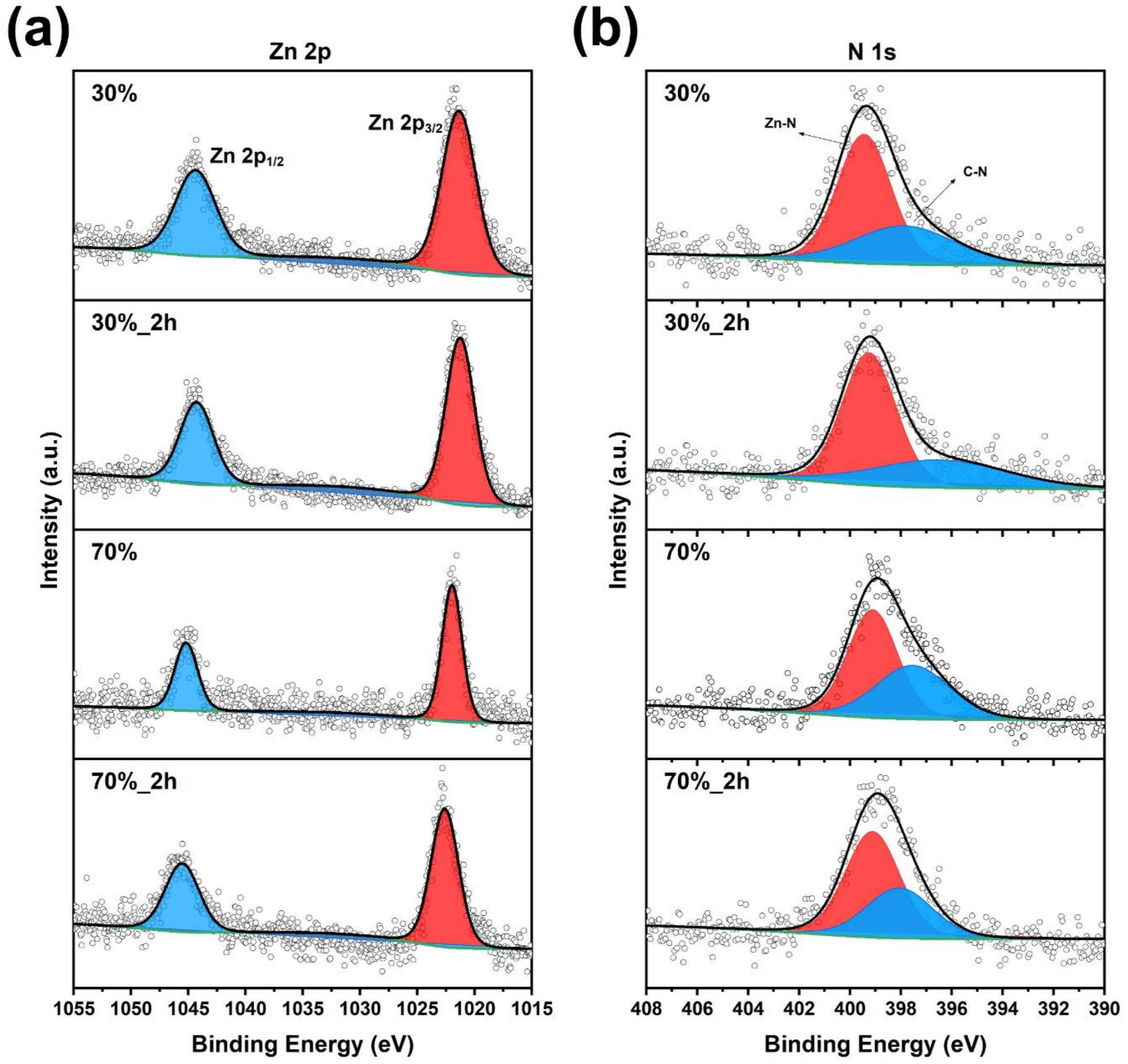

Figure S22. Zn 2p (a) and N 1s (b) spectrum of XPS for 30%NH_2-ZIF, 30%NH_2-ZIF-2h, 70%NH_2-ZIF and 70%NH_2-ZIF-2h. N1 s fitting has two peaks; the first one is assigned to N atom of imidazole linkers at 398.01 eV and the second belongs to nitrogen atom at Zn-N bond at 398.61 eV [10].

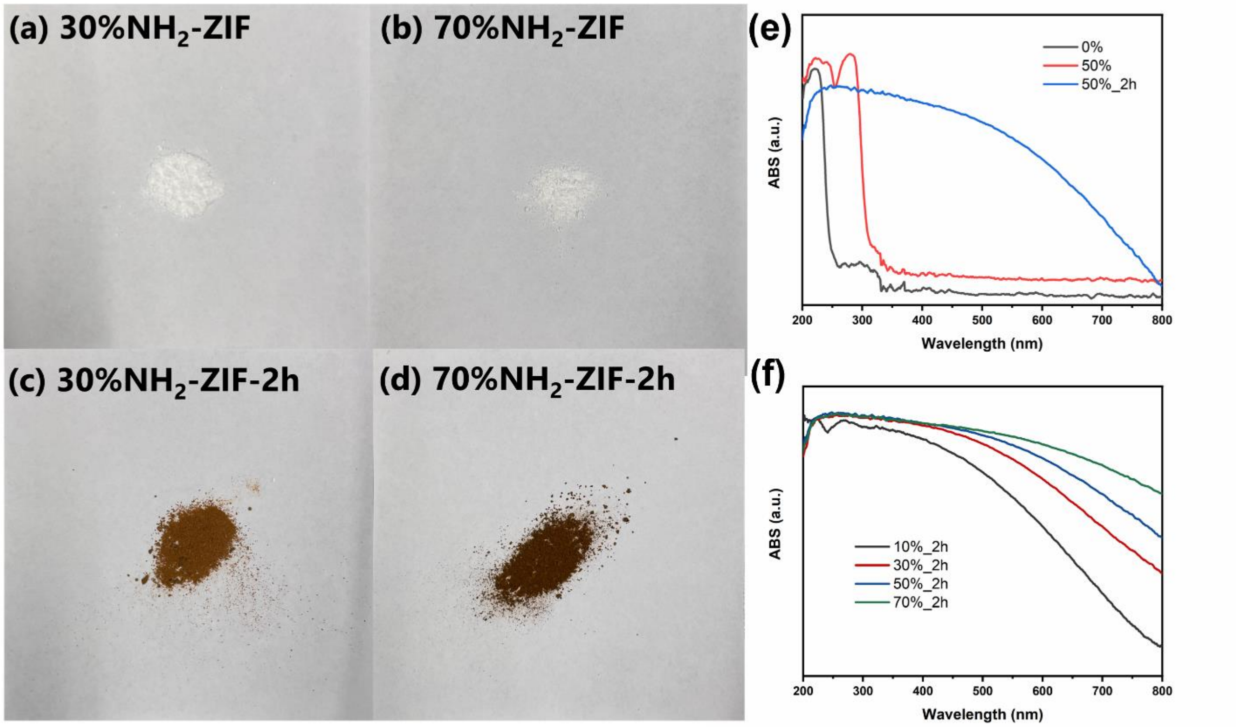

Figure S23. Optical photographs of ML-ZIFs (a, 30%NH_2-ZIF; b, 70%NH_2-ZIF) and LR-ZIFs (d, 30%NH_2-ZIF-2h; e, 70%NH_2-ZIF-2h). Powder DRS for 0%NH_2-ZIF (ZIF-8), 50%NH_2-ZIF-0h and 50%NH_2-ZIF-2h (g), and various ratios of ligands 10-70% removal of R%NH_2-ZIF-2h (h). As the ratio of NH_2-bIm increases, the color of NH_2-ZIF-2h gradually deepens (from light brown to dark brown) after ligand thermolysis. This color change can be attributed to the oxidation of the amino group in NH_2-bIM; the sample's color often changes to brown when nitrogen-containing compounds are oxidized [3, 11].

S4. Ligand removal in inert gases

Structural characterization of LR-ZIFs. To investigate the effect of inert gas on ligand thermolysis, 30% and 70%NH_2-ZIF were calcined in a tube furnace with an Ar flow. As the temperature rise, the $\nu_{s,as}$(-NH_2) and ν(Zn-N_β) of NH_2-ZIF both dropped to 0% in the ex-situ AIR-IR spectra (**Figures S24a** and **S24b**). These bands exhibit reduction between 450-500°C in 30%NH_2-ZIF and 300-450°C in 70%NH_2-ZIF.

In ^{1}H NMR, the thermolabile ligand was almost completely removed from 30%NH_2-ZIF-2h (Ar), while the ligand was only partially removed from 70%NH_2-ZIF-2h (Ar) due to the lower thermolysis temperature (**Figure S25**). Like the thermolysis in air, the characteristic quartets of NH_2-bIm on both sides of 7.35 ppm was markedly reduced. Among them, the NH_2-bIm of 30%NH_2-ZIF-2h (Ar) was more sufficiently removed due to the higher temperature of 500°C, with almost no quadruple peaks remaining. However, due to the excessive mixing of thermolabile ligands in 70%NH_2-ZIF-2h (Ar), it could only maintain its crystallinity at 400°C, resulting in incomplete ligand removal. The *by-products, in contrast to thermolysis in air, from oxidation were not observed.

As well, ligand thermolysis can also be achieved using N_2 flow, with no difference compared to Ar flow. Whether in N_2 or Ar, in-situ XRD illustrates that the crystallinity of ML-ZIF decreased with temperature, while 0%, 30% and 70%NH_2-ZIF collapsed after 450°C, 520°C and 550°C respectively (**Figures S24c** and **S24d**). Similarly, calcination times of >2 hours have the devastating effect on the crystallinity of 30% and 70%NH_2-ZIF (**Figure S28**). If the temperature continues to be raised to 700-900°C, the zinc will evaporate and eventually form the carbon material like nitrogen-doped carbon (NC) or graphitic carbon (GC) [12, 13], which is detrimental for our research. Additionally, the color of LR-ZIF (Ar) tends to be darker due to the additional carbon attached, and the light absorption extends from the UV to the visible region (**Figure S29**).

Hierarchical Pores in LR-ZIFs (Ar). NLDFT porosity distribution and TEM images illustrate the feasibility of constructing sponge-like hierarchical porous ZIFs by SeLiRe process (**Figures S30** and **S31**). Compared to air, 30%NH_2-ZIF-Ar-2h (500°C) exhibits bimodal distribution of mesopores,

ranging in size from 2 to 7 nm (close to the 50% in air). In contrast, 70%NH_2-ZIF-Ar-2h (400°C) obtained by the lower temperature, is almost identical to the calcined sample in air, strongly supporting our hypothesis regarding the SeLiRe mechanism. Note that single-ligand ZIFs also can gradually dislodge the intrinsic metal clusters and thus form the irregular mesopores during high-temperature calcination with inert gases [14, 15]. However, they typically fail to maintain the intrinsic micropores and crystal structure of ZIFs, eventually converting to NC or GC [16]. Moreover, compared with ML-ZIFs, single-ligand ZIFs heating is difficult to regulate the porous channel and spatial arrangement of mesopores through the synthetic condition. Our focus in this work is on the construction of controlled hierarchically porous ZIFs that maintain the intrinsic framework structure, thus making single-ligand ZIFs as a precursor less desirable for our purposes.

Influence of inert gas calcination. Compared to air calcination, the use of inert gas allowed for SeLiRe at higher temperatures, up to 400-500°C, while avoiding the by-products and influence of oxidation reaction on the ML-ZIFs. The surface of the ML-ZIF was the first to be affected by high temperature, tending to carbonize at temperatures over 500°C. The appearance of two bands, D band (1342 cm^{-1}) and G band (1577 cm^{-1}) corresponded to the disordered carbon atoms and the sp^2-hybridised graphite carbon atoms, respectively (**Figure S32d**) [17]. These bands indicate the formation of the graphite/carbon structure resulting from the calcination in inert gas. However, the XRD, ATR-IR, XPS and TGA did not change much, indicating the maintain of the framework structure within ZIF-8 (**Figures S32a-c**). Therefore, the removal of NH_2-bIm does not depend on the oxidation reaction, and the SeLiRe process can be achieved in inert gas as well.

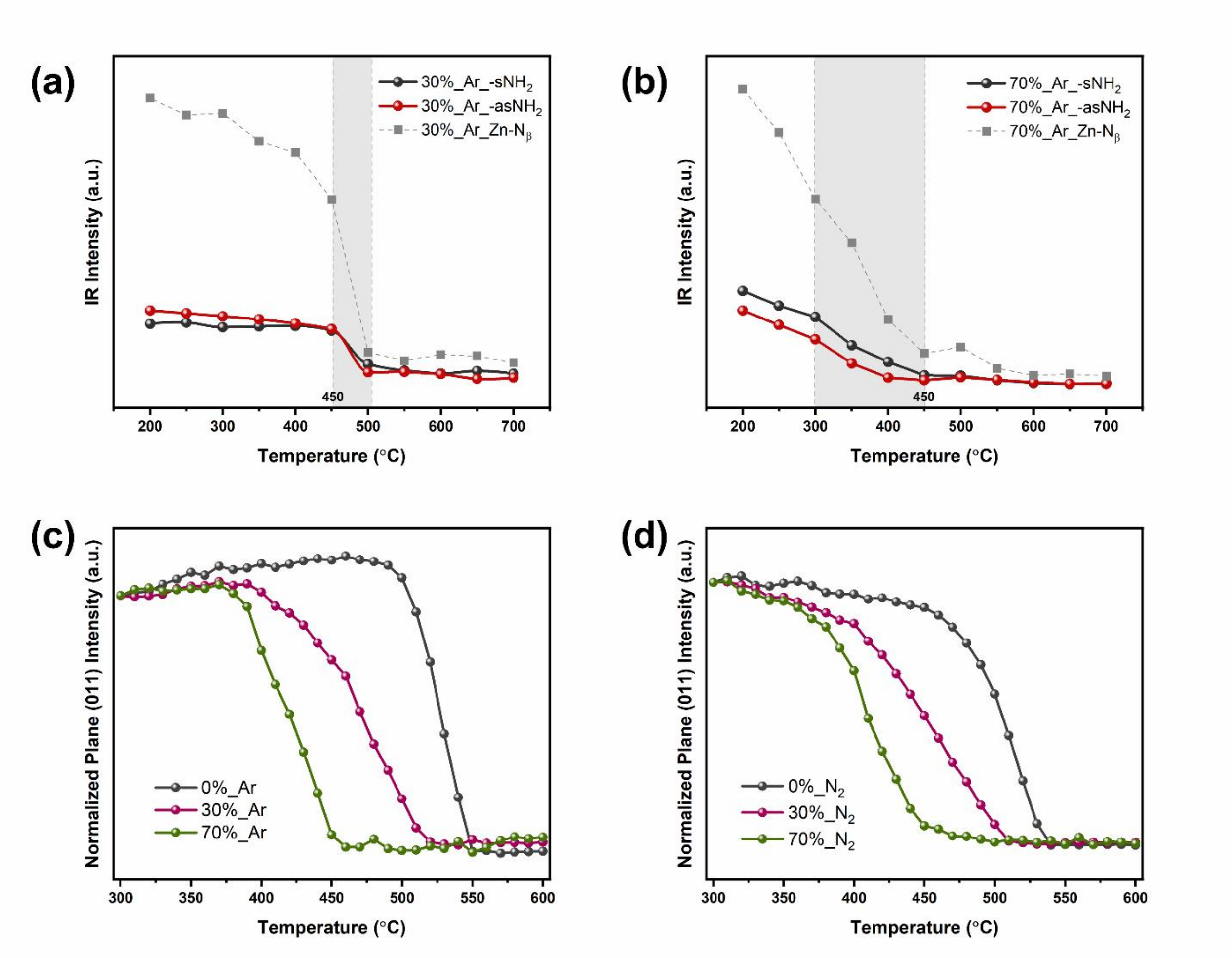

Figure S24. Intensity plot of $\nu_{s,\,as}$(-NH_2) and ν(Zn-N_β) in the ex-situ AIR-IR spectra with temperature for 30% (a) and 70%NH_2-ZIF (b) in Ar atmosphere. Data are from the IR spectra in **Figure S26** at 3250-3550 cm^{-1} for ν(-NH_2), 400-550 cm^{-1} for ν(Zn-N_β). Evolution intensity plot of (011) peaks in the in-situ XRD for 0%, 30%, 70%NH_2-ZIF in Ar (c) and N_2 (d) flow. Data are from the XRD patterns of (011) peaks in **Figure S27**.

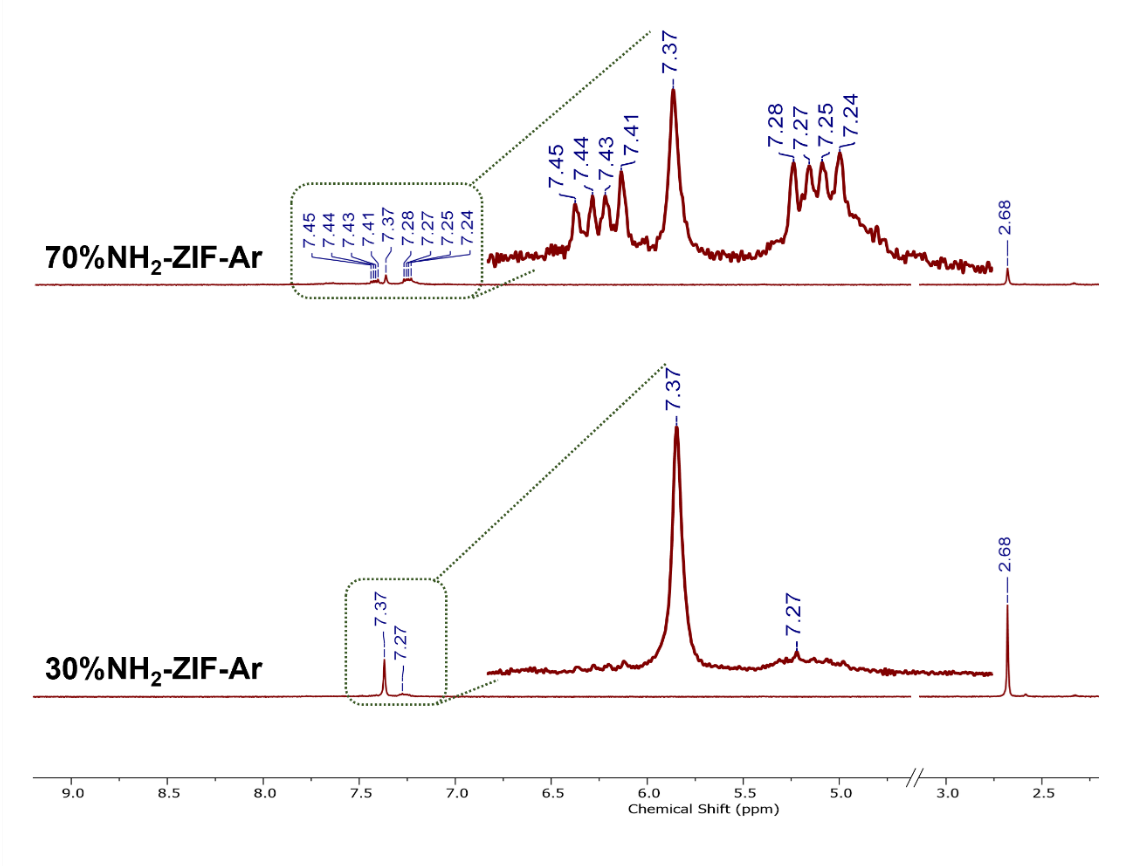

Figure S25. ^{1}H NMR spectroscopy of 30%NH_2-ZIF-Ar-2h and 70%NH_2-ZIF-Ar-2h in Ar atmosphere.

30%NH_2-ZIF-Ar-2h: *^{1}H NMR (250 MHz, Acetic Acid-d_4) δ 2.60 – 2.80 (s, 14H), 7.24 – 7.31 (s, 1H), 7.33 – 7.41 (s, 8H).* 70%NH_2-ZIF-Ar-2h: *^{1}H NMR (250 MHz, Acetic Acid-d_4) δ 2.58 – 2.79 (s, 2H), 7.17 – 7.31 (dd, J = 3.3, 5.9 Hz, 2H), 7.31 – 7.40 (s, 1H), 7.40 – 7.51 (dd, J = 3.3, 5.9 Hz, 1H).*

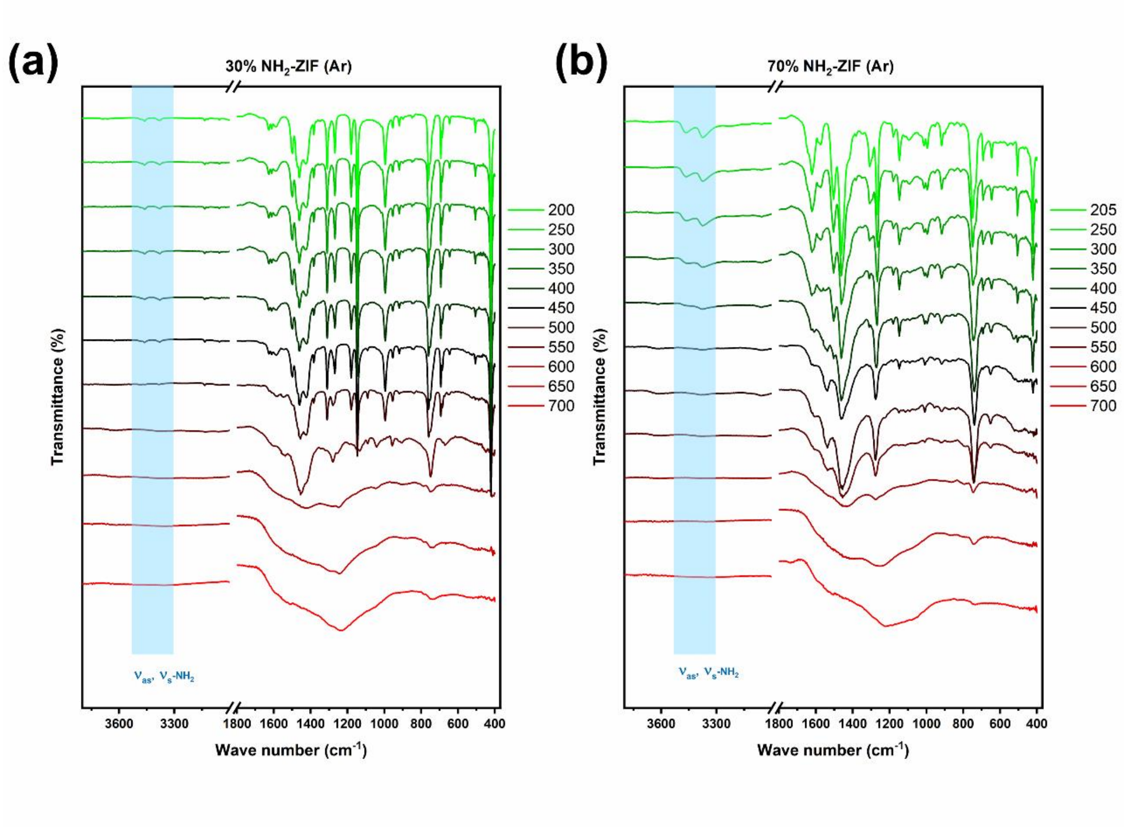

Figure S26. Ex-situ ATR-IR spectra of 30%NH_2-ZIF (a) and 70%NH_2-ZIF (b) at different temperatures. All ML-ZIFs were calcined in a tube furnace, in Ar atmosphere, for a period of 2 hours and then cooled naturally to room temperature.

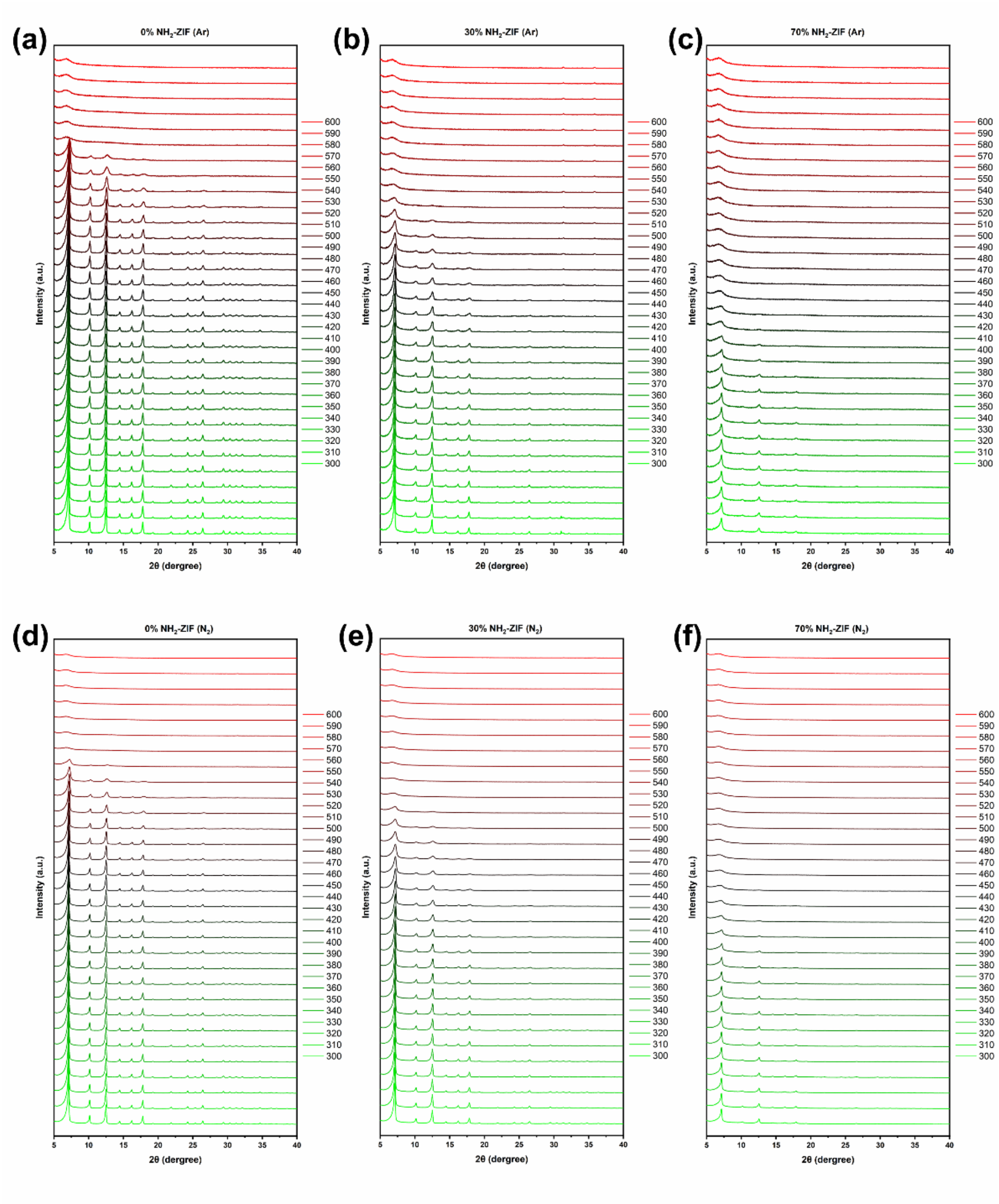

Figure S27. Temperature-programmed in-situ XRD patterns of 0%, 30% and 70%NH$_2$-ZIFs in Ar flow (a, 0%NH$_2$-ZIF; b, 30%NH$_2$-ZIF; c, 70%NH$_2$-ZIF) and in N_2 flow (d, 0%NH$_2$-ZIF; e, 30%NH$_2$-ZIF; f, 70%NH$_2$-ZIF).

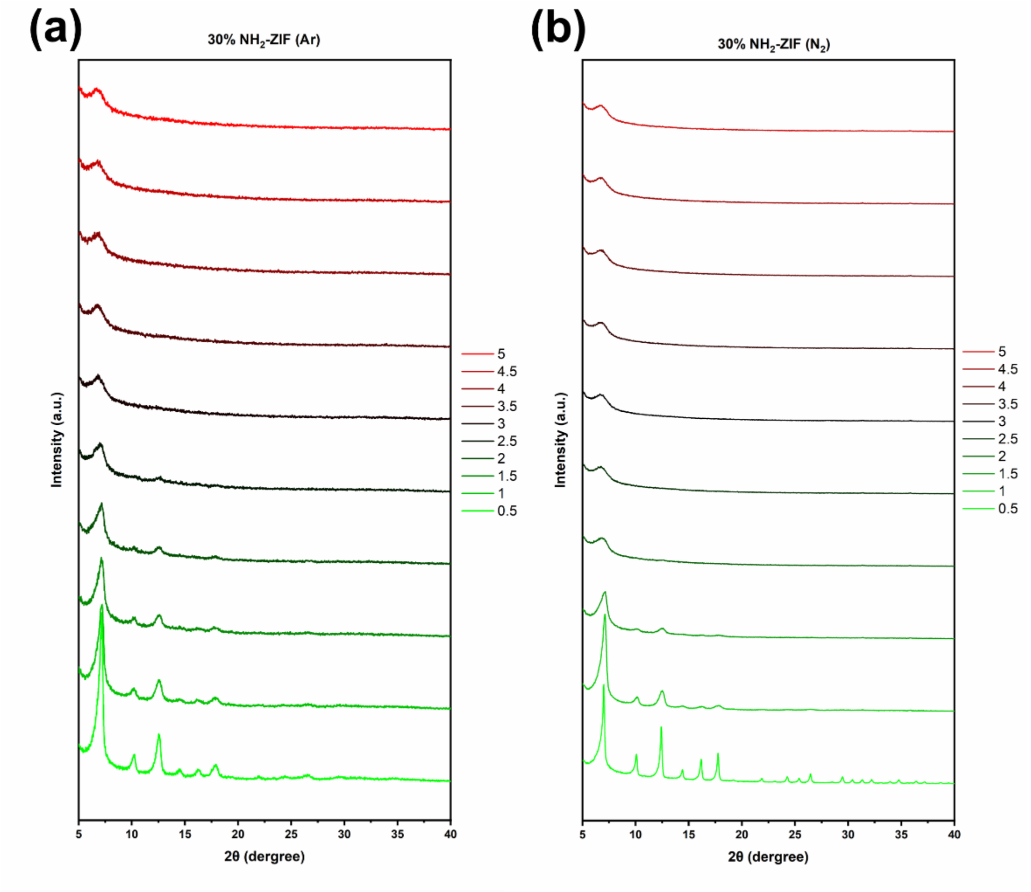

Figure S28. Isothermal in-situ XRD patterns of 30%NH_2-ZIF at different heating times in Ar (a) and N_2 (b) flow at 500°C.

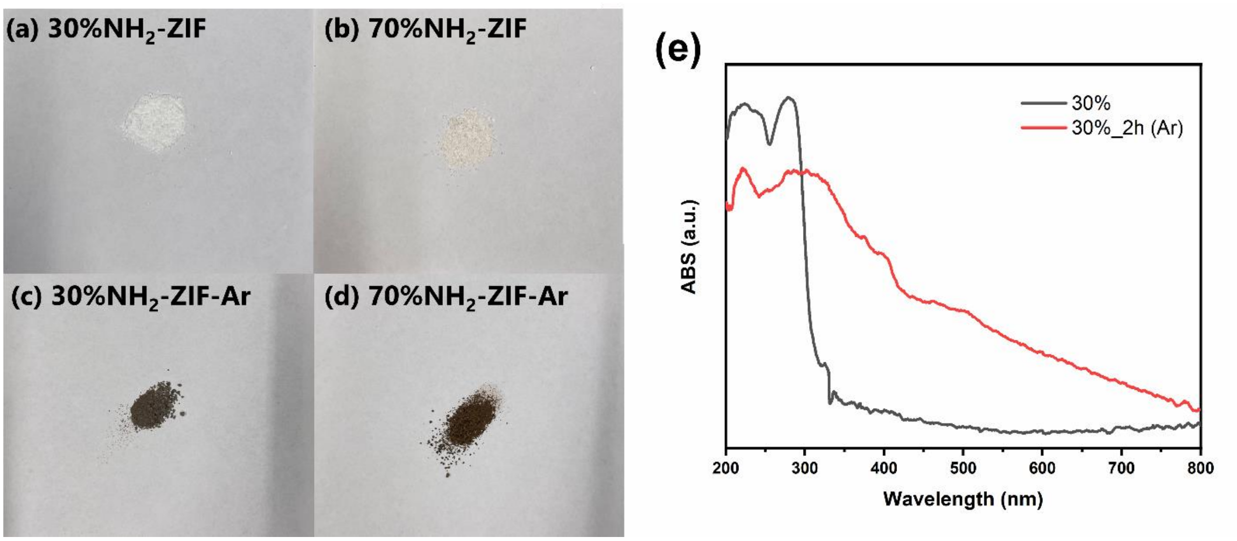

Figure S29. Optical photographs of ML-ZIFs (a, 30%NH_2-ZIF; b, 70%NH_2-ZIF) and LR-ZIFs (c, 30%NH_2-ZIF-Ar-2h; d, 70%NH_2-ZIF-Ar-2h). Powder DRS for 30%NH_2-ZIF and 30%NH_2-ZIF-Ar-2h (e).

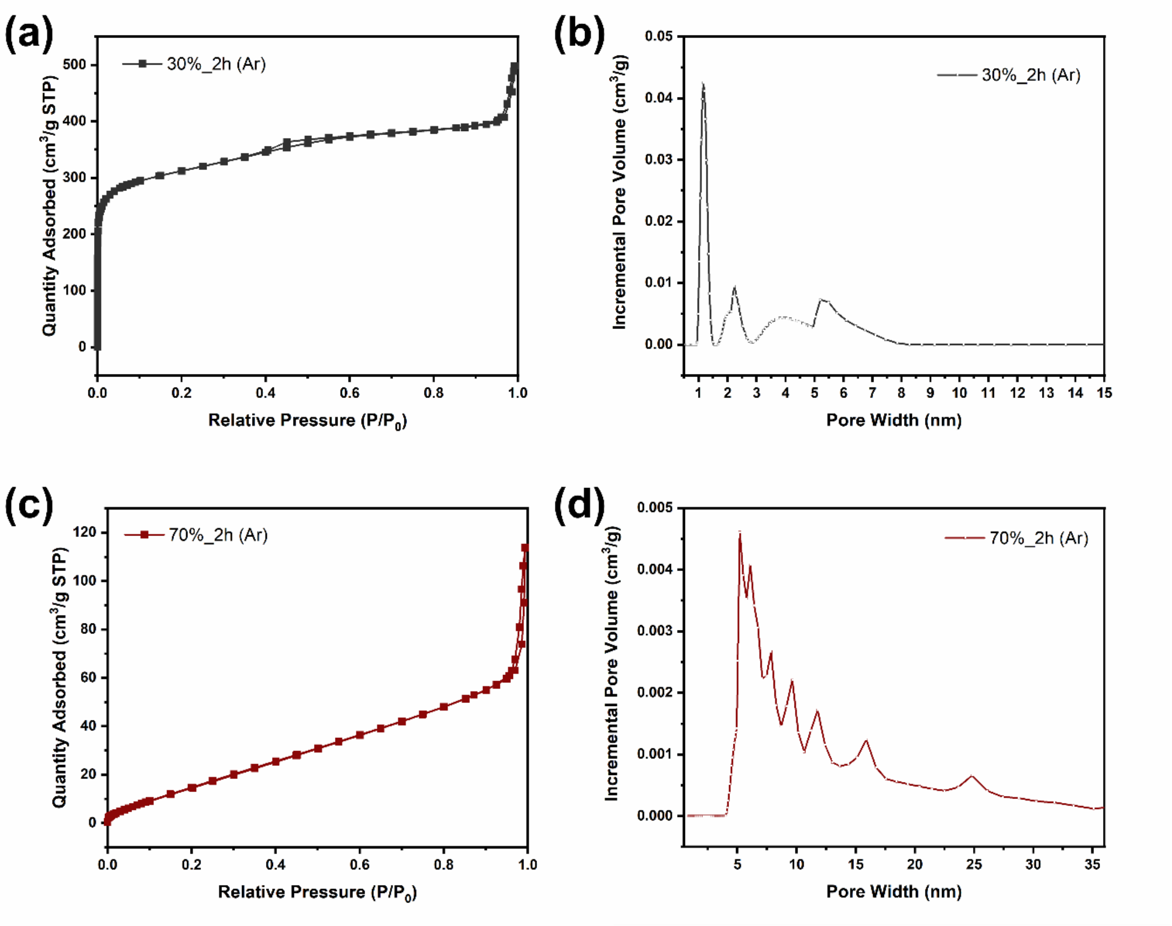

Figure S30. N_2 sorption isotherm (a) and NLDFT porosity distribution (b) of 30%NH_2-ZIF-Ar-2h. N_2 sorption isotherm (c) and NLDFT porosity distribution (d) of 70%NH_2-ZIF-Ar-2h.

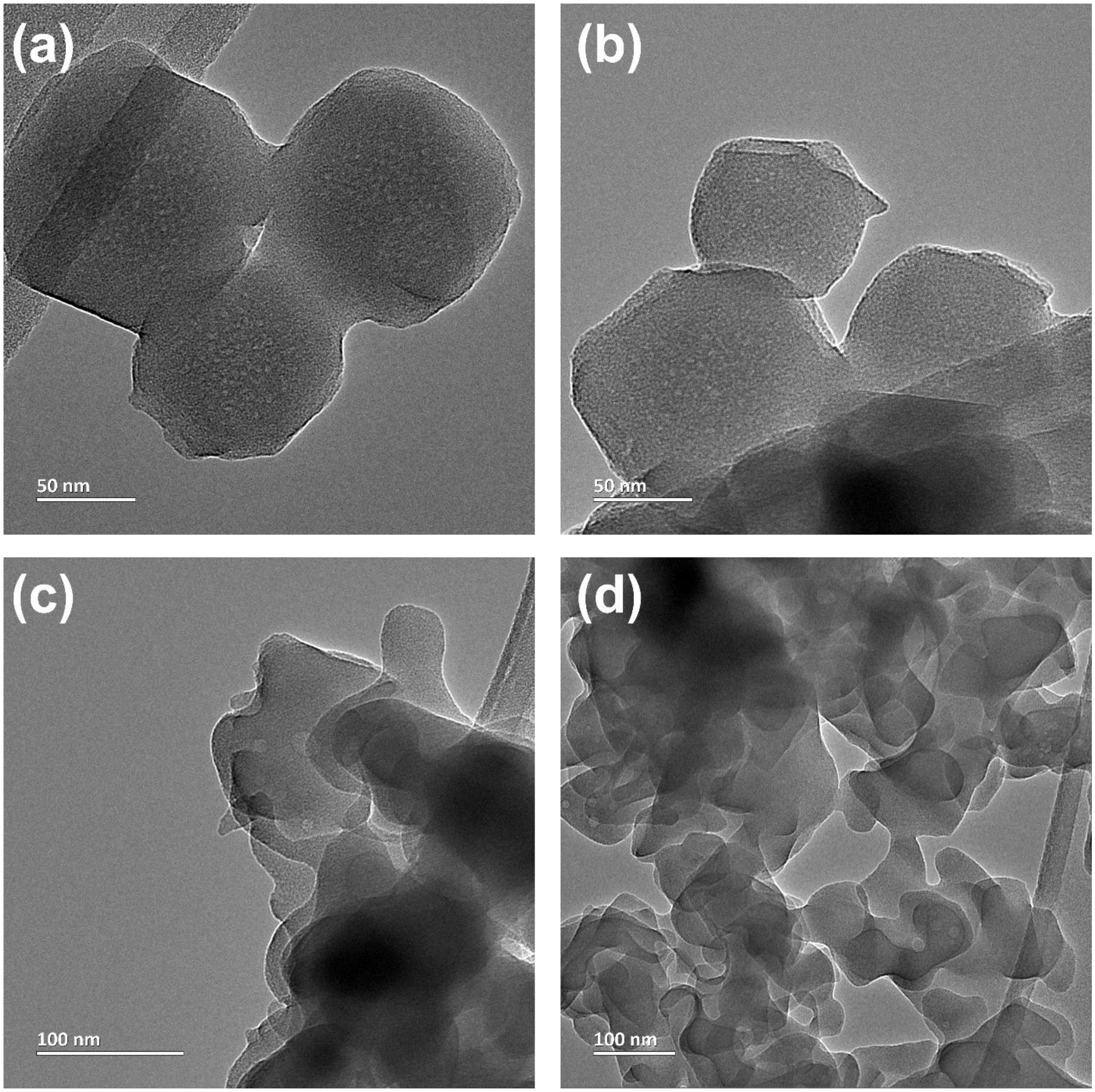

Figure S31. TEM images for single crystals of 30%NH_2-ZIF-Ar-2h (a, b) and 70%NH_2-ZIF-Ar-2h (c, d) after heating treatment in Ar flow.

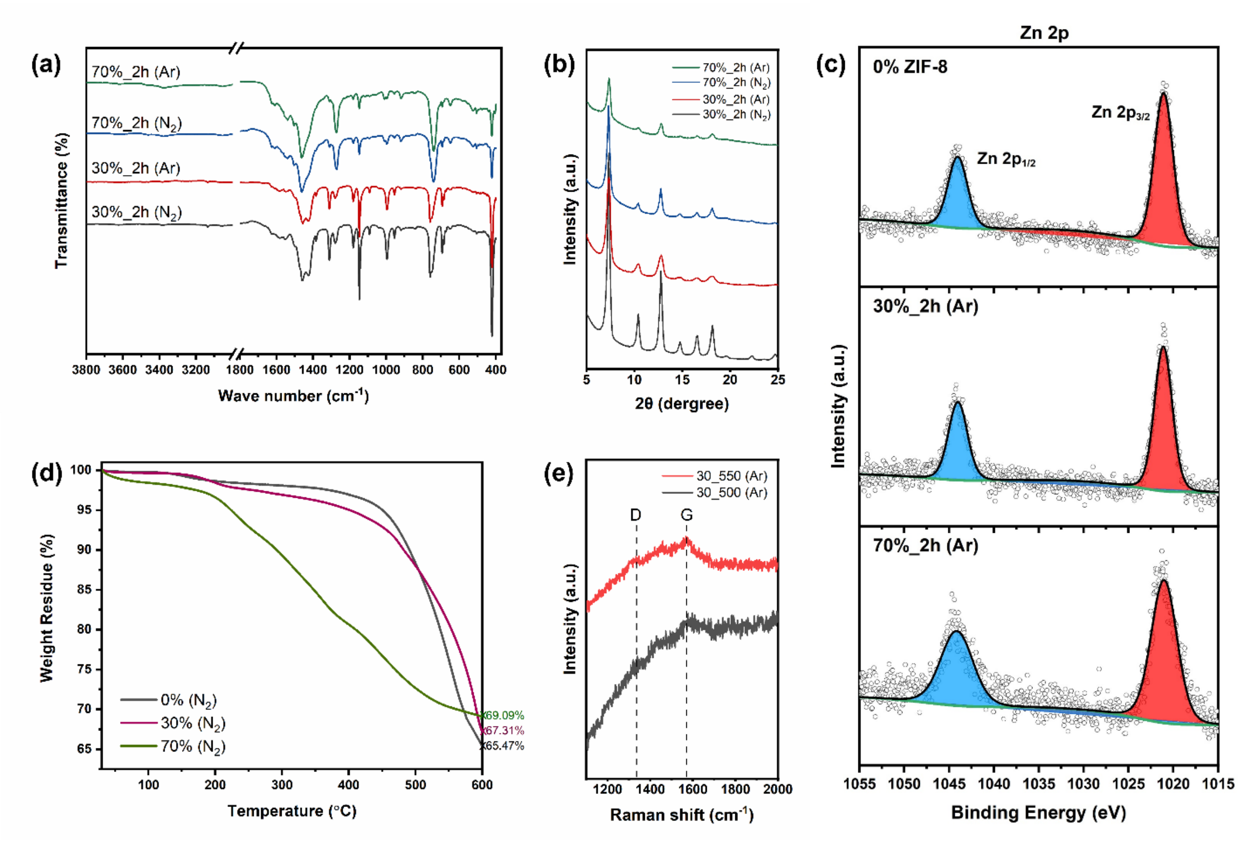

Figure S32. Ex-situ ATR-IR spectra of 30%NH_2-ZIF-2h and 70%NH_2-ZIF-2h were heated in Ar and N_2 atmosphere, respectively (a). XRD patterns of 30%NH_2-ZIF-2h and 70%NH_2-ZIF-2h in Ar and N_2 atmosphere (b). Zn 2p spectrum (c) of XPS for ZIF-8, 30%NH_2-ZIF-Ar-2h and 70%NH_2-ZIF-Ar-2h. TGA curves of 0%NH_2-ZIF (ZIF-8), 30%NH_2-ZIF and 70%NH_2-ZIF were heated in N_2 flow (d). Raman spectra of 30%NH_2-ZIF-500°C and 30%NH_2-ZIF-550°C (e), were heated 2 hours in Ar flow.

S5. DFT simulation models

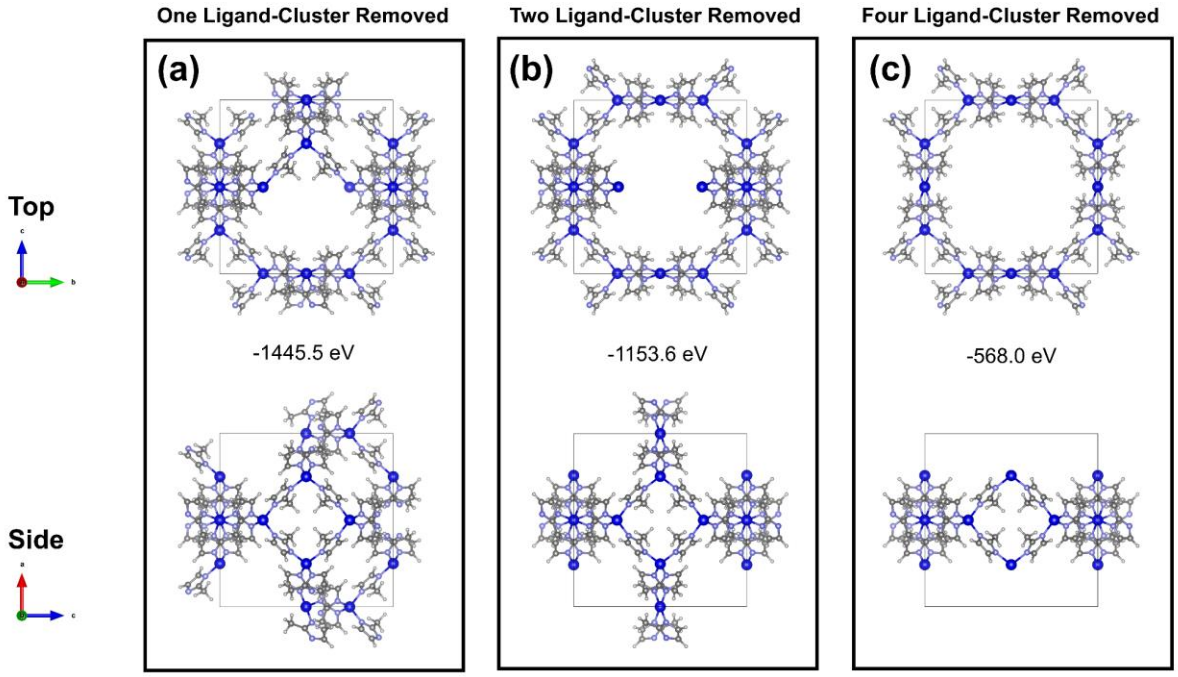

Figure S33. Schematic diagrams (top and side view) of the LR-ZIFs framework with one (a), two (b) and four (c) Zn-N_4 clusters and surrounding ligands removed. Original ZIF-8 and ligand-cluster structures are in **Figure S34**. Dark blue for Zn, light blue for N, gray for C and white for H.

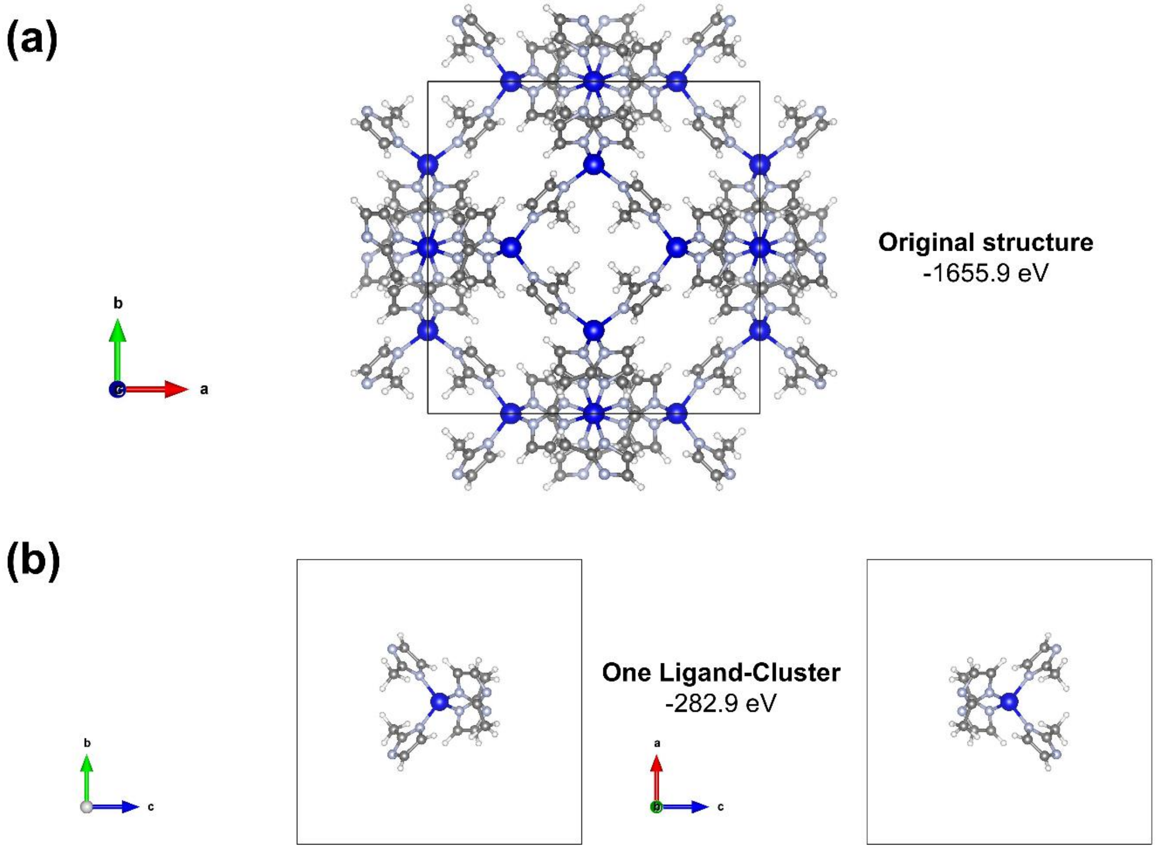

Figure S34. Schematic diagrams of the ZIF-8 original framework (a) and one Zn-$(IM)_4$ ligand-cluster structure (b). Dark blue for Zn, light blue for N, gray for C and white for H.

Table S3. Structural energy of ligand-cluster, ZIF-8 and LR-ZIFs obtained from the DFT calculation.

**Number of ligand-clusters removal**	**Atomic number: H, C, N, Zn**	**E_o [a] (eV)**	**E_f [b] (eV)**
Original (zero)[c]	120,96,48,12	-1740.1688	-
Remove one	100,80,40,11	-1445.4944	11.7447
Remove two	80,64,32,10	-1153.6422	8.9225
Remove four	40,32,16,8	-567.9773	19.8055
One ligand-cluster	20,16,8,1	-282.9297	-

[a] The initial energy of each system obtained after the DFT calculation.

[b] Formation energy calculated by:

Structural energy with ligand-cluster removed + ligand-cluster energy − structural energy without ligand-cluster removed.

For example, the formation energy of structure with two ligand-clusters removed is: structural energy with two ligand-cluster removed + ligand-cluster energy − structural energy with one ligand-cluster removed.

[c] Original ZIF-8 in the **Figure S34a**.

S6. Enhancing Dyes Adsorption

Evaluation of the adsorption experiment. From kinetic and isotherm curves of MB adsorption (**Figures S35** and **S36)**, it was clearly evident that pure ZIF-8 had essentially little capacity for MB adsorption, and with increase after mixing with the ligand NH_2-bIM. Thankfully, once the ligand was removed, the adsorption capacity of LR-ZIF-8 was considerably enhanced. After fitting all isotherms curves, it was shown that the R^2 values for ML-ZIFs were higher in the Langmuir model than the Freundlich model, indicating their favoring for monolayer adsorption (**Table S5**) [18]. In the kinetic fit, the R^2 values for the PSO model were higher than those for the PFO model, as explained in detail in the main manuscript (**Table S4**).

If the calcination time is 2h, the ranking of the adsorption capacity Q_m is calculated to be 10%NH_2-ZIF-2h > 30%NH_2-ZIF-2h > 50%NH_2-ZIF-2h > 5%NH_2-ZIF-2h > 2%NH_2-ZIF-2h > 70%NH_2-ZIF-2h. Among them, 10%NH_2-ZIF-2h showed the highest capacity (Q_m is 20.835 mg/g) for MB adsorption, 39 times that of pure ZIF-8 (0.531 mg/g) and 5.5 times that of the 10%ML-ZIF (3.874 mg/g), a significant improvement in adsorption capacity. When the mixed-ligand ratio is 10%, the ranking of the different calcination time was 10%NH_2-ZIF-2h > 10%NH_2-ZIF-0.5h > 10%NH_2-ZIF-5h > 10%NH_2-ZIF-20h > 10%NH_2-ZIF-0h. The calcination time beyond 5 hours causes irreversible effects on the NH_2-ZIF's framework, leading to the loss of active sites as a result of hierarchical pores damage/over-oxidation, ultimately led to a dramatic decrease in the 10%NH_2-ZIF-20h (3.931 mg/g). For the same reason, the adsorption capacity of 10%NH_2-ZIF-2h (Ar) was not sufficiently enhanced when heated at higher temperature (in 500°C, **Figure S37c**).

Adsorption of other organic dyes. To further evaluate the water purification of LR-ZIF-8, we conducted experiments with other organic dyes (**Figures S37a** and **S37b**). In the adsorption test for methyl orange, the Q_e of 10%NH_2-ZIF-2h was 19.053 mg/g, again a great increase in adsorption capacity compared to the 4.226 mg/g of 10%NH_2-ZIF-0h, approximately 5 times. Similarly, for Rhodamine B, the Q_e of 10%NH_2-ZIF-2h (23.955 mg/g) was significantly higher than that of 10%NH_2-ZIF-0h (14.584 mg/g).

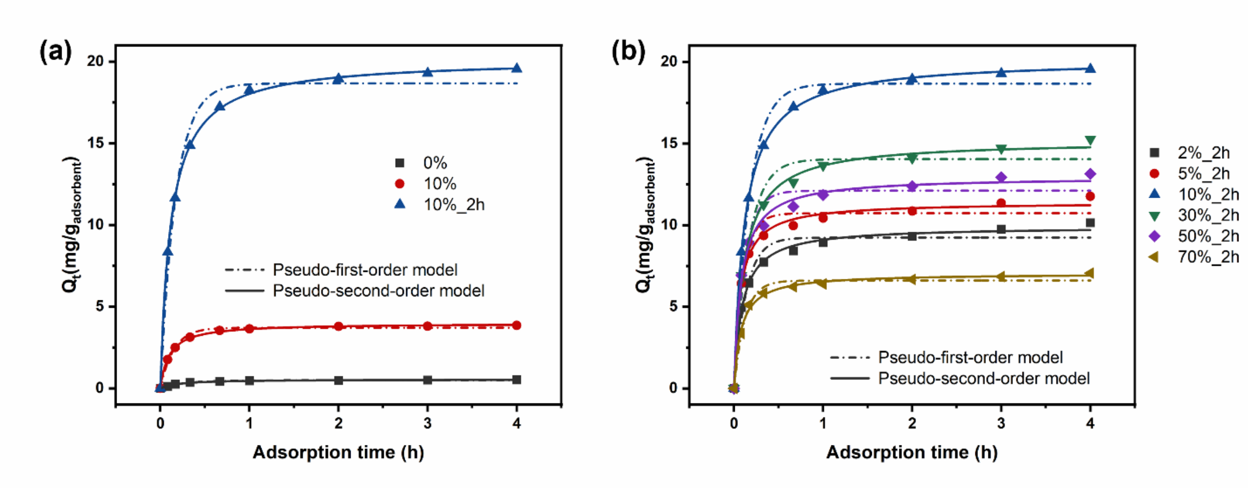

Figure S35. Kinetic curves of Methylene blue for 0%NH_2-ZIF (ZIF-8), 10%NH_2-ZIF and 10%NH_2-ZIF-2h (a), and various ratios of ligands 2-70% removal of R%NH_2-ZIF-2h (b). Kinetic curves are fixed by Pseudo-first-order and Pseudo-second-order models (**Equations S1-S4**).

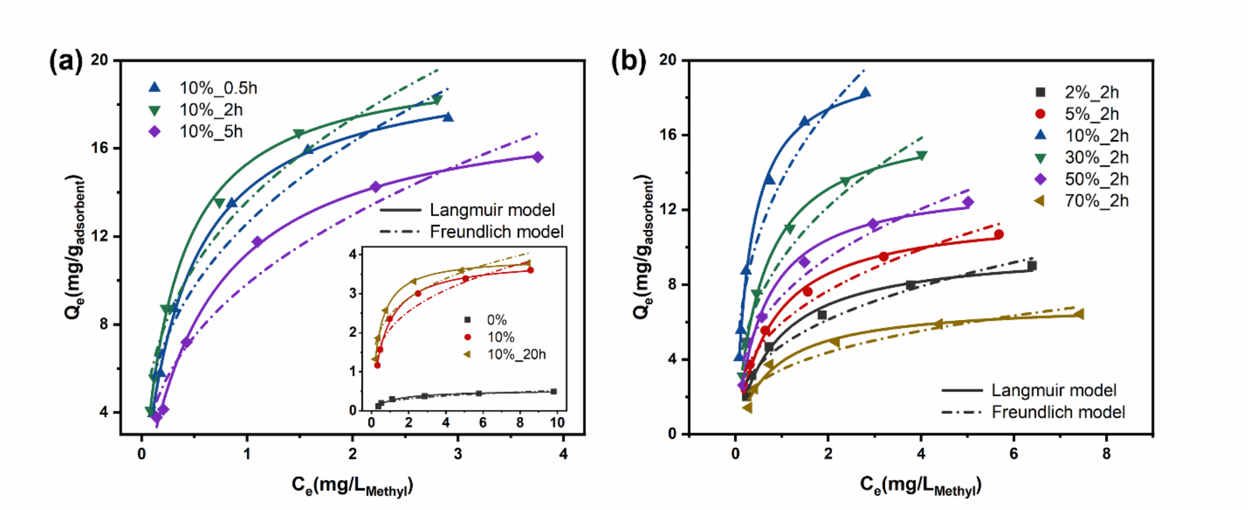

Figure S36. Isotherm curves of MB for 10%NH_2-ZIF-0.5h, 10%NH_2-ZIF-2h and 10%NH_2-ZIF-5h (a). 0%NH_2-ZIF (ZIF-8), 10%NH_2-ZIF-0h and 10%NH_2-ZIF-20h are shown in (a) inset. Various ratios of ligands 2-70% removal of R%NH_2-ZIF-2h (b). Isotherm curves are fixed by Langmuir and Freundlich models (**Equations S5** and **S6**).

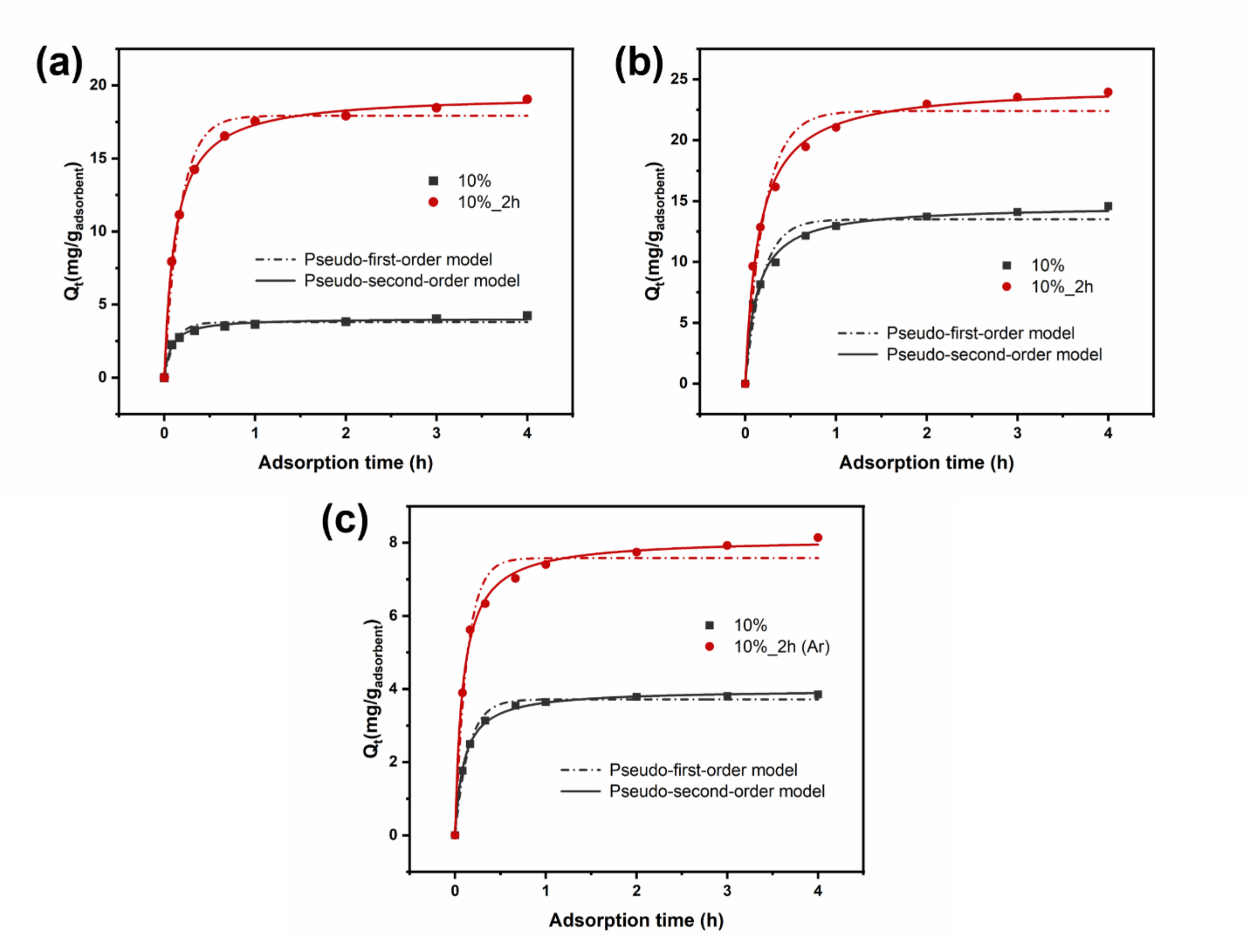

Figure S37. Kinetic curves of methyl orange (a) and rhodamine B (b) for 10%NH_2-ZIF and 10%NH_2-ZIF-2h, the latter being heated 290°C, 2 hours in air atmosphere. Kinetic curves of methylene blue (c) for 10%NH_2-ZIF and 10%NH_2-ZIF-2h (Ar), the latter being heated 500°C, 2 hours in an inert gas of Ar. Kinetic curves are fixed by Pseudo-first-order and Pseudo-second-order models (**Equations S1-S4**).

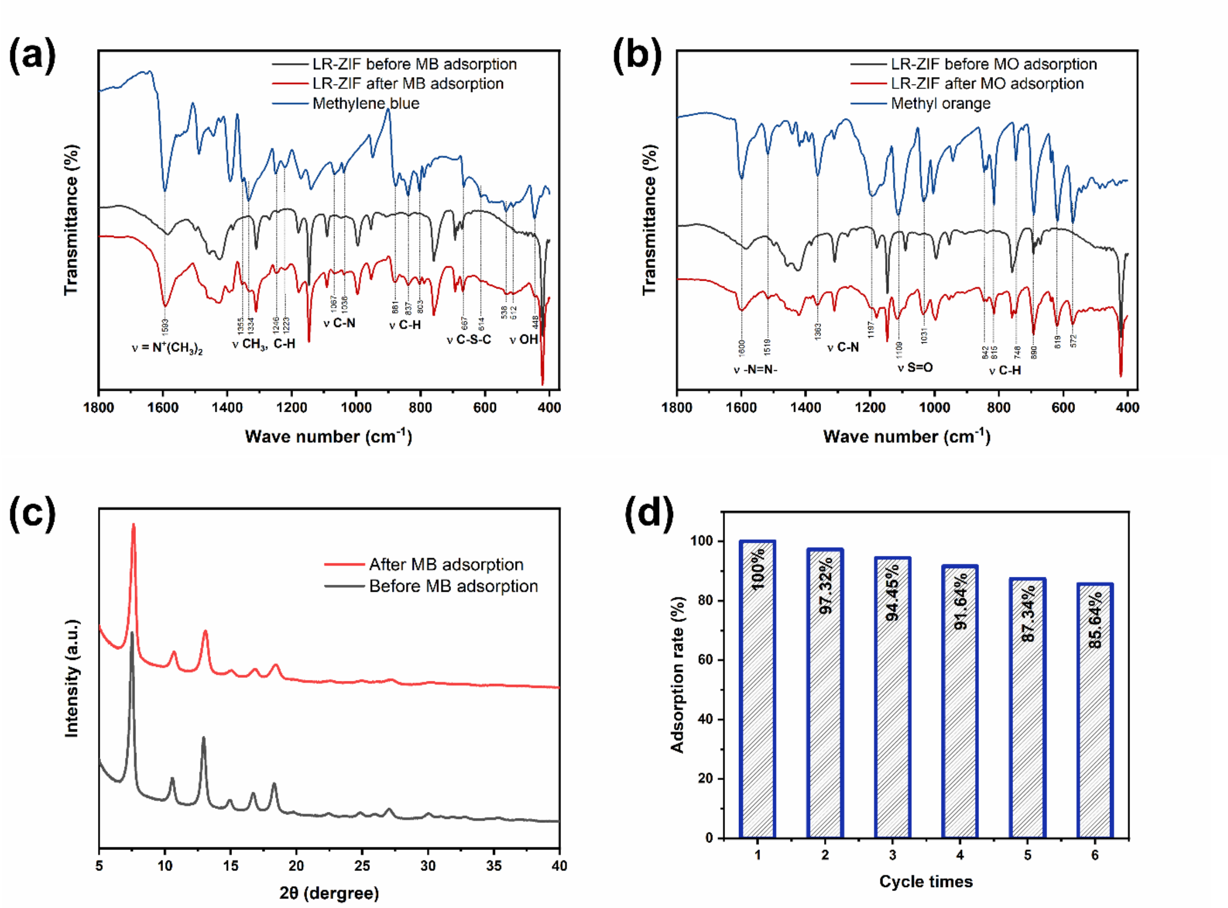

Figure S38. ATR-IR spectra of 10%NH_2-ZIF-2h before and after saturation of methylene blue (a) and methyl orange (b) adsorption. XRD patterns of 10%NH_2-ZIF-2h before and after saturation of methylene blue adsorption (c). Adsorption-desorption cycles with 10%NH_2-ZIF-2h (d), the adsorption of methylene blue reached saturation in each cycle. The slight decrease in adsorption rate after recovery is due to acid washing [19, 20].

Table S4. Kinetics parameters of ZIF-8, ML-ZIF-8 and LR-ZIF-8.

Adsorbents[a]	$Q_{e.exp}$ /mg g^{-1}	Pseudo-first-order model			Pseudo-second-order model		
		$Q_{e.cal}$ /mg g^{-1}	K_1 /h^{-1}	R^2	$Q_{e.cal}$ /mg g^{-1}	K_2 / g mg^{-1} h^{-1}	R^2
0%NH_2-ZIF (ZIF-8)	0.524	0.495	3.694	0.9867	0.551	8.678	0.9919
2%NH_2-ZIF-2h	10.146	9.238	7.473	0.9614	9.922	1.116	0.9942
5%NH_2-ZIF-2h	11.765	10.731	9.402	0.9643	11.413	1.293	0.9932
10%NH_2-ZIF-0h	3.856	3.716	6.717	0.9901	3.983	2.549	0.9985
10%NH_2-ZIF-2h	19.547	18.665	5.788	0.9831	20.166	0.418	0.9996
30%NH_2-ZIF-2h	15.263	14.046	6.132	0.9639	15.183	0.585	0.9958
50%NH_2-ZIF-2h	13.146	12.111	8.269	0.9551	12.962	0.964	0.9928
70%NH_2-ZIF-2h[a]	7.062	6.609	8.345	0.9837	7.048	1.819	0.9929
10%NH_2-ZIF-2h (Ar)	8.141	7.582	7.727	0.9729	8.124	1.424	0.9965
10%NH_2-ZIF-0h (MO[b])	4.226	3.795	8.536	0.9525	4.055	3.217	0.9897
10%NH_2-ZIF-2h (MO[b])	19.053	17.925	5.724	0.9819	13.385	0.428	0.9989
10%NH_2-ZIF-0h (RhB[c])	14.584	13.495	5.443	0.9541	14.635	0.538	0.9926
10%NH_2-ZIF-2h (RhB[c])	23.955	22.402	4.738	0.9601	24.483	0.271	0.9958

[a] In the air atmosphere range, 2-50% heated at 290°C and 70% heated at 260°C. In the Ar flow, 10%NH_2-ZIF heated to 500°C.

[b] Adsorption Kinetics of Methyl Orange in 20 mg L^{-1}.

[c] Adsorption Kinetics of Rhodamine B in 10 mg L^{-1}.

Table S5. Isotherm parameters of ZIF-8, ML-ZIF-8 and LR-ZIF-8.

Adsorbents[a]	Langmuir model			Freundlich model		
	$Q_{m.cal}$ /mg g^{-1}	K_L /L mg^{-1}	R^2	n	K_F / (mg g^{-1}) (mg L^{-1})$^{-n}$	R^2
0%NH_2-ZIF (ZIF-8)	0.531	0.965	0.9801	3.043	0.243	0.9158
2%NH_2-ZIF-2h	9.943	1.151	0.9872	2.717	4.753	0.9596
5%NH_2-ZIF-2h	11.905	1.274	0.9898	2.697	5.903	0.9613
10%NH_2-ZIF-0h	3.874	1.451	0.9961	3.546	2.093	0.9066
10%NH_2-ZIF-0.5h	20.673	2.398	0.9986	2.691	12.589	0.9264
10%NH_2-ZIF-2h	20.835	3.159	0.9969	2.828	13.582	0.9411
10%NH_2-ZIF-5h	18.315	1.573	0.9966	2.508	9.843	0.9448
10%NH_2-ZIF-20h	3.931	2.435	0.9993	4.291	2.454	0.8981
30%NH_2-ZIF-2h	17.001	1.691	0.9958	2.634	9.358	0.9466
50%NH_2-ZIF-2h	13.584	1.647	0.9763	2.834	7.391	0.9481
70%NH_2-ZIF-2h[a]	7.043	1.228	0.9781	2.967	3.467	0.9074

[a] In the air atmosphere range, 2-50% heated at 290°C and 70% heated at 260°C. In the Ar flow, 10%NH_2-ZIF heated to 500°C.

S7. Materials Synthesis and Experimental Section

ZIF-8 was synthesized directly by mixing the ligands 2-mIm and zinc nitrate ($Zn(NO_3)_2·6H_2O$) at room temperature in methanolic solution, as previously reported [21]. 2 g of 2-methylimidazole was dissolved in 30 ml of HPLC methanol, while 0.87 g of zinc nitrate was dissolved in 20 ml of HPLC methanol. The two solutions were then mixed, sonicated for 10 minutes and continuously stirred for 2 hours to accelerate the growth of ZIF-8 crystals. After being allowed to deposit for 24 hours, the ZIF-8 powder was centrifuged, washed three times with methanol and DI water, and then dried in an oven overnight, resulting in a white ZIF-8 powder.

Mixed-ligand ZIF-8 (ML-ZIF-8 or R%NH_2-ZIF) was synthesized via a solvent-assisted ligand exchange (SALE) method. NH_2-bIm (0.04 g, 0.1 g, 0.2 g, 0.6 g, 1 g and 1.4 g) and 2-mIm (1.96 g, 1.9 g, 1.8 g, 1.4 g, 1 g and 0.6 g) were mixed into 30 ml HPLC methanol at various weight ratios (2%, 5%, 10%, 30%, 50% and 70%). The solution was sonicated for 10 minutes and stirred for 30 minutes to ensure even mixing and dissolution of the ligands. The following synthesis steps were the same as for the pure ZIF-8 synthesis, mixing the methanolic solution of the two ligands with zinc nitrate solution (0.87g $Zn(NO_3)_2·6H_2O$; 20 ml HPLC methanol) and stirring for 2 hours. After 24 hours of deposition to allow the crystals formation, the resulting powder was centrifuged and washed three times with methanol and DI water to remove excess ligands. Finally, the obtained NH_2-ZIF powder (R%NH_2-ZIF, where R% indicates the mixing ratio of various NH_2-bIM) was dried overnight, resulting in a white powder.

Ligand-removed ZIF-8 (LR-ZIF-8 or R%NH_2-ZIF-*time*/*temp*), refers to the ZIF-8 samples obtained by thermolysis of ML-ZIF-8 at specific temperatures for specific time periods. The heating process was conducted using either a muffle furnace (LT 5/12 Nabertherm, Germany) in air atmosphere, ramp rate of 10°C/min. Or a tube furnace (HTM Reetz LK-1100, Germany) with argon/nitrogen flow at a ramp rate of 10°C/min. The final temperature was allowed to cool naturally to room temperature. For air atmosphere, the calcination temperature is 290°C for 0-50%NH_2-ZIFs and 260°C for 70%. For argon/nitrogen flow, the calcination temperature was 500°C for 30%NH_2-ZIF and 400°C for 70%NH_2-

ZIF. The samples were labeled as R%NH_2-ZIF-*time*/*temp* (or R%-*time*/*temp*), where R% indicates the mixing ratio of various NH_2-bIM, *time* is the calcination time, and *temp* indicates the special calcination temperature.

Mixed-ligand ZIF-67. NH_2-bIM (0 g, 0.2 g, 0.4 g, 0.6 g, 0.8 g and 1 g) and 2-IM (2 g, 1.8 g, 1.6 g, 1.4 g, 1.2 g and 1 g) were mixed into 30 ml HPLC methanol at various weight ratios (0%, 10%, 20%, 30%, 40% and 50%). Using solvent-assisted ligand exchange (SALE), the synthesis was similar to that of ML-ZIF-8, except for the 0.87 g zinc nitrate ($Zn(NO_3)_2 \cdot 6H_2O$) was replaced by 0.87 g cobalt nitrate ($Co(NO_3)_2 \cdot 6H_2O$). Finally, Purple NH_2-ZIF67 powder (R%NH_2-ZIF67, R% indicates the mixing ratio of various 2-aminobenzimidazole) was obtained.

X-ray Diffraction (XRD). Used PANalytical X'Pert Pro multi-purpose diffractometer (MPD) with Bragg Brentano geometry with Cu anode at 45 kV, 40 mA, equipped with a BBHD Mirror and an X-Celerator multichannel detector. The diffraction patterns were tested at a 2θ angle of 5° and 90°. All measurements were performed with Cu sealed tube Kα and Kß radiation (2:1 ratio) with a wavelength of λ=1.54060 Å at a scan rate of 0.5° min^{-1}. Sample holder is a single crystal silicon on which the sample is immobilized by use of a drop of heptane, and rotated every 4 seconds during the test.

In-situ X-ray Diffraction (In-situ XRD). Same MPD system is used with the addition of the Anton Paar HTK 1200 temperature control system, with heating rate of 5°C/min. The sample was heated with a flow rate of 0.5 mL/min of air, argon and nitrogen.

Attenuated total reflection-Infrared spectroscopy (ATR-IR). Spectra obtained with Perkinelmer Spectrum two FT-IR spectrometer were in the infrared range of 400-4000 cm, with the $LiTaO_3$ (lithium tantalate) MIR detector. Control each data Force Gauge at around 120 with accumulation number of scans is 8 times.

X-ray photoelectron spectroscopy (XPS). Determination of the chemical states of the ALD-synthesized MOF-like material was accomplished by a custom designed XPS, provided by SPECS, using monochromatic AL-Kα (1486,6 eV) radiation an a hemispheric SCIENTA RS4000 photoelectron energy analyzer.

N_2 Physisorption. Measurements were conducted at temperature of 77 K, on a 3Flex instrument by

Micromeritics. Before measuring, the samples were vacuum outgassed at 150°C for 3-12 hours. The apparent surface area was calculated by applying the Brunauer-Emmet-Teller (BET) equation, following the recommended procedure for microporous sorbents [22]. The relevant pore size distributions were determined from the adsorption branch of the isotherms using the kernel of nonlocal density functional theory (NLDFT).

Scanning electron microscope (SEM). Images were recorded on a FEI Quanta 250 (Schottky-)FEG-SEM, facilitated by USTEM (university service center for transmission electron microscopy) at TU Wien, equipped with an ETD Secondary electron detector and an EDAX-AMETEK Octane Elite 55 detector for elemental characterization by energy-dispersive X-ray-spectroscopy. The device was operated at a voltage of 10 kV at a working distance of about 5 mm, providing a maximum resolution of roughly 2 nm. Chemical X-ray microanalysis was performed at 20 kV and 10 mm working distance.

Transmission electron microscope (TEM). All HR-TEM measurements displayed in this paper were performed on a Tecnai F20 FEG-TEM, facilitated by USTEM (university service center for transmission electron microscopy) at TU Wien, equipped with a X-FEG, a Gatan Rio16 CCD-camera, Gatan DigiSTEM II with HAADF detector for STEM imaging, an EDAX-AMETEK Apollo XLTW SDD EDX-detector. The operating voltage was kept at 200 kV for all measurements, providing a maximum lattice resolution of approximately 0.14 nm.

Raman spectroscopy. Measurements were carried out using the WITec alpha 300 RSA^+, focusing on the range 1000-2800 cm^{-1}. Excitation laser wavelength is 532nm, the laser power is 0.1 mA (very low), the integration time is 1 second and the accumulation number is 10.

UV-Vis absorption spectroscopy and diffuse reflectance spectroscopy (UV-Vis and DRS). Supernatant concentrations of methylene blue after adsorption were obtained at 660-670nm by Jasco V-670. Measurements were carried out using the Jasco V-670 in diffuse reflectance mode with an Ulbricht-sphere. After centrifugation of the methylene blue solution, 1 ml×3 of supernatant was added to a cuvette in the UV-Vis incident light range of 200-800 nm for absorbance measurements and the results were averaged over 3 times for all tests. The concentration of the current MB solution was derived from the peak intensity at 667 nm, MO is 464 nm and RhB is 554 nm. In the powders DRS

testing, $MgSO_4$ was used as a baseline, in the range of 200-800 nm. Scan speed is 200 nm/min, data interval is 1 nm for all measurements. All tests were conducted in triplicate, and only the average values are reported here.

Nuclear magnetic resonance spectroscopy (^{1}H NMR). Liquid phase ^{1}H spectra were measured using the Bruker ADVANCE 250 (250.13 MHz) instrument, which is equipped with a 5 mm inverse-broad probe head and z-gradient unit. To serve as an internal reference, Acetic Acid with a δ = 1.96 ppm was used. Due to the limited solubility of MOFs in typical NMR solvents, the sample weighing 2 mg was digested with 0.5 ml of d_4-acetic acid and then sonicated until it was well dispersed in the acid.

Thermogravimetric analysis (TGA). Measurements were carried out using PerkinElmer 8000 (Waltham, USA) with the sample heated in of Al_2O_3 crucible. The heating rate was 10°C/min, either in air, argon and nitrogen flow, and finally held at 600°C for 1 hour.

Density-functional theory (DFT). All of the calculations were performed by using Vienna ab initio program package (VASP) [23]. The exchange and correlation effects of the electrons were described by using the Perdew-Burke-Ernzerhof (PBE) [24] functional of a generalized gradient approximation (GGA) method [25]. The projector augmented wave (PAW) method was used to describe the electron-ion interaction. Spin polarization considered. The cutoff energy of the plane-wave was set as 400 eV. The (2×2×2) k-point mesh was used for k-space integration in our structure relaxations. Conjugate-gradient algorithm is used to relax the ions into their instantaneous ground state. The structure involved were fully relaxed with the energy and force convergences less than 1×10^{-7} eV and 0.03 eV Å^{-1}, respectively.

Adsorption kinetics. Prior to the adsorption experiments, all ZIF adsorbents were dried at 60°C overnight. For the adsorption experiments, 20 mg of adsorbent was added into 50 ml of 10 mg L^{-1} methylene blue solution (20 mg L^{-1} for Methyl orange and 10 mg L^{-1} for Rhodamine B) with deionized (DI) water, and stirred for a suitable period. At regular intervals (ranging from 5 minutes to 4 hours), 1 ml of methylene blue solution was taken out for centrifugation, and the absorbance of the supernatant was measured in the 400-800 nm range. The adsorption capacity of the adsorbent can be calculated by **Equation S1-S2**:

$$Q_t = \frac{(C_i - C_t) \times V}{M} \quad \text{(S1)}$$

$$Q_e = \frac{(C_i - C_e) \times V}{M} \tag{S2}$$

where Q_t (mg g^{-1}) is the adsorption capacity for time t. C_i and C_t are the initial and time t concentrations (mg L^{-1}) of MB solution, can be obtained by UV-vis testing the absorbance of the solution. Q_e and C_e is the adsorption capacity and concentrations for MB equilibrium. V, M represent the volume of the MB solution (L) and the weight of the ZIFs (g) respectively.

The resulting adsorption capacity curves for various time periods can be fitted by Pseudo-first-order (PFO) and Pseudo-second-order (PSO) kinetic models [26], as described by the following **Equation S3-S4**:

$$Q_t = Q_e(1 - e^{-K_1 t}) \tag{S3}$$

$$Q_t = \frac{K_2 Q_e^2 t}{1 + K_2 Q_e t} \tag{S4}$$

K_1 (min^{-1}) and K_2 (g mg^{-1} min^{-1}) are the rate constant for the Pseudo-first-order and Pseudo-second-order model, respectively.

Adsorption isotherms. Prior to the adsorption experiments, all ZIF adsorbents were dried at 60°C overnight. 20 mg of adsorbent was added to the MB solution at various concentrations (1-10 mg L^{-1}). After 2 hours of continuous stirring, the adsorbent was considered to be full saturated (as known by adsorption kinetics). Similarly, C_e and Q_e were calculated from the absorbance data obtained by UV-vis, and the adsorption isotherms were plotted. Langmuir and Freundlich isotherm models [27] were used to fit with the adsorption data at various MB concentrations. Langmuir model represents monolayer molecular adsorption on homogeneous surface and Freundlich model is used for multilayer adsorption on heterogeneous surfaces [18], as described by the following **Equation S4-S5**:

$$Q_e = \frac{Q_{max} K_L C_e}{1 + K_L C_e} \tag{S5}$$

$$Q_e = K_F {C_e}^{1/n} \tag{S6}$$

where Q_{max} (mg g^{-1}) is the maximum adsorption capacity, K_L (L mg^{-1}) and K_F (mg g^{-1}) (mg L^{-1})$^{-n}$ are the Langmuir and Freundlich isotherm constants, respectively, related to the adsorption capacity and adsorbate-adsorbent interactions. n is the adsorption intensity, related to the energy distribution and heterogeneity of the adsorption sites [20].

Adsorption-desorption cycles. After the adsorption experiment, the MB solution was centrifuged to obtain the saturated adsorbed ZIF adsorbent. The adsorbent was then sonicated in HPLC methanol solution for 2 hours, followed by centrifugation and rinsing with 0.1 mol L^{-1} H_2SO_4 and DI water. This desorption process was repeated three times to obtain adsorbent that could be reused for subsequent adsorption experiments.

S8. References

[1] R. J. Sundberg, R. B. Martin, Chem. Rev. 1974, 74, 471.
[2] K. Eum, K. C. Jayachandrababu, F. Rashidi, K. Zhang, J. Leisen, S. Graham, R. P. Lively, R. R. Chance, D. S. Sholl, C. W. Jones, S. Nair, J. Am. Chem. Soc. 2015, 137, 4191.
[3] L. Feng, S. Yuan, L.-L. Zhang, K. Tan, J.-L. Li, A. Kirchon, L.-M. Liu, P. Zhang, Y. Han, Y. J. Chabal, H.-C. Zhou, J. Am. Chem. Soc. 2018, 140, 2363.
[4] J. B. James, Y. S. Lin, J. Phys. Chem. C 2016, 120, 14015.
[5] J. A. Thompson, J. T. Vaughn, N. A. Brunelli, W. J. Koros, C. W. Jones, S. Nair, Microporous Mesoporous Mater. 2014, 192, 43.
[6] A. Phan, C. J. Doonan, F. J. Uribe-Romo, C. B. Knobler, M. O'Keeffe, O. M. Yaghi, Acc. Chem. Res. 2010, 43, 58.
[7] C. Wu, D. Xie, Y. Mei, Z. Xiu, K. M. Poduska, D. Li, B. Xu, D. Sun, Phys. Chem. Chem. Phys. 2019, 21, 17571.
[8] Y. Zhang, N. Zhang, Y. Liu, Y. Chen, H. Huang, W. Wang, X. Xu, Y. Li, F. Fan, J. Ye, Z. Li, Z. Zou, Nat. Commun. 2022, 13, 2942.
[9] C. Schlumberger, M. Thommes, Adv. Mater. Interfaces 2021, 8, 2002181.
[10] D. Tuncel, A. N. Ökte, Catal. Today 2021, 361, 191.
[11] L. Oliviero, J. Barbier, D. Duprez, Appl. Catal., B 2003, 40, 163.
[12] H. Chen, K. Shen, Q. Mao, J. Chen, Y. Li, ACS Catalysis 2018, 8, 1417.
[13] W. Zhang, X. Jiang, Y. Zhao, A. Carné-Sánchez, V. Malgras, J. Kim, J. H. Kim, S. Wang, J. Liu, J.-S. Jiang, Y. Yamauchi, M. Hu, Chem. Sci. 2017, 8, 3538.
[14] Y. Pan, K. Sun, S. Liu, X. Cao, K. Wu, W.-C. Cheong, Z. Chen, Y. Wang, Y. Li, Y. Liu, D. Wang, Q. Peng, C. Chen, Y. Li, J. Am. Chem. Soc. 2018, 140, 2610.
[15] R. Jiang, T. Liu, R. Wu, C. Guo, Y. Chen, G. Xiang, L. Wang, ACS Appl. Mater. Interfaces 2020, 12, 7270.
[16] J. Tang, R. R. Salunkhe, J. Liu, N. L. Torad, M. Imura, S. Furukawa, Y. Yamauchi, J. Am. Chem. Soc. 2015, 137, 1572.
[17] A. Hu, X. Lu, D. Cai, H. Pan, R. Jing, Q. Xia, D. Zhou, Y. Xia, Molecular Catalysis 2019, 472, 27.
[18] R. Kaur, A. Kaur, A. Umar, W. A. Anderson, S. K. Kansal, Mater. Res. Bull. 2019, 109, 124.
[19] G. Wang, J. Zhang, S. Lin, H. Xiao, Q. Yang, S. Chen, B. Yan, Y. Gu, Cellulose 2020, 27, 2085.
[20] Y. R. Lee, X. H. Do, K. Y. Cho, K. Jeong, K.-Y. Baek, ACS Applied Nano Materials 2020, 3, 9852.
[21] Z. Huang, Q. Zhou, J. Wang, Y. Yu, J. Mater. Sci. Technol. 2022, 112, 68.
[22] F. O. Sánchez-Varretti, G. D. García, A. J. Ramirez-Pastor, F. Romá, J. Chem. Phys. 2009, 130, 194711.
[23] Kresse, Furthmuller, Phys. Rev. B: Condens. Matter 1996, 54, 11169.
[24] J. P. Perdew, A. Ruzsinszky, G. I. Csonka, O. A. Vydrov, G. E. Scuseria, L. A. Constantin, X. L. Zhou, K. Burke, Phys. Rev. Lett. 2008, 100, 4.
[25] J. P. Perdew, K. Burke, M. Ernzerhof, Phys. Rev. Lett. 1996, 77, 3865.

[26] J.-P. Simonin, Chem. Eng. J. 2016, 300, 254.
[27] K. Y. Foo, B. H. Hameed, Chem. Eng. J. 2010, 156, 2.
