# Supplementary material for: Hierarchically Micro‐ and Mesoporous Zeolitic Imidazolate Frameworks Through Selective Ligand Removal
Source: Small. 2023 Dec 21;20(21):2307981. doi: 10.1002/smll.202307981 (PMC11478943; doi:10.1002/smll.202307981)
Supplement: Supplementary file 2 — Supporting Information [file SMLL-20-2307981-s002.zip › Huang_DFT_Support.pdf]

DFT Supporting Information

Hierarchically Micro- and Mesoporous Zeolitic Imidazolate Frameworks through Selective Ligand Removal

Zheao Huang[1], Jakob Rath[1], Qiancheng Zhou[2], Alexey Cherevan[1], Shaghayegh Naghdi[1], and Dominik Eder[,1]*

[1] Institute of Material Chemistry, Vienna University of Technology, 1060, Vienna, Austria

[2] Institute of Nanoscience and Nanotechnology, College of Physical Science and Technology, Central China Normal University, 430079, Wuhan, China

* Corresponding author. Email: dominik.eder@tuwien.ac.at.

DFT calculation details and parameters:

Remove one ligand

```
1.0
      16.99560000         0.00000000         0.00000000
       0.00000000        16.99560000         0.00000000
       0.00000000         0.00000000        16.99560000
   H    C    Zn    N
   100    80    11    40
Direct
   0.2699431858168801    0.1505008899006274    0.3851379725874769
   0.3506274177342137    0.5732390039637105    0.1214097476480563
   0.7770419661490542    0.6511674509289737    0.8952250026657629
   0.6441589571361701    0.8813274830410048    0.5687022798718895
   0.8488325490710263    0.2770419661490546    0.6047749973342371
   0.7346220315764267    0.6200177137108085    0.1511965881699951
   0.2288738705066736    0.1165640817592976    0.6483171090957218
   0.9267609960362895    0.8506274177342141    0.3785902523519438
   0.8562619825710093    0.9286381991508512    0.3789395217113168
   0.3541464222990489    0.4317493538046848    0.8847501460174280
   0.8814735066930224    0.6378817930699446    0.5628250941500087
   0.2690358479999571    0.8501607881225028    0.6150387989579903
   0.3817253145865555    0.1434343186059839    0.0689754355685640
   0.1169066687707204    0.2348667365599836    0.6460646157017976
   0.9317493538046847    0.1458535777009509    0.6152498539825720
   0.3494991100993725    0.7699431858168807    0.1148620274125231
   0.0682506461953151    0.8541464222990491    0.6152498539825720
   0.4301001886521564    0.3597187930363408    0.8841286080765667
   0.5713618008491488    0.3562619825710093    0.1210604782886832
   0.6498392118774972    0.7690358479999567    0.8849612010420097
   0.1201069469385233    0.4333433470418791    0.6399605411611295
   0.6493725822657859    0.4267609960362891    0.1214097476480563
   0.9333433470418792    0.3798930530614767    0.8600394588388705
   0.5698998113478437    0.6402812069636596    0.8841286080765667
   0.0666566529581209    0.6201069469385232    0.8600394588388705
   0.4306951918353639    0.8815946260634956    0.3568786302871435
   0.3498867275250054    0.1149381051191506    0.7704725554850865
   0.6501132724749953    0.8850618948808495    0.7704725554850865
   0.8597187930363404    0.0698998113478435    0.6158713919234333
   0.6163484127274026    0.7324174894902026    0.1509801753277846
   0.8799822862891915    0.2346220315764264    0.3488034118300050
```

0.6186725169589952	0.1441589571361706	0.9312977201281105
0.3834359182407022	0.7288738705066740	0.8516828909042782
0.6505008899006277	0.2300568141831193	0.1148620274125231
0.0732390039637106	0.1493725822657862	0.3785902523519437
0.1511674509289739	0.7229580338509458	0.6047749973342371
0.4286381991508512	0.6437380174289907	0.1210604782886832
0.3501607881225032	0.2309641520000431	0.8849612010420097
0.6184053739365044	0.9306951918353641	0.1431213697128566
0.3854953723302801	0.1504554678572540	0.2702004261774685
0.6189351224006157	0.0700032743619083	0.8571151677349993
0.8856702639176306	0.6488787624626142	0.7734417455571647
0.3850618948808497	0.8498867275250047	0.7295274445149135
0.5693048081646359	0.1184053739365045	0.3568786302871435
0.8511212375373858	0.3856702639176308	0.7265582544428353
0.3495445321427461	0.8854953723302795	0.2297995738225317
0.4299967256380916	0.1189351224006160	0.6428848322650007
0.8830933312292799	0.7651332634400165	0.6460646157017976
0.6145046276697205	0.8495445321427462	0.2702004261774685
0.1200177137108085	0.7653779684235733	0.3488034118300050
0.1143297360823692	0.3511212375373857	0.7734417455571647
0.3810648775993840	0.9299967256380921	0.8571151677349993
0.6182746854134443	0.8565656813940155	0.0689754355685640
0.8621182069300554	0.3814735066930227	0.9371749058499913
0.8798930530614768	0.5666566529581208	0.6399605411611295
0.2651332634400165	0.6169066687707201	0.8539353842982024
0.3815946260634957	0.0693048081646361	0.1431213697128566
0.6149381051191505	0.1501132724749952	0.7295274445149135
0.5700032743619079	0.8810648775993843	0.6428848322650007
0.6165640817592980	0.2711261294933267	0.8516828909042782
0.1488787624626141	0.6143297360823694	0.7265582544428353
0.6504554678572538	0.1145046276697200	0.2297995738225317
0.3813274830410054	0.8558410428638298	0.9312977201281105
0.1402812069636593	0.9301001886521562	0.6158713919234333
0.5494812787313401	0.0777510635544483	0.4507198059456664
0.5503294794290682	0.9220699537973315	0.5491294940168936
0.1185264933069773	0.3621182069300554	0.5628250941500087
0.4475169679987431	0.4537513410275050	0.9255340179318041
0.0462486589724948	0.9475169679987435	0.5744659820681959
0.5555777939033958	0.4522670793372093	0.0834870973981987
0.9537513410275049	0.0524830320012567	0.5744659820681959
0.4444222060966042	0.5477329206627908	0.0834870973981987

0.0477329206627908	0.0555777939033957	0.4165129026018013
0.5524830320012565	0.5462486589724951	0.9255340179318041
0.5777751063554477	0.9505187212686599	0.0492801940543336
0.5779300462026685	0.0503294794290683	0.9508705059831064
0.4220699537973309	0.9496705205709318	0.9508705059831064
0.6458535777009509	0.5682506461953153	0.8847501460174280
0.9570865931024233	0.4215732773703403	0.9527168392744904
0.3836515872725972	0.2675825105097979	0.1509801753277846
0.4222248936445518	0.0494812787313405	0.0492801940543336
0.0784267226296594	0.4570865931024233	0.5472831607255096
0.4505187212686593	0.9222248936445523	0.4507198059456664
0.3565656813940165	0.8817253145865557	0.4310245644314362
0.7675825105097974	0.1163484127274029	0.3490198246722154
0.2653779684235736	0.3799822862891914	0.1511965881699951
0.7711261294933260	0.8834359182407021	0.6483171090957218
0.3558410428638293	0.1186725169589948	0.5687022798718895
0.0429134068975766	0.5784267226296592	0.9527168392744904
0.7309641520000433	0.1498392118774967	0.6150387989579903
0.1437380174289906	0.0713618008491489	0.3789395217113168
0.2229580338509458	0.3488325490710261	0.8952250026657629
0.7300568141831193	0.8494991100993723	0.3851379725874769
0.6434343186059845	0.1182746854134443	0.4310245644314362
0.2324174894902021	0.8836515872725974	0.3490198246722154
0.9522670793372092	0.9444222060966042	0.4165129026018013
0.1378817930699445	0.6185264933069776	0.9371749058499913
0.7348667365599835	0.3830933312292799	0.8539353842982024
0.9215732773703408	0.5429134068975767	0.5472831607255096
0.4496705205709316	0.0779300462026688	0.5491294940168936
0.5056149718280580	0.8768731133212652	0.1229912344916970
0.3099724115347661	0.3670144512256199	0.1073412037708740
0.8136039946009893	0.8680122450264206	0.6031963097689009
0.8112349876568657	0.1315737906584953	0.3930608324581866
0.1815097419698025	0.3941477120823064	0.8777170521241602
0.6878323436324330	0.1051536708383690	0.6299424218205468
0.6303036620320248	0.1052884785869230	0.6867070508596509
0.3092773854156655	0.6309988322168544	0.8978677879400400
0.5844698645295731	0.0959450679880913	0.9052250720335141
0.5061672915103657	0.1239741618921066	0.8769474385537159
0.8941477120823068	0.3184902580301974	0.6222829478758398
0.1863960053990105	0.1319877549735799	0.6031963097689009
0.6231268866787348	0.0056149718280579	0.3770087655083029

0.3755973267003992	0.3774017647704046	0.9969791397105268
0.3768731133212653	0.9943850281719420	0.3770087655083029
0.1887650123431336	0.8684262093415046	0.3930608324581866
0.0903187834823443	0.5850524288617909	0.9089056076870267
0.1189885882844389	0.5068333127148988	0.8812296322541183
0.5951638369644113	0.0840310460532134	0.4050979866270479
0.6907226145843340	0.3690011677831457	0.8978677879400400
0.6900275884652349	0.6329855487743793	0.1073412037708740
0.9096812165176554	0.4149475711382095	0.9089056076870267
0.8810114117155607	0.4931666872851003	0.8812296322541183
0.1225982352295955	0.8755973267003989	0.5030208602894732
0.0930363538637884	0.9030826439466423	0.5810320036750916
0.6244026732996011	0.6225982352295951	0.9969791397105268
0.5969173560533577	0.5930363538637889	0.9189679963249084
0.8769483685264146	0.8748846689687234	0.4914950479801694
0.6251153310312766	0.3769483685264142	0.0085049520198305
0.5989046729743297	0.4042122380688504	0.0876507551528169
0.6319877549735794	0.3136039946009896	0.8968036902310991
0.1230516314735857	0.1251153310312760	0.4914950479801694
0.0957877619311496	0.0989046729743297	0.4123492448471830
0.6057674358377972	0.1296681863508968	0.1867724778408380
0.6058045814057899	0.1869294877934381	0.1297458864969132
0.1058522879176934	0.6815097419698024	0.6222829478758398
0.4010953270256706	0.5957877619311498	0.0876507551528169
0.9069636461362111	0.0969173560533576	0.5810320036750916
0.8774017647704049	0.1244026732996008	0.5030208602894732
0.3703318136491033	0.1057674358377978	0.3132275221591623
0.3748846689687244	0.6230516314735854	0.0085049520198305
0.5959450679880910	0.9155301354704269	0.5947749279664859
0.6239741618921063	0.9938327084896343	0.6230525614462842
0.4040549320119087	0.0844698645295732	0.5947749279664859
0.3760258381078936	0.0061672915103652	0.6230525614462841
0.8690011677831456	0.8092773854156659	0.6021322120599600
0.6296681863508966	0.8942325641622026	0.3132275221591623
0.3684262093415048	0.3112349876568665	0.1069391675418134
0.1277547091303741	0.3953656797662940	0.8175180253965371
0.4030826439466421	0.4069636461362116	0.9189679963249084
0.1329855487743800	0.8099724115347651	0.3926587962291258
0.6051536708383696	0.8121676563675670	0.8700575781794532
0.3941954185942104	0.8130705122065620	0.1297458864969132
0.6315737906584954	0.6887650123431343	0.1069391675418134

0.4159689539467865	0.0951638369644110	0.0949020133729520
0.3680122450264199	0.6863960053990107	0.8968036902310991
0.8670144512256207	0.1900275884652340	0.3926587962291258
0.8722452908696254	0.6046343202337060	0.8175180253965371
0.3696963379679754	0.8947115214130770	0.6867070508596509
0.3121676563675665	0.8948463291616304	0.6299424218205468
0.3942325641622020	0.8703318136491034	0.1867724778408380
0.1309988322168545	0.1907226145843346	0.6021322120599600
0.1046343202337058	0.6277547091303746	0.6824819746034629
0.9149475711382091	0.5903187834823446	0.5910943923129733
0.9931666872851012	0.6189885882844393	0.6187703677458817
0.4155301354704267	0.9040549320119090	0.9052250720335141
0.4938327084896348	0.8760258381078937	0.8769474385537159
0.0850524288617906	0.4096812165176557	0.5910943923129733
0.0068333127148994	0.3810114117155610	0.6187703677458817
0.5840310460532130	0.9048361630355887	0.0949020133729520
0.9042122380688502	0.9010953270256703	0.4123492448471830
0.8953656797662940	0.3722452908696260	0.6824819746034629
0.3130705122065615	0.1058045814057896	0.3702541135030869
0.3947115214130769	0.1303036620320248	0.8132929491403491
0.8184902580301976	0.6058522879176932	0.8777170521241602
0.6052884785869230	0.8696963379679752	0.8132929491403491
0.6869294877934380	0.8941954185942101	0.3702541135030869
0.3948463291616308	0.1878323436324333	0.8700575781794532
0.4943850281719425	0.1231268866787345	0.1229912344916970
0.4048361630355889	0.9159689539467870	0.4050979866270479
0.7504440704820284	0.5010183259641535	0.0012913686558102
0.2490632938891892	-0.0000005508783677	0.4994272638156895
-0.0010183259641538	0.2504440704820283	0.4987086313441900
0.0010183259641538	0.7495559295179716	0.4987086313441900
0.5000000000000000	0.0000000000000000	0.2500000000000000
0.5000000000000000	0.0000000000000000	0.7500000000000000
0.4999994491216323	0.2509367061108106	0.0005727361843106
-0.0000000000000000	0.5000000000000000	0.7500000000000000
0.2495559295179716	0.4989816740358458	0.0012913686558102
0.5000005508783678	0.7490632938891894	0.0005727361843106
0.7509367061108106	0.0000005508783677	0.4994272638156895
0.9086617798875201	0.1853351375470721	0.4621619122316900
0.4091862904280080	0.6813678515106799	0.9667341833816584
0.8178084729797483	0.0904237957486197	0.4628963857115605
0.5902308874976975	0.0347606151108084	0.6823699927926470

0.3137385666997766	0.5910909145510489	0.9686546104256425
0.5347606151108085	0.9097691125023025	0.8176300072073530
0.4652393848891916	0.0902308874976981	0.8176300072073530
0.5352172745654054	0.0896741834857524	0.1824883810308980
0.0334733920899967	0.5885941589106085	0.6801889591038115
0.9665266079100032	0.4114058410893908	0.6801889591038115
0.4647827254345950	0.9103258165142476	0.1824883810308980
0.5908137095719922	0.3186321484893202	0.9667341833816584
0.0913382201124795	0.8146648624529278	0.4621619122316900
0.9114058410893915	0.5334733920899968	0.8198110408961885
0.3146648624529280	0.4086617798875204	0.0378380877683100
0.4097691125023018	0.9652393848891915	0.6823699927926470
0.8186321484893201	0.9091862904280078	0.5332658166183416
0.0910909145510492	0.1862614333002234	0.5313453895743575
0.3161421266790376	0.9654535960398888	0.5900067383495569
0.5345464039601112	0.8161421266790378	0.9099932616504431
0.4654535960398887	0.1838578733209621	0.9099932616504431
0.4103258165142475	0.0352172745654050	0.3175116189691018
0.5896741834857524	0.9647827254345946	0.3175116189691018
0.5904237957486194	0.6821915270202517	0.0371036142884396
0.0885941589106092	0.4665266079100036	0.8198110408961885
0.5352414291250278	0.1827588462986554	0.0897478818965265
0.0351245336224212	0.6760263462478366	0.5824081995549709
0.9089090854489511	0.8137385666997766	0.5313453895743575
0.4095762042513802	0.3178084729797490	0.0371036142884396
0.1760263462478370	0.4648754663775785	0.9175918004450291
0.6838578733209622	0.0345464039601114	0.5900067383495569
0.6853351375470722	0.5913382201124799	0.0378380877683100
0.6862614333002234	0.4089090854489504	0.9686546104256425
0.1821915270202511	0.9095762042513806	0.4628963857115605
0.6827588462986555	0.9647585708749722	0.4102521181034732
0.3172411537013446	0.0352414291250273	0.4102521181034732
0.8239736537521634	0.5351245336224214	0.9175918004450291
0.4647585708749728	0.8172411537013445	0.0897478818965265
0.9648754663775786	0.3239736537521630	0.5824081995549709
0.1813678515106798	0.0908137095719920	0.5332658166183416

Remove two ligands

```
   1.00000000000000
    16.9955999999999996    0.0000000000000000    0.0000000000000000
     0.0000000000000000   16.9955999999999996    0.0000000000000000
     0.0000000000000000    0.0000000000000000   16.9955999999999996
   H    C    Zn   N
    80    64    10    32
Direct
  0.2684426207711609  0.1494185292864423  0.3844820966594334
  0.3524914731660427  0.5678092651730658  0.1185839840881922
  0.6434482270511573  0.8812116694008438  0.5682139997473656
  0.7378226250643967  0.6196351107796025  0.1469504842473385
  0.2289921733955959  0.1167227043175458  0.6494863425179689
  0.9321907348269342  0.8524914731660428  0.3814160159118076
  0.8592054930619148  0.9277489278296971  0.3809399472373072
  0.3524914731660427  0.4321907348269338  0.8814160159118082
  0.2684426207711609  0.8505814707135581  0.6155179033405663
  0.3812116694008439  0.1434482270511577  0.0682139997473654
  0.1196351107796022  0.2378226250643970  0.6469504842473386
  0.9321907348269342  0.1475085268339573  0.6185839840881918
  0.3505814707135578  0.7684426207711615  0.1155179033405664
  0.0678092651730662  0.8524914731660428  0.6185839840881918
  0.4277489278296971  0.3592054930619154  0.8809399472373076
  0.5722510721703029  0.3592054930619154  0.1190600527626928
  0.6494185292864419  0.7684426207711615  0.8844820966594337
  0.5722510721703029  0.6407945069380852  0.8809399472373076
  0.4306760221405543  0.8813981619474656  0.3575569073338820
  0.3504546782631890  0.1156692300989782  0.7700163477814380
  0.6495453217368113  0.8843307699010218  0.7700163477814380
  0.8592054930619148  0.0722510721703031  0.6190600527626924
  0.6167227043175456  0.7289921733955961  0.1494863425179687
  0.8803648892203975  0.2378226250643970  0.3530495157526613
  0.6187883305991562  0.1434482270511577  0.9317860002526344
  0.3832772956824542  0.7289921733955961  0.8505136574820311
  0.6494185292864419  0.2315573792288391  0.1155179033405664
  0.0678092651730662  0.1475085268339573  0.3814160159118076
  0.4277489278296971  0.6407945069380852  0.1190600527626928
  0.3505814707135578  0.2315573792288391  0.8844820966594337
  0.6186018380525344  0.9306760221405542  0.1424430926661178
  0.3843307699010217  0.1495453217368112  0.2700163477814385
  0.6186018380525344  0.0693239778594457  0.8575569073338819
```

0.3843307699010217	0.8504546782631887	0.7299836522185620
0.5693239778594458	0.1186018380525343	0.3575569073338820
0.3504546782631890	0.8843307699010218	0.2299836522185614
0.4306760221405543	0.1186018380525343	0.6424430926661181
0.8803648892203975	0.7621773749356033	0.6469504842473386
0.6156692300989782	0.8504546782631887	0.2700163477814385
0.1196351107796022	0.7621773749356033	0.3530495157526613
0.3813981619474660	0.9306760221405542	0.8575569073338819
0.6187883305991562	0.8565517729488427	0.0682139997473654
0.2621773749356029	0.6196351107796025	0.8530495157526614
0.3813981619474660	0.0693239778594457	0.1424430926661178
0.6156692300989782	0.1495453217368112	0.7299836522185620
0.5693239778594458	0.8813981619474656	0.6424430926661181
0.6167227043175456	0.2710078266044042	0.8505136574820311
0.6495453217368113	0.1156692300989782	0.2299836522185614
0.3812116694008439	0.8565517729488427	0.9317860002526344
0.5537569210941946	0.5466826085079248	0.9219479036133488
0.5498726013263479	0.0771935418209737	0.4511565244102347
0.5498726013263479	0.9228064581790263	0.5488434755897651
0.9533173914920752	0.9462430789058054	0.4219479036133483
0.4462430789058053	0.4533173914920749	0.9219479036133488
0.0466826085079251	0.9462430789058054	0.5780520963866512
0.5537569210941946	0.4533173914920749	0.0780520963866515
0.9533173914920752	0.0537569210941945	0.5780520963866513
0.4462430789058053	0.5466826085079248	0.0780520963866515
0.0466826085079251	0.0537569210941945	0.4219479036133483
0.5771935418209737	0.9501273986736523	0.0488434755897652
0.5771935418209737	0.0498726013263475	0.9511565244102349
0.4228064581790261	0.9501273986736521	0.9511565244102349
0.6475085268339572	0.5678092651730658	0.8814160159118082
0.3832772956824542	0.2710078266044042	0.1494863425179688
0.4228064581790261	0.0498726013263475	0.0488434755897652
0.4501273986736526	0.0771935418209737	0.5488434755897651
0.4501273986736526	0.9228064581790263	0.4511565244102347
0.3565517729488422	0.8812116694008438	0.4317860002526345
0.7710078266044039	0.1167227043175458	0.3505136574820314
0.2621773749356029	0.3803648892203976	0.1469504842473385
0.7710078266044039	0.8832772956824544	0.6494863425179689
0.3565517729488422	0.1187883305991560	0.5682139997473656
0.7315573792288385	0.1494185292864423	0.6155179033405663
0.1407945069380847	0.0722510721703031	0.3809399472373071

0.7315573792288385	0.8505814707135581	0.3844820966594334
0.1407945069380847	0.9277489278296971	0.6190600527626924
0.6475085268339572	0.4321907348269338	0.1185839840881922
0.6434482270511572	0.1187883305991560	0.4317860002526345
0.2289921733955959	0.8832772956824544	0.3505136574820314
0.7378226250643967	0.3803648892203976	0.8530495157526614
0.5063872210254380	0.8758436909282963	0.1229485343216629
0.3075219773257620	0.3679360830132507	0.1036597076330788
0.8134477544137013	0.8672230309158103	0.6045643587362480
0.8134477544137013	0.1327769690841898	0.3954356412637512
0.6884632283354667	0.1046695987842801	0.6303275954043842
0.6309141752556824	0.1047790741704795	0.6870576899944176
0.3075219773257620	0.6320639169867487	0.8963402923669214
0.5842985268455277	0.0955091943633014	0.9056396306816856
0.5063872210254380	0.1241563090717039	0.8770514656783368
0.4044908056366986	0.9157014731544721	0.4056396306816855
0.1865522455862988	0.1327769690841898	0.6045643587362480
0.3758436909282959	0.9936127789745620	0.3770514656783371
0.6241563090717037	0.0063872210254375	0.3770514656783371
0.3736524641133313	0.3780964084859927	0.9936340519118106
0.1865522455862988	0.8672230309158103	0.3954356412637512
0.5955091943633017	0.0842985268455277	0.4056396306816855
0.6924780226742379	0.3679360830132507	0.8963402923669214
0.6924780226742379	0.6320639169867487	0.1036597076330788
0.1219035915140072	0.8736524641133316	0.5063659480881894
0.0929936363370855	0.9013089526554334	0.5844904673157211
0.6263475358866685	0.6219035915140069	0.9936340519118106
0.5986910473445666	0.5929936363370858	0.9155095326842788
0.8780964084859931	0.8736524641133315	0.4936340519118105
0.6263475358866685	0.3780964084859927	0.0063659480881893
0.5986910473445666	0.4070063636629146	0.0844904673157216
0.6327769690841897	0.3134477544137012	0.8954356412637520
0.1219035915140072	0.1263475358866687	0.4936340519118105
0.0929936363370855	0.0986910473445666	0.4155095326842785
0.6047790741704795	0.1309141752556822	0.1870576899944175
0.6046695987842804	0.1884632283354666	0.1303275954043840
0.3953304012157199	0.1884632283354666	0.8696724045956158
0.4013089526554335	0.5929936363370858	0.0844904673157216
0.9070063636629142	0.0986910473445666	0.5844904673157212
0.8780964084859931	0.1263475358866687	0.5063659480881894
0.3690858247443177	0.1047790741704795	0.3129423100055827

0.3736524641133313	0.6219035915140069	0.0063659480881893
0.5955091943633017	0.9157014731544721	0.5943603693183144
0.6241563090717037	0.9936127789745620	0.6229485343216632
0.4044908056366986	0.0842985268455277	0.5943603693183144
0.3758436909282959	0.0063872210254375	0.6229485343216632
0.8679360830132513	0.8075219773257621	0.6036597076330786
0.6309141752556825	0.8952209258295205	0.3129423100055827
0.3672230309158102	0.3134477544137012	0.1045643587362486
0.4013089526554335	0.4070063636629146	0.9155095326842788
0.1320639169867489	0.8075219773257621	0.3963402923669211
0.6046695987842804	0.8115367716645333	0.8696724045956158
0.3953304012157199	0.8115367716645333	0.1303275954043840
0.6327769690841897	0.6865522455862987	0.1045643587362486
0.4157014731544721	0.0955091943633014	0.0943603693183144
0.3672230309158102	0.6865522455862987	0.8954356412637520
0.8679360830132513	0.1924780226742382	0.3963402923669211
0.3690858247443177	0.8952209258295205	0.6870576899944176
0.3115367716645335	0.8953304012157196	0.6303275954043842
0.3952209258295206	0.8690858247443176	0.1870576899944174
0.1320639169867489	0.1924780226742382	0.6036597076330786
0.4157014731544721	0.9044908056366983	0.9056396306816856
0.4936127789745624	0.8758436909282963	0.8770514656783368
0.5842985268455277	0.9044908056366983	0.0943603693183144
0.9070063636629142	0.9013089526554334	0.4155095326842785
0.3115367716645335	0.1046695987842801	0.3696724045956159
0.3952209258295206	0.1309141752556822	0.8129423100055824
0.6047790741704795	0.8690858247443175	0.8129423100055824
0.6884632283354667	0.8953304012157196	0.3696724045956159
0.4936127789745624	0.1241563090717039	0.1229485343216629
0.7583892251544154	0.5000000000000000	0.0000000000000000
0.2474454649152446	0.0000000000000000	0.5000000000000000
0.0000000000000000	0.2583892251544153	0.5000000000000000
0.0000000000000000	0.7416107748455846	0.5000000000000000
0.5000000000000000	0.0000000000000000	0.2500000000000000
0.5000000000000000	0.0000000000000000	0.7500000000000000
0.5000000000000000	0.2525545350847554	0.0000000000000000
0.2416107748455846	0.5000000000000000	0.0000000000000000
0.5000000000000000	0.7474454649152440	0.0000000000000000
0.7525545350847560	0.0000000000000000	0.5000000000000000
0.9088123845306457	0.1880199159244439	0.4663880747730142
0.8197186953183979	0.0913542775964999	0.4652036362194025

0.5905226707098327	0.0344453486662317	0.6823978058294609
0.3119800840755561	0.5911876154693543	0.9663880747730139
0.5344453486662315	0.9094773292901673	0.8176021941705391
0.4655546513337683	0.0905226707098327	0.8176021941705391
0.4655546513337683	0.9094773292901673	0.1823978058294611
0.9088123845306457	0.8119800840755560	0.5336119252269861
0.5913542775964998	0.3197186953183980	0.9652036362194026
0.0911876154693540	0.8119800840755560	0.4663880747730142
0.3119800840755561	0.4088123845306461	0.0336119252269860
0.4094773292901673	0.9655546513337685	0.6823978058294609
0.8197186953183979	0.9086457224035002	0.5347963637805974
0.5344453486662315	0.0905226707098327	0.1823978058294611
0.0911876154693540	0.1880199159244439	0.5336119252269861
0.3157690632498529	0.9657826591216611	0.5900640411408976
0.5342173408783389	0.8157690632498524	0.9099359588591024
0.4657826591216609	0.1842309367501472	0.9099359588591024
0.4094773292901673	0.0344453486662317	0.3176021941705386
0.5905226707098327	0.9655546513337685	0.3176021941705386
0.5913542775964998	0.6802813046816021	0.0347963637805974
0.4086457224035001	0.6802813046816021	0.9652036362194026
0.5342173408783389	0.1842309367501472	0.0900640411408977
0.4086457224035001	0.3197186953183980	0.0347963637805974
0.6842309367501476	0.0342173408783390	0.5900640411408976
0.6880199159244440	0.5911876154693543	0.0336119252269860
0.6880199159244440	0.4088123845306461	0.9663880747730139
0.1802813046816019	0.9086457224035002	0.4652036362194025
0.6842309367501476	0.9657826591216611	0.4099359588591027
0.3157690632498529	0.0342173408783390	0.4099359588591027
0.4657826591216609	0.8157690632498524	0.0900640411408977
0.1802813046816019	0.0913542775964999	0.5347963637805974

Remove four ligands

```
   1.00000000000000
    16.9955997467000017    0.0000000000000000    0.0000000000000000
     0.0000000000000000   16.9955997467000017    0.0000000000000000
     0.0000000000000000    0.0000000000000000   16.9955997467000017
   N    C    H    Zn
    16    32    40     8
Direct
  0.5925181845908920  0.9657075290475822  0.3140937141796754
  0.4074818154091079  0.0342924889524120  0.3140937141796754
  0.4074818154091079  0.9657075290475822  0.6859062858203245
  0.5925181845908920  0.0342924889524120  0.6859062858203245
  0.3152972407804509  0.5926702229320163  0.9653942301288559
  0.3152972407804509  0.4073297770679836  0.0346057878711380
  0.6847027592195488  0.4073297770679836  0.9653942301288559
  0.6847027592195488  0.5926702229320163  0.0346057878711380
  0.5925181845908920  0.3140937141796754  0.9657075290475822
  0.4074818154091079  0.3140937141796754  0.0342924889524120
  0.4074818154091079  0.6859062858203245  0.9657075290475822
  0.5925181845908920  0.6859062858203245  0.0342924889524120
  0.3152972407804509  0.9653942301288559  0.5926702229320163
  0.3152972407804509  0.0346057878711380  0.4073297770679836
  0.6847027592195488  0.9653942301288559  0.4073297770679836
  0.6847027592195488  0.0346057878711380  0.5926702229320163
  0.6346514055927441  0.8963354354906684  0.3076421118413232
  0.3653485644072603  0.1036645415093260  0.3076421118413232
  0.3653485644072603  0.8963354354906684  0.6923578881586764
  0.6346514055927441  0.1036645415093260  0.6923578881586764
  0.3085741004522528  0.6349430707651078  0.8961281980029555
  0.3085741004522528  0.3650568992348970  0.1038717789970389
  0.6914258995477468  0.3650568992348970  0.8961281980029555
  0.6914258995477468  0.6349430707651078  0.1038717789970390
  0.6346514055927441  0.3076421118413232  0.8963354354906684
  0.3653485644072603  0.3076421118413232  0.1036645415093260
  0.3653485644072603  0.6923578881586764  0.8963354354906684
  0.6346514055927441  0.6923578881586764  0.1036645415093260
  0.3085741004522528  0.8961281980029555  0.6349430707651078
  0.3085741004522528  0.1038717789970390  0.3650568992348970
  0.6914258995477468  0.8961281980029555  0.3650568992348970
  0.6914258995477468  0.1038717789970390  0.6349430707651078
  0.6246268979917986  0.0060497244716808  0.3747283395720371
```

0.5966875622581553	0.0844088314237699	0.4026809227527838
0.3753730720082062	0.9939502595283252	0.3747283395720371
0.4033124377418449	0.9155911835762279	0.4026809227527838
0.3753730720082062	0.0060497244716808	0.6252716304279675
0.4033124377418449	0.0844088314237699	0.5973190772472162
0.6246268979917986	0.9939502595283252	0.6252716304279674
0.5966875622581553	0.9155911835762279	0.5973190772472162
0.3753730720082062	0.6252716304279674	0.0060497244716808
0.4033124377418449	0.5973190772472162	0.0844088314237699
0.3753730720082062	0.3747283395720370	0.9939502595283252
0.4033124377418449	0.4026809227527838	0.9155911835762279
0.6246268979917986	0.3747283395720370	0.0060497244716808
0.5966875622581553	0.4026809227527838	0.0844088314237699
0.6246268979917986	0.6252716304279675	0.9939502595283252
0.5966875622581553	0.5973190772472162	0.9155911835762279
0.6213214030858784	0.8532921408782773	0.2622673215327741
0.3786786269141241	0.1467078881217218	0.2622673215327741
0.3786786269141241	0.8532921408782773	0.7377326784672259
0.6213214030858784	0.1467078881217219	0.7377326784672259
0.2635568064646013	0.6213121503033913	0.8528029999177995
0.3545500560562324	0.8815099407017150	0.4280511717069065
0.2635568064646013	0.3786878796966111	0.1471970290822001
0.3545500560562324	0.1184900442982870	0.5719488282930933
0.7364431935353987	0.3786878796966111	0.8528029999177995
0.6454499439437676	0.1184900442982870	0.4280511717069065
0.7364431935353987	0.6213121503033913	0.1471970290822001
0.6454499439437676	0.8815099407017151	0.5719488282930933
0.4286765069591886	0.3539588680632446	0.8814817937281118
0.5713234930408121	0.3539588680632446	0.1185181912718906
0.4286765069591886	0.6460411319367559	0.1185181912718906
0.5713234930408122	0.6460411319367559	0.8814817937281118
0.4286765069591886	0.8814817937281118	0.3539588680632446
0.5713234930408121	0.1185181912718906	0.3539588680632446
0.4286765069591886	0.1185181912718906	0.6460411319367559
0.5713234930408121	0.8814817937281118	0.6460411319367559
0.6213214030858784	0.2622673215327741	0.8532921408782773
0.3786786269141241	0.2622673215327741	0.1467078881217218
0.3786786269141241	0.7377326784672259	0.8532921408782773
0.6213214030858784	0.7377326784672259	0.1467078881217218
0.2635568064646013	0.8528029999177995	0.6213121503033913
0.3545500560562324	0.4280511717069065	0.8815099407017151

0.2635568064646013	0.1471970290822001	0.3786878796966111
0.3545500560562324	0.5719488282930933	0.1184900442982870
0.7364431935353987	0.8528029999177995	0.3786878796966111
0.6454499439437676	0.4280511717069065	0.1184900442982870
0.7364431935353987	0.1471970290822001	0.6213121503033913
0.6454499439437676	0.5719488282930933	0.8815099407017151
0.4489553772541408	0.9212103950687331	0.4483722855777241
0.4489553772541408	0.0787896049312670	0.5516277444222782
0.5510446527458619	0.0787896049312670	0.4483722855777241
0.5510446527458619	0.9212103950687331	0.5516277444222782
0.4489553772541408	0.4483722855777241	0.9212103950687331
0.5510446527458619	0.4483722855777241	0.0787896049312670
0.4489553772541408	0.5516277444222782	0.0787896049312670
0.5510446527458619	0.5516277444222782	0.9212103950687331
0.5000000000000000	0.0000000000000000	0.2446173155141393
0.5000000000000000	0.0000000000000000	0.7553826844858607
0.2454776041113712	0.5000000000000000	0.0000000000000000
0.7545223958886288	0.5000000000000000	0.0000000000000000
0.2454776041113712	0.0000000000000000	0.5000000000000000
0.5000000000000000	0.2446173155141393	0.0000000000000000
0.5000000000000000	0.7553826844858607	0.0000000000000000
0.7545223958886288	0.0000000000000000	0.5000000000000000

One ligand-cluster

```
   1.00000000000000
    16.9955999999999996    0.0000000000000000    0.0000000000000000
     0.0000000000000000   16.9955999999999996    0.0000000000000000
     0.0000000000000000    0.0000000000000000   16.9955999999999996
   H    C    Zn   N
    20   16    1    8
Direct
  0.6456385730224361  0.7331678944529249  0.3629674558711962
  0.3543614269775637  0.2668321055470751  0.3629674558711962
  0.7331678944529249  0.3543614269775637  0.6370325441288029
  0.2668321055470751  0.6456385730224361  0.6370325441288029
  0.6441760740235807  0.6136569604497766  0.6944350429494710
  0.3558239259764192  0.3863430395502235  0.6944350429494710
  0.6136569604497766  0.3558239259764192  0.3055649570505291
  0.3863430395502235  0.6441760740235807  0.3055649570505291
  0.5462347288059018  0.5776342915083440  0.7006958230073866
  0.4537652711940981  0.4223657084916561  0.7006958230073866
  0.5776342915083440  0.4537652711940981  0.2993041769926125
  0.4223657084916561  0.5462347288059018  0.2993041769926125
  0.6231853520800710  0.4223324104466470  0.3873116091522767
  0.3768146479199291  0.5776675895533533  0.3873116091522767
  0.4223324104466470  0.3768146479199291  0.6126883908477224
  0.5776675895533533  0.6231853520800710  0.6126883908477224
  0.6186198698686537  0.3550743100169458  0.5198965519578442
  0.3813801301313463  0.6449256899830543  0.5198965519578442
  0.3550743100169458  0.3813801301313463  0.4801034480421555
  0.6449256899830543  0.6186198698686537  0.4801034480421555
  0.6003417544350668  0.6898371208324340  0.3760573998259590
  0.3996582455649341  0.3101628791675661  0.3760573998259590
  0.6898371208324340  0.3996582455649341  0.6239426001740402
  0.3101628791675661  0.6003417544350668  0.6239426001740402
  0.5846697196472855  0.4027913186807076  0.3390514168786704
  0.4153302803527143  0.5972086813192922  0.3390514168786704
  0.4027913186807076  0.4153302803527143  0.6609485831213296
  0.5972086813192922  0.5846697196472855  0.6609485831213296
  0.6320346354094089  0.3994585376702043  0.5640083730685455
  0.3679653645905914  0.6005414623297954  0.5640083730685455
  0.3994585376702043  0.3679653645905914  0.4359916269314545
  0.6005414623297954  0.6320346354094089  0.4359916269314545
  0.5072628193988049  0.3749882899217005  0.3680530900410984
```

0.4927371806011951	0.6250117100783003	0.3680530900410984
0.3749882899217005	0.4927371806011951	0.6319469099589022
0.6250117100783003	0.5072628193988049	0.6319469099589022
0.5000000000000000	0.5000000000000000	0.5000000000000000
0.5911399860710085	0.4679113481980152	0.5691966002437303
0.4088600139289920	0.5320886518019849	0.5691966002437303
0.4679113481980152	0.4088600139289920	0.4308033997562699
0.5320886518019849	0.5911399860710085	0.4308033997562699
0.5328454261418135	0.6850616080992683	0.3344214034825100
0.4671545738581868	0.3149383919007315	0.3344214034825100
0.6850616080992683	0.4671545738581868	0.6655785965174895
0.3149383919007315	0.5328454261418135	0.6655785965174895

Original ZIF-8 structure

```
1.00000000000000
  16.9955997467000017    0.0000000000000000    0.0000000000000000
   0.0000000000000000   16.9955997467000017    0.0000000000000000
   0.0000000000000000    0.0000000000000000   16.9955997467000017
H   C   Zn   N
120   96   12   48
Direct
0.6158747958289833  0.8513112965625588  0.2686276267690848
0.3841524327891851  0.1487625650090563  0.2688224263331025
0.3840638821611955  0.8511811135431737  0.7312948779846264
0.6158668607233412  0.1489438562331686  0.7310436921250609
0.2687317525332522  0.6159915248408767  0.8512718650314264
0.2687773076180244  0.3842056809868730  0.1488394018042499
0.7313320486118725  0.3839997068093736  0.8512591044089642
0.7311076464025814  0.6157667398288591  0.1488631018722778
0.8514141795947708  0.2685360306816887  0.6158956633351342
0.1488604254042478  0.2689030112184186  0.3842718653191092
0.8509885347191414  0.7309936238965029  0.3841228844445048
0.1487154743799355  0.7313936975801354  0.6160659633855154
0.8514141795947708  0.6158956633351342  0.2685360306816887
0.1488604254042478  0.3842718653191092  0.2689030112184186
0.8509885347191414  0.3841228844445048  0.7309936238965029
0.1487154743799355  0.6160659633855154  0.7313936975801354
0.6158747958289833  0.2686276267690848  0.8513112965625588
0.3841524327891851  0.2688224263331025  0.1487625650090563
0.3840638821611955  0.7312948779846264  0.8511811135431737
0.6158668607233412  0.7310436921250609  0.1489438562331686
0.2687317525332522  0.8512718650314264  0.6159915248408767
0.2687773076180244  0.1488394018042499  0.3842056809868730
0.7313320486118725  0.8512591044089642  0.3839997068093736
0.7311076464025814  0.1488631018722778  0.6157667398288591
0.1158747738289887  0.3513112665625634  0.7686275967690827
0.8841524027891896  0.6487625350090608  0.7688223963331000
0.8840638521612000  0.3511810835431786  0.2312948479846238
0.1158668387233466  0.6489438262331734  0.2310436621250586
0.7687317225332500  0.1159915028408819  0.3512718350314311
0.7687772776180217  0.8842056509868776  0.6488393718042544
0.2313320186118701  0.8839996768093781  0.3512590744089686
0.2311076164025785  0.1157667178288643  0.6488630718722825
0.3514141495947752  0.7685360006816864  0.1158956413351394
```

0.6488603954042522	0.7689029812184162	0.8842718353191140
0.3509885047191461	0.2309935938965003	0.8841228544445096
0.6487154443799400	0.2313936675801330	0.1160659413855208
0.3514141495947752	0.1158956413351394	0.7685360006816864
0.6488603954042522	0.8842718353191140	0.7689029812184162
0.3509885047191461	0.8841228544445096	0.2309935938965003
0.6487154443799400	0.1160659413855208	0.2313936675801330
0.1158747738289887	0.7686275967690827	0.3513112665625634
0.8841524027891896	0.7688223963331000	0.6487625350090608
0.8840638521612000	0.2312948479846238	0.3511810835431786
0.1158668387233466	0.2310436621250586	0.6489438262331734
0.7687317225332500	0.3512718350314311	0.1159915028408819
0.7687772776180217	0.6488393718042544	0.8842056509868776
0.2313320186118701	0.3512590744089686	0.8839996768093781
0.2311076164025785	0.6488630718722825	0.1157667178288643
0.9176465118028899	0.3752312179554544	0.8552792052115302
0.0520935953971108	0.5784212250611857	0.9526118930436644
0.9068438450972964	0.5870402826190938	0.0307122714279269
0.1232231372691144	0.3661895970779390	0.1278512252601821
0.3806722194579398	0.8564913416122075	0.9305623414761861
0.5822286552546884	0.9233921414853321	0.0334003768396515
0.6299655391824283	0.0985935347869862	0.8580143577945522
0.4079572715256415	0.0320756929328143	0.1063203101877587
0.8607203824268665	0.9424393364795014	0.3867969471012087
0.9635453881609223	0.1183251604591233	0.5978024767157345
0.0666499884644868	0.8571872431031613	0.6180107359821732
0.1091855270368509	0.0328077155987580	0.4066987102816197
0.4176465118028896	0.8752311879554594	0.3552792052115306
0.5520935883971069	0.0784212180611814	0.4526118930436648
0.4068438450972965	0.0870402756190895	0.5307122994279294
0.6232231222691166	0.8661895670779438	0.6278512102601772
0.8806722194579396	0.3564913416122079	0.4305623414761864
0.0822286322546827	0.4233921414853323	0.5334003808396520
0.1299655241824306	0.5985935117869877	0.3580143277945497
0.9079572415256459	0.5320756819328099	0.6063203401877609
0.3607203824268664	0.4424393664794968	0.8867969471012085
0.4635453581609272	0.6183251524591229	0.0978024537157363
0.5666500114644851	0.3571872431031613	0.1180107289821689
0.6091855420368484	0.5328077435987605	0.9066986802816173
0.3806722194579398	0.9305623414761861	0.8564913416122075
0.5822286552546884	0.0334003768396515	0.9233921414853321

0.6299655391824283	0.8580143577945522	0.0985935347869862
0.4079572715256415	0.1063203101877587	0.0320756929328143
0.8607203824268665	0.3867969471012087	0.9424393364795014
0.9635453881609223	0.5978024767157345	0.1183251604591233
0.0666499884644868	0.6180107359821732	0.8571872431031613
0.1091855270368509	0.4066987102816197	0.0328077155987580
0.9176465118028899	0.8552792052115302	0.3752312179554544
0.0520935953971108	0.9526118930436644	0.5784212250611857
0.9068438450972964	0.0307122714279269	0.5870402826190938
0.1232231372691144	0.1278512252601821	0.3661895970779390
0.9593898785838973	0.4207918632283426	0.9390452032304919
0.1440516438304327	0.6206439711865129	0.9277304942709756
0.8652874601562128	0.6330975903757753	0.1141974350976065
0.0314961326398615	0.4091305669901078	0.1033814408228892
0.4215553334774252	0.9503355913247304	0.9503355913247304
0.6053974081196356	0.9546541145490998	0.1305010517922586
0.6053974081196356	0.1305010517922586	0.9546541145490998
0.3661132113517042	0.1255844506800944	0.1255844506800944
0.9593898785838973	0.9390452032304919	0.4207918632283426
0.8652874601562128	0.1141974350976065	0.6330975903757752
0.1440516438304327	0.9277304942709756	0.6206439711865129
0.0314961326398615	0.1033814408228892	0.4091305669901078
0.8806722194579396	0.4305623414761864	0.3564913416122079
0.0822286322546827	0.5334003808396520	0.4233921414853323
0.1299655241824306	0.3580143277945497	0.5985935117869877
0.9079572415256459	0.6063203401877609	0.5320756819328099
0.3607203824268664	0.8867969471012085	0.4424393664794968
0.4635453581609272	0.0978024537157363	0.6183251524591229
0.5666500114644851	0.1180107289821689	0.3571872431031613
0.6091855420368484	0.9066986802816173	0.5328077435987605
0.4176465118028896	0.3552792052115306	0.8752311879554594
0.5520935883971069	0.4526118930436648	0.0784212180611814
0.4068438450972965	0.5307122994279294	0.0870402756190895
0.6232231222691166	0.6278512102601772	0.8661895670779438
0.4593898785838971	0.9207918632283423	0.4390452332304944
0.6440516138304300	0.1206439631865125	0.4277304942709754
0.3652874901562083	0.1330976053757728	0.6141974500976045
0.5314961366398621	0.9091305369901121	0.6033814258228846
0.9215553334774248	0.4503355913247302	0.4503355913247302
0.1053974151196325	0.4546540855491012	0.6305010517922585
0.1053974151196325	0.6305010517922585	0.4546540855491012

0.8661131813517018	0.6255844206800989	0.6255844206800989
0.4593898785838971	0.4390452332304944	0.9207918632283423
0.3652874901562083	0.6141974500976045	0.1330976053757728
0.6440516138304300	0.4277304942709754	0.1206439631865125
0.5314961366398621	0.6033814258228846	0.9091305369901121
0.5953272335603756	0.0849805693859637	0.4047118663349541
0.4047545955390772	0.9150153729499483	0.4046149524157444
0.4045721931807549	0.0851206866184604	0.5953386789382970
0.5953714813063689	0.9149043577556718	0.5953832803728423
0.4045721931807549	0.5953386789382970	0.0851206866184604
0.4047545955390772	0.4046149524157444	0.9150153729499483
0.5953272335603756	0.4047118663349541	0.0849805693859637
0.5953714813063689	0.5953832803728423	0.9149043577556718
0.0850683119366569	0.4045546231063038	0.5953094455945375
0.9150334540345655	0.4047033254783443	0.4047033254783443
0.0850683119366569	0.5953094455945375	0.4045546231063038
0.9148913770790374	0.5953833538640816	0.5953833538640816
0.0953272415603763	0.5849805543859661	0.9047118663349540
0.9047545955390771	0.4150153429499460	0.9046149524157446
0.9045721931807550	0.5851206716184628	0.0953386869382974
0.0953714893063696	0.4149043277556695	0.0953832883728428
0.9045721931807550	0.0953386869382974	0.5851206716184628
0.9047545955390771	0.9046149524157446	0.4150153429499460
0.0953272415603763	0.9047118663349540	0.5849805543859661
0.0953714893063696	0.0953832883728428	0.4149043277556695
0.5850682969366593	0.9045546231063036	0.0953094535945380
0.4150334240345629	0.9047033254783441	0.9047033254783441
0.5850682969366593	0.0953094535945380	0.9045546231063036
0.4148913470790347	0.0953833618640821	0.0953833618640821
0.6308610853797746	0.8957821133094779	0.3119223733462326
0.3690512854373683	0.1042232876598089	0.3120007625226317
0.3689182866009449	0.8958782228770122	0.6882781744354343
0.6311372986551551	0.1041336516810433	0.6881924687796941
0.3118001228965032	0.6311456166215368	0.8959211759593283
0.3119192738928802	0.3691294622556187	0.1042583887177406
0.6880763641064446	0.3689697567283658	0.8957710012142637
0.6881808292419948	0.6309867607866231	0.1041269456254558
0.8959103887971926	0.3117800397054469	0.6309563888566156
0.1042722034436095	0.3120156228140425	0.3691472055679428
0.8957487276104147	0.6880610541638991	0.3689303889681536
0.1040722107846015	0.6882994073911582	0.6311700744180778

0.8959103887971926	0.6309563888566156	0.3117800397054469
0.1042722034436095	0.3691472055679428	0.3120156228140425
0.8957487276104147	0.3689303889681536	0.6880610541638991
0.1040722107846015	0.6311700744180778	0.6882994073911582
0.6308610853797746	0.3119223733462326	0.8957821133094779
0.3690512854373683	0.3120007625226317	0.1042232876598089
0.3689182866009449	0.6882781744354343	0.8958782228770122
0.6311372986551551	0.6881924687796941	0.1041336516810433
0.3118001228965032	0.8959211759593283	0.6311456166215368
0.3119192738928802	0.1042583887177406	0.3691294622556187
0.6880763641064446	0.8957710012142637	0.3689697567283658
0.6881808292419948	0.1041269456254558	0.6309867607866231
0.1308611153797699	0.3957821433094804	0.8119223733462323
0.8690513154373636	0.6042233106598143	0.8120007625226319
0.8689183166009402	0.3958782528770148	0.1882781744354342
0.1311373286551505	0.6041336746810489	0.1881924687796940
0.8118001228965032	0.1311456466215322	0.3959212059593306
0.8119192738928801	0.8691294922556145	0.6042584117177463
0.1880763641064447	0.8689697867283613	0.3957710312142660
0.1881808292419946	0.1309867907866184	0.6041269686254613
0.3959104187971951	0.8117800397054471	0.1309564188566111
0.6042722264436152	0.8120156228140425	0.8691472355679379
0.3957487576104172	0.1880610541638992	0.8689304189681489
0.6040722337846068	0.1882994073911583	0.1311701044180730
0.3959104187971951	0.1309564188566111	0.8117800397054471
0.6042722264436152	0.8691472355679379	0.8120156228140425
0.3957487576104172	0.8689304189681489	0.1880610541638992
0.6040722337846068	0.1311701044180730	0.1882994073911583
0.1308611153797699	0.8119223733462323	0.3957821433094804
0.8690513154373636	0.8120007625226319	0.6042233106598143
0.8689183166009402	0.1882781744354342	0.3958782528770148
0.1311373286551505	0.1881924687796940	0.6041336746810489
0.8118001228965032	0.3959212059593306	0.1311456466215322
0.8119192738928801	0.6042584117177463	0.8691294922556145
0.1880763641064447	0.3957710312142660	0.8689697867283613
0.1881808292419946	0.6041269686254613	0.1309867907866184
0.6236830393516455	0.0068525174665058	0.3762820059973392
0.3763737368068274	0.9932304379464633	0.3764320555494668
0.3769660396929879	0.0064661306923086	0.6230917800645768
0.6229484446912767	0.9936433195615959	0.6229199488249716
0.3769660396929879	0.6230917800645768	0.0064661306923086

0.3763737368068274	0.3764320555494668	0.9932304379464633
0.6236830393516455	0.3762820059973392	0.0068525174665058
0.6229484446912767	0.6229199488249716	0.9936433195615959
0.0066177161952276	0.3766894553613035	0.6233502008093040
0.9931818537603178	0.3763271183268009	0.3763271183268009
0.0066177161952276	0.6233502008093040	0.3766894553613035
0.9936122670030436	0.6229603491530713	0.6229603491530713
0.1236830693516407	0.5068525334665002	0.8762820359973347
0.8763737668068229	0.4932304679464585	0.8764320855494622
0.8769660696929832	0.5064661466923027	0.1230918100645721
0.1229484746912721	0.4936433495615913	0.1229199788249668
0.8769660696929832	0.1230918100645721	0.5064661466923027
0.8763737668068229	0.8764320855494622	0.4932304679464585
0.1236830693516407	0.8762820359973347	0.5068525334665002
0.1229484746912721	0.1229199788249668	0.4936433495615913
0.5066177321952219	0.8766894853612990	0.1233502308092998
0.4931818837603132	0.8763271483267960	0.8763271483267960
0.5066177321952219	0.1233502308092998	0.8766894853612990
0.4936122970030392	0.1229603791530666	0.1229603791530666
0.4998346766493414	-0.0000049318727683	0.2502207802342627
0.5001446875816227	-0.0000188076432620	0.7502236279534030
0.2500038503658503	0.5000699705263236	0.0000468347226436
0.7500038503658500	0.5000468347226437	0.0000699705263237
0.0001446875816228	0.2502236279534027	0.4999811923567381
-0.0001653233506586	0.7502207802342625	0.4999950681272318
0.0001446875816228	0.4999811923567381	0.2502236279534027
-0.0001653233506586	0.4999950681272318	0.7502207802342625
0.4998346766493414	0.2502207802342627	-0.0000049318727683
0.5001446875816227	0.7502236279534030	-0.0000188076432620
0.2500038503658503	0.0000468347226436	0.5000699705263236
0.7500038503658500	0.0000699705263237	0.5000468347226437
0.5904841291406522	0.9660774369873112	0.3166493289926523
0.4094371686926439	0.0339544208095224	0.3166878616941788
0.4098222102567494	0.9658828559841252	0.6830978985957920
0.5902202928220398	0.0341610435139916	0.6830412694161616
0.3169574041786090	0.5902873862970411	0.9659030364345508
0.3166401464546297	0.4095603534474780	0.0339724874556737
0.6834685695689738	0.4093398835081767	0.9661210173066478
0.6829205253907492	0.5900578911494571	0.0342169897031660
0.9660375066469502	0.3167697802821056	0.5903887325283254
0.0339580002175998	0.3166476597997694	0.4094764844184026

0.9658921313128401	0.6831784055549027	0.4096565738108313
0.0341292535868102	0.6830489410122238	0.5902023643560440
0.9660375066469502	0.5903887325283254	0.3167697802821056
0.0339580002175998	0.4094764844184026	0.3166476597997694
0.9658921313128401	0.4096565738108313	0.6831784055549027
0.0341292535868102	0.5902023643560440	0.6830489410122238
0.5904841291406522	0.3166493289926523	0.9660774369873112
0.4094371686926439	0.3166878616941788	0.0339544208095224
0.4098222102567494	0.6830978985957920	0.9658828559841252
0.5902202928220398	0.6830412694161616	0.0341610435139916
0.3169574041786090	0.9659030364345508	0.5902873862970411
0.3166401464546297	0.0339724874556737	0.4095603534474780
0.6834685695689738	0.9661210173066478	0.4093398835081767
0.6829205253907492	0.0342169897031660	0.5900578911494571
0.0904841291406523	0.4660774079873125	0.8166493289926523
0.9094371686926437	0.5339544028095279	0.8166878616941788
0.9098222102567496	0.4658828269841260	0.1830978985957918
0.0902202928220398	0.5341610255139972	0.1830412694161616
0.8169574041786090	0.0902873862970411	0.4659030074345519
0.8166401464546296	0.9095603534474781	0.5339724694556794
0.1834685695689737	0.9093398835081765	0.4661209883066487
0.1829205253907489	0.0900578911494570	0.5342169717031718
0.4660374776469516	0.8167697802821055	0.0903887325283254
0.5339579822176055	0.8166476597997696	0.9094764844184026
0.4658921023128414	0.1831784055549026	0.9096565738108312
0.5341292355868160	0.1830489410122237	0.0902023643560443
0.4660374776469516	0.0903887325283254	0.8167697802821055
0.5339579822176055	0.9094764844184026	0.8166476597997696
0.4658921023128414	0.9096565738108312	0.1831784055549026
0.5341292355868160	0.0902023643560443	0.1830489410122237
0.0904841291406523	0.8166493289926523	0.4660774079873125
0.9094371686926437	0.8166878616941788	0.5339544028095279
0.9098222102567496	0.1830978985957918	0.4658828269841260
0.0902202928220398	0.1830412694161616	0.5341610255139972
0.8169574041786090	0.4659030074345519	0.0902873862970411
0.8166401464546296	0.5339724694556794	0.9095603534474781
0.1834685695689737	0.4661209883066487	0.9093398835081765
0.1829205253907489	0.5342169717031718	0.0900578911494570
